# A Novel Metabolomic Aging Clock Predicting Health Outcomes and Its Genetic and Modifiable Factors

**DOI:** 10.1002/advs.202406670

**Published:** 2024-09-27

**Authors:** Xueqing Jia, Jiayao Fan, Xucheng Wu, Xingqi Cao, Lina Ma, Zeinab Abdelrahman, Fei Zhao, Haitao Zhu, Daniele Bizzarri, Erik B van den Akker, P. Eline Slagboom, Joris Deelen, Dan Zhou, Zuyun Liu

**Affiliations:** ^1^ Center for Clinical Big Data and Analytics of the Second Affiliated Hospital and Department of Big Data in Health Science School of Public Health, Zhejiang Key Laboratory of Intelligent Preventive Medicine Zhejiang University School of Medicine Hangzhou 310058 China; ^2^ Department of General Practice, Sir Run Run Shaw Hospital Zhejiang University School of Medicine Hangzhou 310016 China; ^3^ Department of Geriatrics National Clinical Research Center for Geriatric Medicine Xuanwu Hospital Capital Medical University Beijing 100053 China; ^4^ Molecular Epidemiology and Public Health Research Group, Centre for Public Health, Queen’s University Belfast, Institute for Clinical Sciences A, Royal Victoria Hospital Belfast BT12 6BA UK; ^5^ Hangzhou Meilian Medical Co., Ltd. Hangzhou 311200 China; ^6^ Molecular Epidemiology Department of Biomedical Data Sciences Leiden University Medical Center Leiden 2333 ZC The Netherlands; ^7^ The Delft Bioinformatics Lab, Pattern Recognition & Bioinformatics Delft University of Technology Delft 2628 CC The Netherlands; ^8^ Max Planck Institute for Biology of Ageing 50931 Cologne Germany; ^9^ Cologne Excellence Cluster on Cellular Stress Responses in Ageing‐Associated Diseases (CECAD) University of Cologne 50931 Cologne Germany

**Keywords:** aging, biological age, genetic determinant, metabolomic, modifiable factor, mortality

## Abstract

Existing metabolomic clocks exhibit deficiencies in capturing the heterogeneous aging rates among individuals with the same chronological age. Yet, the modifiable and non‐modifiable factors in metabolomic aging have not been systematically studied. Here, a new aging measure—MetaboAgeMort—is developed using metabolomic profiles from 239,291 UK Biobank participants for 10‐year all‐cause mortality prediction. The MetaboAgeMort showed significant associations with all‐cause mortality, cause‐specific mortality, and diverse incident diseases. Adding MetaboAgeMort to a conventional risk factors model improved the predictive ability of 10‐year mortality. A total of 99 modifiable factors across seven categories are identified for MetaboAgeMort. Among these, 16 factors representing pulmonary function, body composition, socioeconomic status, dietary quality, smoking status, alcohol intake, and disease status showed quantitatively stronger associations. The genetic analyses revealed 99 genomic risk loci and 271 genes associated with MetaboAgeMort. The tissue‐enrichment analysis showed significant enrichment in liver. While the external validation of the MetaboAgeMort is required, this study illuminates heterogeneous metabolomic aging across the same age, providing avenues for identifying high‐risk individuals, developing anti‐aging therapies, and personalizing interventions, thus promoting healthy aging and longevity.

## Graphical abstract

The new aging measure—MetaboAgeMort—shows significant associations with all‐cause mortality, cause‐specific mortality, and diverse incident diseases. Adding MetaboAgeMort to a conventional risk factors model improves the predictive ability of 10‐year mortality. A total of 99 modifiable factors across seven key domains are identified for MetaboAgeMort. The genetic analyses reveal 99 genomic risk loci and 271 genes associated with MetaboAgeMort.

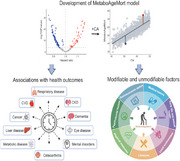

## 1 Introduction

Individuals of the same chronological age may have varying rates of biological aging, a phenomenon known as heterogeneous aging. Such different rate of aging engenders diverse susceptibilities to diseases, their progression, and impacts on mortality beyond the chronological age.^[^ ^1^, ^2^ ^]^ As the global demographic shifts toward an aging population, the ability to measure biological aging, identify individuals who age faster, and understand the factors that contribute to differential rates of aging are of utmost importance. These findings have significant implications for the development of targeted preventive programs and interventions. These efforts may alleviate the socioeconomic and healthcare burden of age‐related diseases, thus promoting healthy aging and longevity.

Metabolomics offers a novel avenue for assessing the biological processes that underlie aging.^[^ ^3^, ^4^ ^]^ To date, multiple studies have attempted to elucidate how metabolomic profiles in various tissues (e.g., blood, urine, and cerebrospinal fluid) interact with aging, and a few metabolomic clocks have been proposed to measure biological aging.^[^ ^5^, ^6^, ^7^, ^8^ ^]^ The majority of these clocks are generated based on correlations between metabolomic profiles and chronological age; while chronological age is considered an imperfect surrogate for building aging measures as it does not fully capture the heterogeneity of individual aging rates.^[^ ^9^ ^]^ In contrast, metabolomic aging measures based on health‐related surrogate indicators (e.g., time to death) may better reflect an individual's health status and reveal intrinsic biological aging mechanisms.^[^ ^10^ ^]^ Previous studies have utilized targeted or untargeted mass spectrometry and NMR techniques to construct multivariable metabolite scores of all‐cause mortality.^[^ ^11^, ^12^, ^13^ ^]^ ​While these metabolite scores exhibit exceptional predictive accuracy even over conventional risk factor models, their applicability in risk stratification, especially across individuals of the same chronological age, remains constrained by their reliance on scaled biomarker values created independently for each cohort.^[^ ^13^ ^]^ Metabolomic profiles manifest pronounced responsiveness to the confluence of endogenous genetic regulation and exogenous environmental exposures in each individual cohort.^[^ ^14^ ^]^ Thus, developing aging measures that can be calculated based on concentration units derived from individual‐level data, may have more applicability in both clinical setting and research on the biology of aging.

Although certain behaviors (e.g., dietary quality) have displayed anti‐aging properties in human and animal models,^[^ ^15^, ^16^ ^]^ their impact on metabolomic aging remains uncertain. Furthermore, the complex relationship between aging and a constellation of diverse factors (e.g., local environmental factors and socioeconomic status [SES]) has emerged as a burgeoning focus in the field of aging research. A thorough investigation of these modifiable factors in metabolomic aging could reveal new strategies for preventive interventions targeting the aging process. Moreover, prior investigations have indicated that aging measures may capture distinct aging domains influenced by varying genetic determinants.^[^ ^17^ ^]^ However, the underlying mechanisms and pathways of metabolomic aging have not been elucidated, thereby restricting the identification of potential therapeutic targets.^[^ ^18^ ^]^

**Figure 1:**
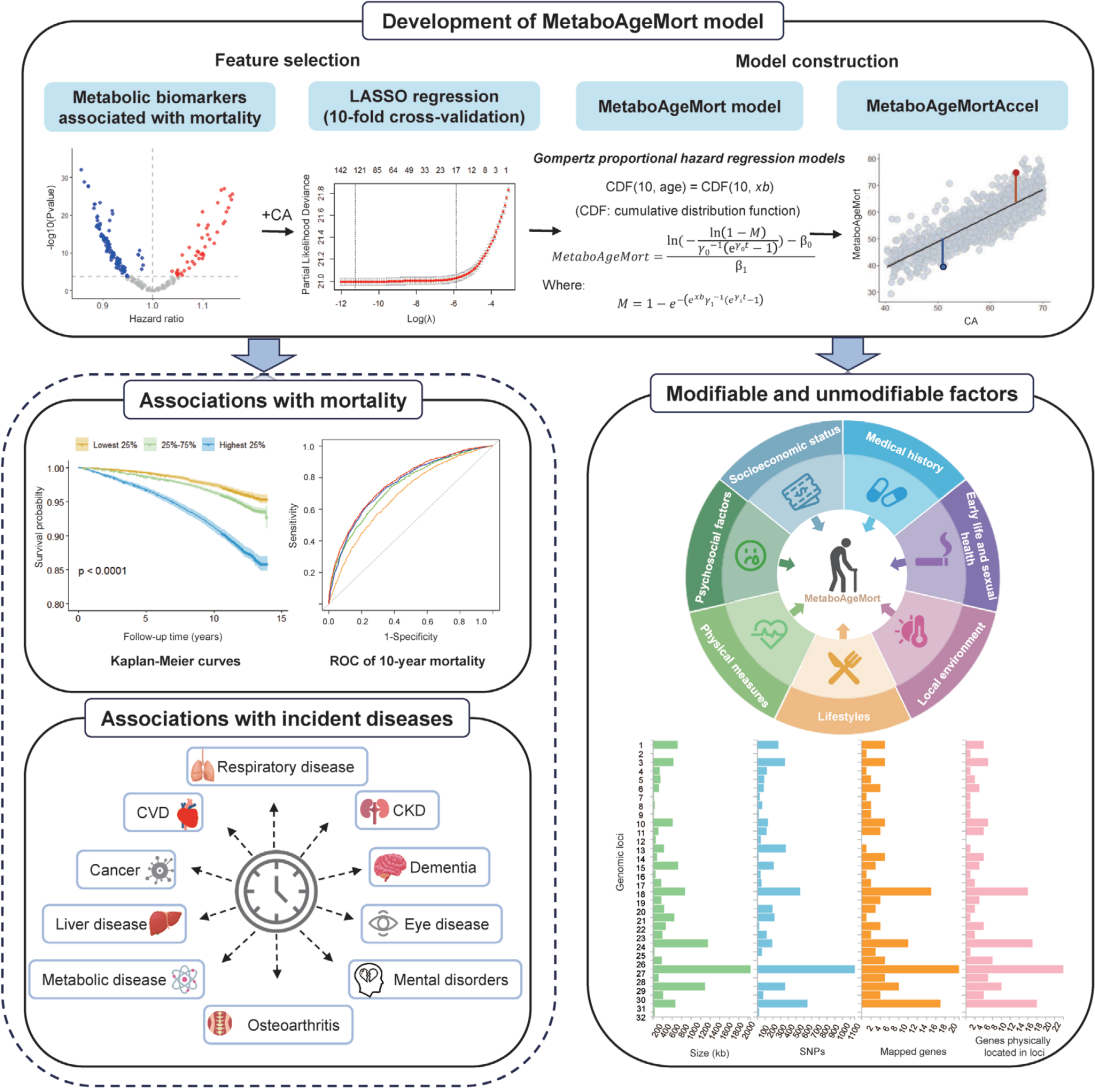
The roadmap of this study. Top part, the development of MetaboAgeMort model. We performed two steps to develop the MetaboAgeMort model. First, we selected 185 all‐cause mortality associated metabolic biomarkers and then performed LASSO Cox regression model to further select variables for MetaboAgeMort construction. Tenfold cross‐validation was used to select the optimal λ. Second, we developed MetaboAgeMort model using Gompertz proportional hazards regression models. Then we calculated the residual resulting from a least‐squares linear model when regressing MetaboAgeMort on CA, and referred to as MetaboAgeMortAccel. Bottom left part, the associations of MetaboAgeMort with mortality and incident diseases. Metabolic diseases included hypertension and type 2 diabetes mellitus. Mental disorders included depression and anxiety. ROC curves were used to evaluate the additional predictive power of MetaboAgeMort for 10‐year mortality beyond the conventional risk factors. Bottom right part, the modifiable and unmodifiable factors for MetaboAgeMort. We identified the modifiable factors across seven categories and genomic risk loci for MetaboAgeMort. CA, chronological age; LASSO, least absolute shrinkage and selection operator; MetaboAgeMortAccel, MetaboAgeMort acceleration; ROC, receiver operating characteristic; CVD, cardiovascular disease; CKD, chronic kidney disease; SNPs, single nucleotide polymorphisms.

In this study, we leveraged large‐scale metabolomic data from the UK Biobank (UKB), a prospective cohort study of over 500 000 participants. We first developed a novel aging measure, MetaboAgeMort, using all‐cause mortality as a surrogate.^[^ ^19^ ^]^ Next, we evaluated its applicability by examining its associations with aging‐related outcomes (i.e., morbidity and mortality), and comparing its performance to conventional risk factors and several previously trained metabolomic clocks. Finally, we systematically identified the modifiable factors and genetic determinants for MetaboAgeMort (**Figure** **1**).

## 2 Results

### 2.1 Population Characteristics

As shown in Figure S1 (Supporting Information), 239 291 participants with complete data on plasma metabolomics and covariates at baseline were included. These participants had a median age of 58.3 years (interquartile range [IQR]: 50.6, 63.7), and the majority were female (53.0%), and white ethnicity (95.6%). To develop the MetaboAgeMort model, the 239 291 participants were randomly split into a training (n = 167 506) and a testing set (n = 71 785), with a 7 to 3 ratio. No significant differences were observed in the sociodemographic characteristics of participants between the training set and the testing set. Detailed characteristics of the total participants and by datasets are presented in Table S1 (Supporting Information).

### 2.2 Development of MetaboAgeMort and MetaboAgeMort Acceleration (MetaboAgeMortAccel)

During a median follow‐up of 13.9 years, we documented 20 447 deaths among 239 291 participants. After adjustment for potential confounders and accounting for multiple testing, a total of 185 metabolic biomarkers, encompassing amino acids, glycolysis‐related metabolites, ketone bodies, fatty acids, lipids, and lipoprotein subclasses, demonstrated significant correlations with all‐cause mortality (*P* < 0.05/249) (Table S2, Supporting Information).

To further select variables for inclusion in the MetaboAgeMort model, we applied a Cox regression model with least absolute shrinkage and selection operator (LASSO) penalization–where the hazard of all‐cause mortality was regressed on the 185 metabolic biomarkers and chronological age–in the training set. Finally, chronological age and 35 metabolic biomarkers, including average diameter for very low‐density lipoprotein (VLDL) particles, linoleic acid, ratio of omega‐3 fatty acids to total fatty acids, ratio of monounsaturated fatty acids to total fatty acids, ratio of linoleic acid to total fatty acids, alanine, histidine, leucine, valine, phenylalanine, tyrosine, glucose, pyruvate, citrate, 3‐hydroxybutyrate, acetate, acetoacetate, acetone, creatinine, albumin, glycoprotein acetyls, triglycerides in very large VLDL, free cholesterol in very large high‐density lipoprotein (HDL), total lipids in small HDL, cholesteryl esters in small HDL, triglycerides to total lipids ratio in large VLDL, phospholipids to total lipids ratio in very small VLDL, phospholipids to total lipids ratio in intermediate‐density lipoprotein (IDL), cholesteryl esters to total lipids ratio in IDL, triglycerides to total lipids ratio in large low‐density lipoprotein (LDL), triglycerides to total lipids ratio in medium LDL, phospholipids to total lipids ratio in small LDL, free cholesterol to total lipids ratio in small LDL, cholesteryl esters to total lipids ratio in very large HDL, free Cholesterol to total lipids ratio in small HDL, were selected (Table S3, Supporting Information).

**Figure 2:**
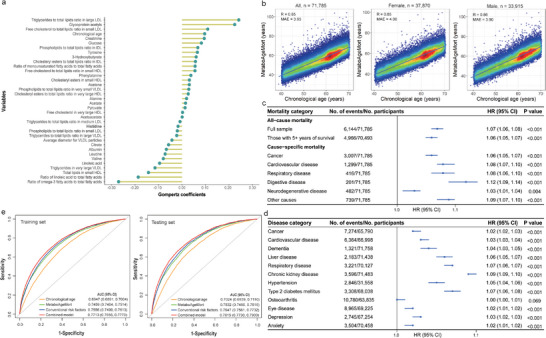
The performance and implications of MetaboAgeMort. a) The variable importance plot of MetaboAgeMort. The y‐axis lists the variable components of the MetaboAgeMort model, and the x‐axis indicates their corresponding Gompertz coefficients values. b) The distributions of MetaboAgeMort and chronological age across all participants and by sex in the testing set. Each scatter indicates a single participant. The correlation coefficient and MAE are shown in the left top part. c) Associations of MetaboAgeMort with all‐cause and cause‐specific mortality in the testing set. For all‐cause mortality, Cox model adjusted for chronological age, sex, ethnicity, education level, Townsend deprivation index, alcohol intake frequency, smoking status, regular exercise, healthy diet, body mass index, medication use, and prevalent diseases at baseline was used. We also repeated the analysis in those with ≥5 years of survival. For cause‐specific mortality, Fine and Gray's competing risk models adjusted for chronological age, sex, ethnicity, education level, Townsend deprivation index, alcohol intake frequency, smoking status, regular exercise, healthy diet, and body mass index were used. d) Associations of MetaboAgeMort with diseases in the testing set. Participants with a specific diagnosis before or at baseline were excluded prior to analysis. The same method for cause‐specific mortality was used. e) Receiver operating characteristic curves for 10‐year all‐cause mortality. The conventional risk factors included chronological age, sex, alcohol intake frequency, smoking status, body mass index, systolic blood pressure, triglycerides, creatinine, total cholesterol, high‐density lipoprotein cholesterol, and prevalent diabetes, cardiovascular disease, and cancer. The combined model included both MetaboAgeMort and conventional risk factors. MAE, mean absolute error; HR, hazard ratio; CI, confidence interval; AUC, area under the curve.

Next, MetaboAgeMort was developed using the methods previously proposed by Levine et al. in the training set.^[^ ^19^ ^]^ For more information about the MetaboAgeMort estimator, refer to **Figure** **2**a and Table S4 (Supporting Information). A profiling of the MetaboAgeMort performance was carried out in the testing set (n = 71 785). MetaboAgeMort ranged from 27.82 to 104.06 years, with a mean and median value of 55.81 (standard deviation [SD] = 9.18) and 56.10 (IQR: 48.99, 62.50) years. As shown in Figure 2b, MetaboAgeMort was highly correlated with chronological age across all participants (r = 0.85) and within each sex subgroup (female: r = 0.85; male: r = 0.86).

We also calculated a metric, MetaboAgeMortAccel, following the methods by Liu et al.^[^ ^20^ ^]^ MetaboAgeMortAccel represents the divergence of MetaboAgeMort from chronological age (i.e., whether a person appears younger [values < 0] or older [values > 0] than expected, based on his/her chronological age). The MetaboAgeMortAccel displayed a range of −16.43 to 41.29 years, with a mean and median value of 0 (SD = 4.78) and −0.41 (IQR: −3.29, 2.79) years in the testing set.

### 2.3 Association of MetaboAgeMort with Mortality

The associations of MetaboAgeMort with all‐cause and cause‐specific mortality in the testing set are demonstrated in Figure 2c. After adjustment for potential confounders, each additional year in MetaboAgeMort corresponded to a 7% rise in the risk of all‐cause mortality (Hazard Ratio [HR]: 1.07, 95% confidence interval [CI]: 1.06, 1.08) (Table S5, Supporting Information). Our finding remains consistent when: 1) stratified by chronological age, sex, ethnicity, education level, smoking status, alcohol intake frequency, regular exercise, healthy diet, and body mass index (BMI) category (Table S6, Supporting Information), 2) excluding participants who died within five years of follow‐up (Figure 2c; Table S5, Supporting Information), and 3) restricting the sample to diseases‐free participants (Table S7, Supporting Information). In addition, MetaboAgeMort was significantly positively associated with cause‐specific mortality, including cancer (HR: 1.06, 95% CI: 1.05, 1.07), cardiovascular disease (CVD, HR: 1.08, 95% CI: 1.07, 1.10), respiratory disease (HR: 1.08, 95% CI: 1.06, 1.10), digestive disease (HR: 1.12, 95% CI: 1.09, 1.14), neurodegenerative disease (HR: 1.03, 95% CI: 1.01, 1.04), and other causes (HR: 1.09, 95% CI: 1.07, 1.10) (Figure 2c; Table S5, Supporting Information).

**Figure 3:**
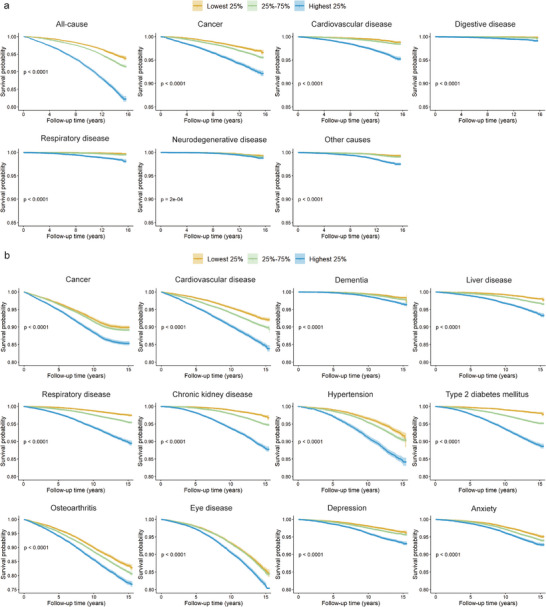
a) Kaplan‐Meier survival plots of all‐cause mortality, cause‐specific mortality and b) 12 aging‐related diseases according to quartiles of the MetaboAgeMort Acceleration. Blue indicates the top quartile, green indicates the second and third quartile, and yellow indicates the bottom quartile with 95% confidence intervals in the testing set. The y‐axis indicates the survival rate, and the x‐axis indicates follow‐up time (in years).

The Kaplan‐Meier survival curves indicated that individuals in the highest quartile group (Q4) of MetaboAgeMortAccel had significantly increased risks of all‐cause and cause‐specific mortality when compared to those in the lowest quartile (Q1) (**Figure** **3**a; Figure S3a and Table S8, Supporting Information). The all‐cause mortality rates of the highest quartile group (Q4) were found to be comparable, or in certain instances higher, than those of the lowest quartile group (Q1), despite the latter being 10 years older chronologically (Figure S4, Supporting Information).

### 2.4 Associations of MetaboAgeMort with Disease Incidences

The associations of MetaboAgeMort with the risk of multiple diseases in the testing set are depicted in Figure 2d. After adjustment for potential confounders, each 1‐year increment in MetaboAgeMort was significantly associated with higher risks of cancer (HR: 1.02, 95% CI: 1.02, 1.03), CVD (HR: 1.03, 95% CI: 1.03, 1.04), dementia (HR: 1.04, 95% CI: 1.03, 1.05), liver disease (HR: 1.06, 95% CI: 1.05, 1.07), respiratory disease (HR: 1.07, 95% CI: 1.06, 1.07), chronic kidney disease (HR: 1.09, 95% CI: 1.09, 1.10), hypertension (HR: 1.05, 95% CI: 1.04, 1.06), type 2 diabetes mellitus (T2DM, HR: 1.07, 95% CI: 1.06, 1.08), eyes disease (HR: 1.02, 95% CI: 1.01, 1.02), depression (HR: 1.03, 95% CI: 1.02, 1.03), and anxiety (HR: 1.02, 95% CI: 1.01, 1.02), with an exception of osteoarthritis (HR: 1.00, 95% CI: 1.00, 1.01) (Table S9, Supporting Information).

The Kaplan‐Meier survival curves exhibited discernible trajectories among the quartile groups of MetaboAgeMortAccel (Figure 3b; Figure S3b, Supporting Information). The group in the highest quartile (Q4) showed a significant association with increased risks of multiple disease incidences compared to the lowest quartile (Q1) (Tables S10 and S11, Supporting Information).

### 2.5 Discriminative Improvements Beyond Clinical Predictors

Based on the findings depicted in Figure 2e, it is evident that the area under the curve (AUC) of MetaboAgeMort displayed a considerable improvement relative to chronological age, aligning it more closely with the AUC of the conventional risk factors model. MetaboAgeMort added predictive utility of 10‐year mortality beyond conventional risk factors (i.e., chronological age, sex, alcohol intake frequency, smoking status, BMI, systolic blood pressure, triglycerides, creatinine, total cholesterol, HDL cholesterol, and prevalent diabetes, CVD and cancer).^[^ ^12^ ^]^ Compared with the conventional risk factors model, the combined model including MetaboAgeMort had better discrimination ability, as demonstrated by significantly increased C‐statistics (0.017, *P* < 0.001) (Table S12, Supporting Information). The superior performance of MetaboAgeMort was further confirmed through substantial enhancements in reclassification, as evaluated by integrated discrimination improvement (IDI: 0.018, 95% CI: 0.004, 0.021) (Table S12, Supporting Information), suggesting that MetaboAgeMort captures something above and beyond what can be explained for mortality risk by conventional risk factors.

### 2.6 The comparison of MetaboAgeMort with MetaboAge and MetaboHealth Score

In addition, we calculated two pre‐existing well‐known multi‐metabolite scores (i.e., MetaboAge^[^ ^6^ ^]^ and MetaboHealth score^[^ ^13^ ^]^), and assessed their associations with the mortality risk and multiple disease incidences. The MetaboAge score predicts chronological age (in years) directly. Two versions of MetaboAge were calculated: MetaboAge_LM (generated through linear regression), and MetaboAge_EN (generated through ElasticNET regression).^[^ ^21^ ^]^ The MetaboHealth score is a multivariate model predicting all‐cause mortality. It was calculated as the weighted sum of 14 log‐transformed and cohort‐scaled metabolites. The distributions of these multi‐metabolite scores in the testing set are shown in Figure S5a (Supporting Information). As shown in Table S13 (Supporting Information), after adjustment for potential confounders, the highest quartile group (Q4) of the MetaboHealth score has significantly increased risks of all‐cause mortality, cause‐specific mortality, and multiple incident diseases, compared to the lowest quartile group (Q1). The MetaboAge_EN showed significant association with all‐cause mortality, CVD mortality, and several cardiometabolic diseases (i.e., CVD and T2DM). Conversely, MetaboAge_LM did not demonstrate any appreciable relationship with these same clinical endpoints. As shown in Table S14 (Supporting Information), even after mutual adjustment, both MetaboAgeMort and MetaboHealth score maintained significant associations with all‐cause mortality. Notably, the Akaike Information Criterion (AIC) for the model integrating MetaboAgeMort indicated a relatively superior fit, implying that MetaboAgeMort may have a higher predictive capacity or enhanced explanatory power in forecasting all‐cause mortality when compared to the model integrating the MetaboHealth score.

Moreover, we compared MetaboAgeMort with these multi‐metabolite scores, in terms of their predictive utility for 10‐year mortality risk and multiple disease incidences. As shown in Figure S5b (Supporting Information), MetaboAgeMort displayed a significantly higher AUC than that of the MetaboAge and MetaboHealth score for 10‐year all‐cause mortality prediction (*P* < 0.001). Combining MetaboAgeMort with conventional risk factors resulted in an AUC that outperformed models integrating MetaboAge (MetaboAge_EN and MetaboAge_LM, both *P* < 0.001) or MetaboHealth score (*P* = 0.021) with the same conventional risk factors. When combining MetaboAgeMort with MetaboHealth score and conventional risk factors, the resulting AUC was higher than that of the model integrating only MetaboHealth score with conventional risk factors, but this difference was not significant when compared to the model integrating only MetaboAgeMort with conventional risk factors. This finding suggests that MetaboAgeMort may capture something beyond what can be explained for mortality risk by MetaboHealth score, when taking into account conventional risk factors. MetaboAgeMort also exhibited a higher predictive utility for multiple diseases (except for cancer incidence) compared to the MetaboHealth score (Figure S5c, Supporting Information). After accounting for chronological age and sex, the AUC of the model integrating both MetaboAgeMort and MetaboHealth score was higher than that of the model with MetaboHealth score alone, but did not show a significant improvement over the model with MetaboAgeMort alone. This finding suggests that MetaboAgeMort may also capture something beyond what can be explained for disease incidences by MetaboHealth score.

### 2.7 Modifiable Factors for MetaboAgeMort

**Figure 4:**
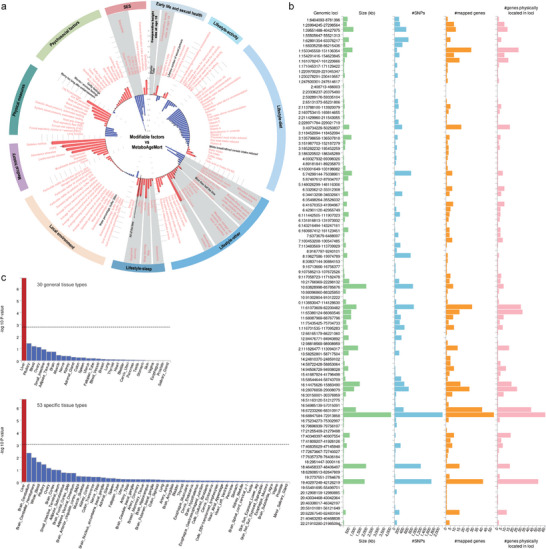
The modifiable factors and genetic determinants for MetaboAgeMort. a) The circular barplot shows the associations between MetaboAgeMort and 107 modifiable factors (positive association in red bars and negative associations in blue bars). The linear models were adjusted for chronological age, sex, and ethnicity. Bonferroni's significant traits (*P* < 0.05/107) are in pink text and the others (*P* > 0.05/107) are in black text. b) Summary of 99 genomic risk loci based on genome‐wide association analysis of MetaboAgeMort. c) Gene‐tissue expression results (*P* values significant at the Bonferroni‐corrected level 0.05/30 for 30 general tissue types or 0.05/53 for 53 specific tissue types in red bars and others in blue bars. SES, socioeconomic status; COPD, chronic obstructive pulmonary disease; SNPs, single nucleotide polymorphisms.

Then we investigated the modifiable factors of MetaboAgeMort. We considered a total of 107 potentially modifiable factors from the UKB baseline survey. After Bonferroni correction, 99 factors showed a significant association with MetaboAgeMort (*P* < 4.67 × 10^−4^, **Figure** **4**a; Table S15, Supporting Information). For 16 factors across four categories, the associations were relatively more substantial (≥ 2 years of change in MetaboAgeMort) per 1‐SD change in the factor or group contrast (Figure S6, Supporting Information), such as forced vital capacity (FVC) (tertile 3 versus tertile 1, β =  −2.22, 95% CI:  −2.28, −2.15), body fat percentage (tertile 3 versus tertile 1, β =  3.55, 95% CI: 3.49, 3.61), dietary index (tertile 3 versus tertile 1, β =   −2.04, 95% CI: −2.09, −1.98), and average total household income before tax (greater than 100,000 versus less than 18000, β =   −3.34, 95% CI: −3.44, −3.24). Comparable findings were observed when additionally adjusting for BMI, or when stratifying by chronological age and sex (Table S15).

### 2.8 Genetic determinants for MetaboAgeMort

To better understand the genetic mechanisms underlying metabolomic aging, we performed a genome‐wide association study (GWAS) analysis of MetaboAgeMort. In the GWAS analysis, 11 688 SNPs significantly associated with MetaboAgeMort were identified (*P* < 5 × 10^−8^) (Figure S7a, Supporting Information). The SNP‐derived heritability of MetaboAgeMort was 38.26% (*P* = 2.23 × 10^−86^). Using the Functional Mapping and Annotation (FUMA) online platform (v1.5.2), we pinpointed 1068 independent significant SNPs, 319 lead SNPs, and 99 genomic risk loci (Tables S16–S19, Supporting Information). Furthermore, 585 prioritized genes that may be involved in the genetic etiology of MetaboAgeMort were identified by positional mapping (Table S20, Supporting Information). The leading SNP of the most significant locus (rs174575, locus 57) was positioned near or within *FADS1* and *FADS2* on chromosome 11. The leading SNP of the second most significant locus (rs217184, locus 78) was in *TXNL4B*, *HPR*, and *HP* on chromosome 16 (Table S21, Supporting Information). Summary results per genomic risk locus are shown in Figure 4b.

We also performed analyses pertaining to genes and tissue enrichment using Multi‐Marker Analysis of GenoMic Annotation (MAGMA) v1.08 within FUMA. In genome‐wide gene‐based association analysis (GWGAS), 310 genes were determined to be genome‐wide significant after applying Bonferroni correction (Table S22, Supporting Information), where 271 genes were also positionally mapped to significant loci from the SNP‐based analysis above. The gene‐set analysis identified 13 significant gene sets after Bonferroni correction, including lipid‐related biological process (e.g., reverse cholesterol transport, cholesterol metabolism, and phospholipid homeostasis), CYP2E1 reactions, and liver specific genes (Table S23, Supporting Information). The tissue‐enrichment analysis indicated that liver displayed significant specificity in gene expression for the MetaboAgeMort‐associated genes (Figure 4c; Tables S24 and S25, Supporting Information).

Given the potential similarity between the MetaboHealth score and MetaboAgeMort, we also performed GWAS and further FUMA analyses for the MetaboHealth score (Figure S7b and Tables S26–S33, Supporting Information). As shown in Figure S9a (Supporting Information), a total of 215 independent SNPs, 44 lead SNPs, 16 genomic risk loci, and 244 protein‐coding gene overlaps were detected across the two scores. The gene‐set analyses of MetaboHealth score identified 18 significant gene sets, of which 6 were also identified for MetaboAgeMort. Moreover, the tissue‐enrichment analysis of MetaboHealth score also showed significant enrichment in liver (Figure S9b, Supporting Information). These findings suggest that MetaboAgeMort and MetaboHealth capture distinct yet overlapping aspects of metabolic aging and health, partly due to the differing sets of metabolic markers included in the two models.

## 3 Discussion

Leveraging the large‐scale metabolomics data, we have formulated a groundbreaking aging measure, MetaboAgeMort, based on 10‐year all‐cause mortality risk prediction. The MetaboAgeMort demonstrated remarkable predictive utility of mortality risk across a wide range of demographic and socioeconomic stratifications, as well as health behavior factors and causes of death. Significantly, the conventional risk factors were augmented by MetaboAgeMort in terms of predictive accuracy for 10‐year mortality. Meanwhile, our study has revealed compelling associations between accelerated metabolomic aging within the same age group and an increased likelihood of various health‐related outcomes. This implies that it could serve as a comprehensive indicator for health and mortality risk stratification in a clinical setting. Next, we identified 99 modifiable factors across seven categories for MetaboAgeMort, highlighting the crucial role played by body composition, healthy diet, SES, and pulmonary function in the process of metabolomic aging. The genetic analyses ultimately revealed 99 significant genomic risk loci and 271 genes linked to MetaboAgeMort, thus offering new insights into the genetic architecture of metabolomic aging.

Previous studies have generated metabolomic clocks while using chronological age as a surrogate, such as MetaboAge^[^ ^6^ ^]^ and the model by Ala‐Korpela et al.^[^ ^22^ ^]^ In comparison, our MetaboAgeMort further incorporates information on all‐cause mortality risk, which is considered a more reliable surrogate for biological aging than chronological age, through a sophisticated modeling method.​ As one would expect from a measure of aging, MetaboAgeMort not only assesses the risks of all‐cause and cause‐specific mortality, but also the risks of various incident diseases, highlighting its considerable potential in the early detection of individuals at risk and facilitating timely and effective interventions. It is worth mentioning that we have substantiated the contribution of MetaboAgeMort in enhancing the predictive capacity for 10‐year all‐cause mortality risk in addition to conventional risk factors. These findings translate into potential clinical application of MetaboAgeMort as an additional source of discriminatory information to refine comprehensive risk assessments for death and diseases. To date, many studies have endeavored to identify metabolite predictors of mortality risk and have successfully developed multivariate metabolite scores (e.g., MetaboHealth score^[^ ^13^ ^]^ and the model by Lau et al.^[^ ^23^ ^]^). These scores exhibited significant association with all‐cause mortality and a few diseases (e.g., CVD, cancer, and T2DM).^[^ ^24^, ^25^ ^]^ Some of these multi‐metabolite scores showed moderate associations with chronological age even in the training set. Next, the utilization of these multi‐metabolite scores is constrained due to the reliance on cohort‐specific scaled biomarker values, thereby impeding its clinical application in identifying individuals with a propensity for accelerated aging and comparison across different populations. MetaboAgeMort, directly calculated from individual‐level data, more intuitively shows the heterogeneity in mortality risk among individuals of the same chronological age, exhibiting enhanced generalization.

The 35 metabolic biomarkers employed in our study to develop MetaboAgeMort are implicated in diverse processes, including lipoprotein and fatty acid metabolism, fluid balance, and inflammation, indicating that aging is an intricate multidimensional phenomenon. Previous studies have sought to elucidate the interaction between metabolites and aging. In line with the discoveries made by Deelen et al.,^[^ ^13^ ^]^ our study revealed that average diameter for VLDL particles, histidine, leucine, phenylalanine, valine, glucose, acetoacetate, albumin, glycoprotein acetyls, the ratio of polyunsaturated fatty acids to total fatty acids, and total lipids in small HDL were essential independent indicators for mortality. Among these, the ratios of polyunsaturated fatty acids to total fatty acids—specifically, the ratio of omega‐3 fatty acids to total fatty acids and the ratio of linoleic acid to total fatty acids—hold considerable importance in our model. Polyunsaturated fatty acids have been shown to play beneficial roles in combating oxidative stress, reducing inflammation, enhancing insulin sensitivity, and preserving mitochondrial function,^[^ ^26^ ^]^ all of which contribute positively to slowing down the process of aging. Glycoprotein acetyls, another key component, serves as a marker of inflammation and have been established as a significant factor related to multiple age‐associated diseases and the acceleration of the aging process.^[^ ^27^ ^]^ In addition, we observed associations for several other metabolic biomarkers, such as ketone bodies (KBs) and relative lipoprotein lipid concentrations. KBs are endogenous fuels generated by the liver in response to metabolic stress.^[^ ^28^ ^]^ In a healthy community‐based population, higher elevated endogenous KBs have shown to be positively associated with all‐cause mortality.^[^ ^29^ ^]^ Relative lipoprotein lipid concentrations play a role in lipid homeostasis and their associations with mortality may be partially attributed to their regulatory effect on plasma triglyceride levels, a critical mortality risk factor.^[^ ^30^ ^]^ Collectively, our study contributes to the comprehension of metabolic alterations that underlie the process of aging.

The primary focus of aging research has been on the development of strategies to combat aging. Measures like MetaboAgeMort, which capture future morbidity and mortality risk, could facilitate evaluation of intervention efficacy while eliminating the requirement for extended follow‐up periods. A recent study in the UK Airwave cohort has discovered correlations between metabolomic aging and several factors such as overweight, obesity, heavy drinking, diabetes, depressive symptoms, depression, anxiety, and post‐traumatic stress disorder.^[^ ^8^ ^]^ Similarly, Lau et al. reported similar findings regarding obesity, diabetes, smoking, and physical inactivity.^[^ ^23^ ^]^ Using a larger‐scale population‐based cohort, our study meticulously investigated the modifiable factors associated with metabolomic aging. Consequently, we identified a total of 99 potential factors across seven distinct categories: SES, early life and sexual health, medical history, physical measures, psychosocial factors, local environment, and lifestyle. Stronger associations (β > 2) were quantitatively observed for 16 factors related to pulmonary function, body composition, SES, dietary quality, smoking status, alcohol intake, and disease status, all of which have previously been reported to be associated with aging.^[^ ^31^, ^32^, ^33^, ^34^ ^]^ Our study provides a metabolomic insight into the mechanisms linking these factors to aging process. Certain local environmental factors, such as fine particulate matter (PM_2.5_) and nitrogen dioxide (NO_2_), have been evidenced to potentiate oxidative stress responses within biological systems, impair mitochondrial function, and subsequently lead to metabolic irregularities.^[^ ^35^, ^36^ ^]^ The correlation between these factors and MetaboAgeMort underscores the significance of enacting urban planning and environmental preservation policies to slow down the aging process. Furthermore, MetaboAgeMort demonstrated responsiveness to specific early‐life exposure factors, suggesting that targeted interventions during the critical developmental period, such as optimizing maternal nutrition and improving the developmental environment for children, could potentially contribute to the promotion of healthy aging and reduction of risks associated with late‐life mortality and morbidity.^[^ ^37^ ^]^

Genetics play a substantial role in determining individual biological aging rates.^[^ ^38^ ^]^ Based on our current understanding, this study offers the initial evidence regarding the genetic determinants of metabolomic aging. By utilizing genotyping data, we pinpointed 99 genomic risk loci and 271 genes associated with MetaboAgeMort. The most significant SNPs were identified within the *FADS* cluster (*FADS1*, *FADS2*) on chromosome 11. These genes have emerged as significant genes in prior studies on serum omega‐3 fatty acid,^[^ ^39^ ^]^ a crucial type of polyunsaturated fatty acids that have demonstrated favorable impacts on age‐related diseases (e.g., CVD and metabolic diseases).^[^ ^40^ ^]^ We also identified several genome‐wide significant genes on chromosome 16, such as *TXNL4B*, *HPR*, and *HP*. These genes play integral roles in modulating the core biological mechanisms, including cell cycle progression, oxidative balance maintenance, protein conformational dynamics and stability, which all exhibit profound interdependencies with the aging process.^[^ ^41^, ^42^ ^]^ Moreover, the MAGMA gene‐set analysis unveiled the critical involvement of lipid metabolism, CYP2E1‐mediated reactions, and liver‐specific genes in metabolomic aging, providing valuable insights into the underlying molecular mechanisms and potential therapeutic targets pertinent to this process. The tissue‐enrichment analysis further emphasizes the importance of liver in metabolomic aging. This finding implies that strategies focused on preserving or restoring liver health, such as modulating key metabolic pathways, enhancing antioxidant defenses, and stem cell therapy, may have far‐reaching systemic benefits in countering metabolomic aging process. Further research is needed to refine these potential strategies and evaluate their efficacy in promoting healthy aging and preventing age‐related diseases. Notably, the pathways identified for MetaboAgeMort were distinct from those enriched by genes associated with PhenoAgeAccel or BioAgeAccel,^[^ ^17^ ^]^ thus reaffirming that the heterogeneous aging patterns observed among individuals might be partly attributed to varying genetic susceptibilities.

Some limitations in this study should also be noted. First, the number of biomarkers captured by the targeted NMR platform is only a fraction of the metabolites in the human plasma. Nevertheless, NMR has the ability to offer highly accurate quantification at a minimal expense, thus facilitating the straightforward implementation of metabolomic clocks in population health. Second, the association between modifiable risk factors and MetaboAgeMort is cross‐sectional, and further causal inferences are needed. Third, even though we have taken into account numerous modifiable risk factors, it is conceivable that certain factors may have been unintentionally neglected. Moreover, the potential correlation among the modifiable factors may impact the interpretation of the independent effects of each factor on metabolomic aging. Fourth, the majority of participants in the UKB were White British and tended to be healthier and wealthier,^[^ ^43^ ^]^ and thus, our sample may be less representative of the overall UK adult population. Fifth, the reliability of the concentration measurements in the Nightingale NMR metabolomics data might be affected by sample and data processing, and analytical batch effects.^[^ ^21^, ^44^ ^]^ Moreover, known sample dilution issues affecting the UKB data suggest that it may be more appropriate to treat this specific dataset as quantitative in relative terms only. Thus, our metabolomic model may not be directly transferable across different versions of the data. When applying the model to other datasets, we recommend either retraining the score using the target dataset or conducting an extensive re‐evaluation of the model and its associations with relevant endpoints to ensure validity and accuracy.

In conclusion, we have successfully developed a novel metabolomic‐based measure of aging known as MetaboAgeMort. This measure showed strong predictive power of mortality and various diseases in the testing set. Of particular significance is the fact that MetaboAgeMort can enhance the predictive accuracy of 10‐year mortality beyond conventional risk factors. These findings imply that MetaboAgeMort has remarkable potential as a comprehensive measure of overall health and risk of mortality; nevertheless, further external validations in independent cohorts are needed. The potential of body composition, healthy diet, SES, pulmonary function, smoking status, and alcohol intake were highlighted as possible factors for delaying metabolomic aging. Our research has led us to the identification of 99 significant genomic risk loci and 271 genes linked to MetaboAgeMort. This breakthrough sheds new light on the complex genetic underpinnings governing the metabolomic aging processes.

## 4 Experimental Section

### Study Participants

The UKB was a large prospective cohort study that comprised over 500 000 participants aged 37–73 years at the time of baseline assessment (2006‐2010). Information was collected via touch‐screen questionnaires, biological samples, physical measurements, and linked medical or death register records. Detailed study design and methodology were described elsewhere.^[^ ^45^ ^]^ Ethics for the UKB was approved by the North West Multicenter Research Ethics Committee, and all participants had provided signed informed consent.

### Plasma metabolomics

A total of 251 metabolic biomarkers for EDTA plasma samples from a randomly selected subset of approximately 280 000 UKB participants were measured between June 2019 and April 2020 (Phase 1) and April 2020 and June 2022 (Phase 2) using a high‐throughput NMR metabolomics platform developed by Nightingale Health Ltd. The metabolic biomarkers span multiple metabolic pathways, including fatty acids, fatty acid compositions, and lipoprotein lipids in 14 subclasses, as well as various low‐molecular weight metabolites, such as ketone bodies, amino acids, and glycolysis metabolites. Detailed protocols for sample collection and methodology for the Nightingale NMR pipeline were described elsewhere.^[^ ^46^, ^47^ ^]^ The present study considered 249 available metabolic biomarkers (except forglucose‐lactate and spectrometer‐corrected alanine) and the values of each metabolic biomarker were transformed using natural logarithmic transformation (ln[x+1]) followed by Z‐normalisation prior to analysis.

### Development of MetaboAgeMort and MetaboAgeMortAccel

Two sequential steps were undertaken in order to create the MetaboAgeMort model. In the first step, aging‐related metabolic biomarkers were identified. Initially, the association of each metabolic biomarker (per 1‐SD increment) with all‐cause mortality was evaluated using multivariable Cox regression models, with adjustment for chronological age, sex, ethnicity, education level, Townsend deprivation index (TDI), alcohol intake frequency, smoking status, regular exercise, healthy diet, BMI, cholesterol‐lowering medication, anti‐hypertensive medication, anti‐diabetes medication, and prevalent diseases at baseline (i.e., cancer, CVD, hypertension, diabetes mellitus, and chronic obstructive pulmonary disease [COPD]) among all participants (n = 239 291), and 185 metabolic biomarkers were selected with Bonferroni correction. Then, a Cox regression model was employed with LASSO penalization–where the hazard of all‐cause mortality was regressed on 185 metabolic biomarkers and chronological age–in the training set. Finally, an optimal λ of 0.00097 was selected via tenfold cross‐validation, and 36 variables, including chronological age were assigned nonzero coefficients.

In the second step, the MetaboAgeMort was constructed in the training set by adopting the methodology previously proposed by Levine et al.^[^ ^19^ ^]^ Two proportional hazards regression models based on the parametric Gompertz distribution were fitted: one used 36 variables selected above as predictors, and the other used only chronological age as a predictor. Based on the two models, the 10‐year all‐cause mortality risk was predicted using the cumulative distribution function, respectively. The mortality risk was then converted into units of years by equating the risk from the two models and solving for age, thus obtaining MetaboAgeMort. ​In general, an individual's MetaboAgeMort represents the chronological age within the general population corresponding to that individual's mortality risk. ​For example, two individuals were chronologically 40 years old, but one may had a MetaboAgeMort of 45 years and the other a MetaboAgeMort of 35 years, indicating that they had the average mortality risk of someone who was chronologically 45 or 35 years old, respectively. In addition, a metric was calculated, MetaboAgeMortAccel, following the methods by Liu et al.^[^ ^20^ ^]^ MetaboAgeMortAccel represents the divergence of MetaboAgeMort from chronological age, defined as the residual resulting from a least‐squares linear model when regressing MetaboAgeMort on chronological age.

### Health‐Related Outcomes

Information on date and cause of death was obtained through the linkage to national death registries. The all‐cause and cause‐specific mortality (i.e., cancer, CVD, respiratory disease, neurodegenerative disease, digestive disease, and other causes) were determined using the International Classification of Disease (ICD)−10 codes (Table S34, Supporting Information).^[^ ^48^ ^]^ Follow‐up time was calculated from the date of baseline assessment to the date of death, loss to follow‐up, or end of follow‐up (Dec 31, 2022), whichever came first.

Information on diagnoses and medical conditions of the participants were obtained through the linked hospital inpatient record data, self‐reported data, and primary care data from the UK National Health Services. The incident diseases were ascertained by the ICD‐9 and ICD‐10 codes (Table S35, Supporting Information). Follow‐up time was calculated from the date of baseline assessment to the date of first diagnosis of the disease, death, loss to follow‐up, or end of follow‐up (Oct 31, 2022), whichever came first.

### MetaboAge and MetaboHealth Score

The MetaboAge score predicts chronological age (in years) directly.^[^ ^6^ ^]^ Two different models were utilized: a linear regression model (MetaboAge_LM) and an ElasticNET regression model (MetaboAge_EN). The model weights/coefficients were obtained from the most recent publication by Bizzarri et al.^[^ ^21^ ^]^ In quality control, three samples were excluded with one or more zero values per sample, as well as one or more concentrations that were more than five times the SD away from the overall mean of the feature. The MetaboHealth score was a multivariate model predicting all‐cause mortality.^[^ ^13^ ^]^ It was calculated as the weighted sum of 14 log‐transformed and cohort‐scaled metabolites, using the R‐package MiMIR.^[^ ^49^ ^]^ To avoid infinite values after log‐transformation, a value of 1 was added to the metabolites containing any zero.

### Modifiable Factors

A total of 107 potentially modifiable factors have been taken into account from the UKB baseline survey. Details of processing the factors are presented in Table S36 (Supporting Information). These factors were classified into seven categories: local environment (e.g., greenspace percentage, buffer 1000m), psychosocial (e.g., nervous feelings), SES (e.g., TDI), medical history (e.g., prevalent CVD at baseline), early life and sexual health (e.g., breastfed as a baby), physical measures (e.g., handgrip strength), and lifestyle (e.g., healthy diet).

### Genome‐Wide Association Analysis

To better understand the genetic mechanisms underlying MetaboAgeMort, a GWAS analysis was performed using the data from UKB v3 genotyping release. The SNPs were excluded if meeting any of the following criteria: 1) minor allele frequency < 0.01, 2) Hardy‐Weinberg equilibrium test *P* < 1.0 × 10^−6^, 3) missing minor allele frequency, or results of Hardy‐Weinberg equilibrium test. This particular aspect of the analysis was constrained to individuals of White British descent (n = 226 937). The fastGWA‐MLM analysis in the Genome‐wide complex trait analysis (GCTA) software (version 1.94.1) was used to perform GWAS analysis.^[^ ^50^ ^]^ Models included chronological age, sex, genotype array, and the top 10 principal components as the covariates. The genome‐based restricted maximum likelihood (GREML) method in GCTA was used to estimate the SNP‐based heritability (variance explained by all the SNPs).^[^ ^51^ ^]^

### Functional Mapping and Annotation

The FUMA online platform (v1.5.2) was applied for functional mapping and annotation of GWAS results (default parameters were used unless explicitly stated otherwise), with annotations derived from the human genome assembly GRCh37 (hg19).^[^ ^52^ ^]^ To identify independent genomic risk loci (defined by r^2^ > 0.6) and variants in linkage disequilibrium (LD) with lead SNPs, the SNP2Gene module was applied using the genetic data of European populations in 1000G phase3 as the reference.^[^ ^53^ ^]^ Positional mapping with a 10 kilobase (kb) window size was employed to map risk loci to neighboring protein‐coding genes.

### Gene‐Based Association, Gene‐Set, and Gene‐Property Analyses with MAGMA

Pertaining to genes and tissue enrichment (i.e., GWGAS, gene‐set, and tissue expression analyses) analyses were performed using MAGMA v1.08 within FUMA.^[^ ^54^ ^]^ For each of the 18 955 protein‐coding genes, GWGAS accessed the joint effect of all variants within the gene. Bonferroni correction was used to establish the genome‐wide significance threshold (*P* < 0.05/18 955 = 2.64 × 10^−6^). Gene‐set analysis was further performed using hypergeometric tests for curated gene sets and Gene Ontology (GO) terms from MsigDB v7.0 to discern whether specific biological pathways or cellular functions were implicated in the genetic etiology of MetaboAgeMort, with Bonferroni correction also being utilized. Simultaneously, tissue enrichment analysis was carried out with 30 general and 53 specific tissue types from GTEx v8.^[^ ^55^ ^]^

### Covariates

Information on chronological age, sex (female or male), ethnicity (White or Non‐white), education level (high, intermediate, or low), alcohol intake frequency (never or special occasions only, one to three times per month, one to four times per week, or daily or almost daily), smoking status (never, previous, or current smoker), regular exercise (yes or no), healthy diet (yes or no), and medication information were collected through questionnaire interview. The TDI was assigned by participants’ postcodes, representing SES levels.^[^ ^56^ ^]^ BMI (kg/m^2^) was calculated as measured weight/height^2^.

### Statistical Analysis

Baseline characteristics were described using median (interquartile range, IQR) or count (percentage). Two‐sample Wilcoxon test for continuous variables and chi‐squared test for categorical variables were used to test the differences between the training and the testing set.

The study's roadmap is illustrated in Figure 1. The Cox proportional hazard model was used to evaluate the association between MetaboAgeMort and all‐cause mortality, with adjustment for chronological age, sex, ethnicity, education level, TDI, alcohol intake frequency, smoking status, regular exercise, healthy diet, BMI, medication use, and prevalent diseases at baseline. To further assess robustness, the analyses were repeated by 1) stratified by several demographic, socioeconomic, as well as health behavior factors, 2) excluding participants who died within 5 years of follow‐up to reduce the influence of end‐of‐life metabolomic status, and 3) only including participants who were free of prevalent diseases (i.e., cancer, CVD, hypertension, diabetes mellitus, COPD, and depression) at baseline to minimize the influence of reverse causality. Proportional hazards of the associations were tested using Schoenfeld's residuals. The participants were then grouped into quartiles of MetaboAgeMortAccel. The Kaplan‐Meier plots were drawn to visualize survival curves.

The evaluation of MetaboAgeMort associations with cause‐specific mortality and incident diseases was conducted using Fine and Gray's competing risk models.^[^ ^57^ ^]^ When constructing models evaluating the associations of MetaboAgeMort with incident diseases, the participants with a specific diagnosis before or at the time of recruitment were excluded from the models. Chronological age, sex, ethnicity, education level, TDI, alcohol intake frequency, smoking status, regular exercise, healthy diet, and BMI were used as covariates. The associations of MetaboAgeMortAccel with mortality and incident diseases were evaluated using the same method as that for MetaboAgeMort. The second and the third MetaboAgeMortAccel quartiles (Q2 and Q3) were set as the reference.

Next, receiver operating characteristic (ROC) curves were used to evaluate the utility of MetaboAgeMort for 10‐year all‐cause mortality risk prediction beyond conventional risk factors. C‐statistic and IDI were calculated, in comparison to that of the conventional risk factors model. In addition, ROC was used to compare its predictive utility for 10‐year all‐cause mortality risk and multiple disease incidences with several previously trained multi‐metabolite scores (i.e., MetaboAge and MetaboHealth score). Moverover, the associations of these multi‐metabolite scores with cause‐specific mortality and diverse age‐related diseases were tested using the same methods employed for MetaboAgeMort.

Multivariable linear regression models were applied to test the response of MetaboAgeMort (response variable) for each modifiable factor (independent variable), with a Bonferroni‐corrected significance threshold for identifying top hits (*P* < 0.05/107 = 4.67 × 10^−4^). In these analyses, continuous variables were Z‐normalised, and results were shown as β coefficients per 1‐SD increment in the corresponding factor (based on the availability of each individual factor). Moreover, continuous modifiable factors were also divided into tertiles and the results using the lowest tertile as a reference were showed. The models were adjusted for chronological age, sex, and ethnicity. Stratified analyses according to chronological age (< 60 and ≥ 60 years) and sex (male and female) were conducted, utilizing Bonferroni‐correction method to determine top hits.

Data analyses and visualization were all performed in R version 4.2.2. A two‐sided *P* of ≤ 0.05 was considered statistically significant.

### Ethics Approval and Consent to Participate

UK Biobank has approval from the North West Multi‐Centre Research Ethics Committee as a Research Tissue Bank approval in 2011 and is renewed every 5 years, which allows researchers to use data from UK Biobank without an additional ethical clearance. All participants have provided signed informed consent.

## Conflict of Interest

The authors declare no conflict of interest.

## Supporting information



Supporting Information

Supporting Information

## Data Availability

The data that support the findings of this study are openly available in UK Biobank at [https://www.ukbiobank.ac.uk/], reference number [61856].
