# Supplementary material for: A Novel Metabolomic Aging Clock Predicting Health Outcomes and Its Genetic and Modifiable Factors
Source: Adv Sci (Weinh). 2024 Sep 27;11(43):2406670. doi: 10.1002/advs.202406670 (PMC11578329; doi:10.1002/advs.202406670)
Supplement: Supplementary file 1 — Supporting Information [file ADVS-11-2406670-s001.docx]

Supporting Information

A Novel Metabolomic Aging Clock Predicting Health Outcomes and Its Genetic and Modifiable Factors

*Xueqing Jia, Jiayao Fan, Xucheng Wu, Xingqi Cao, Lina Ma, Zeinab Abdelrahman, Fei Zhao, Haitao Zhu, Daniele Bizzarri, Erik B van den Akker, P. Eline Slagboom, Joris Deelen, Dan Zhou, Zuyun Liu**


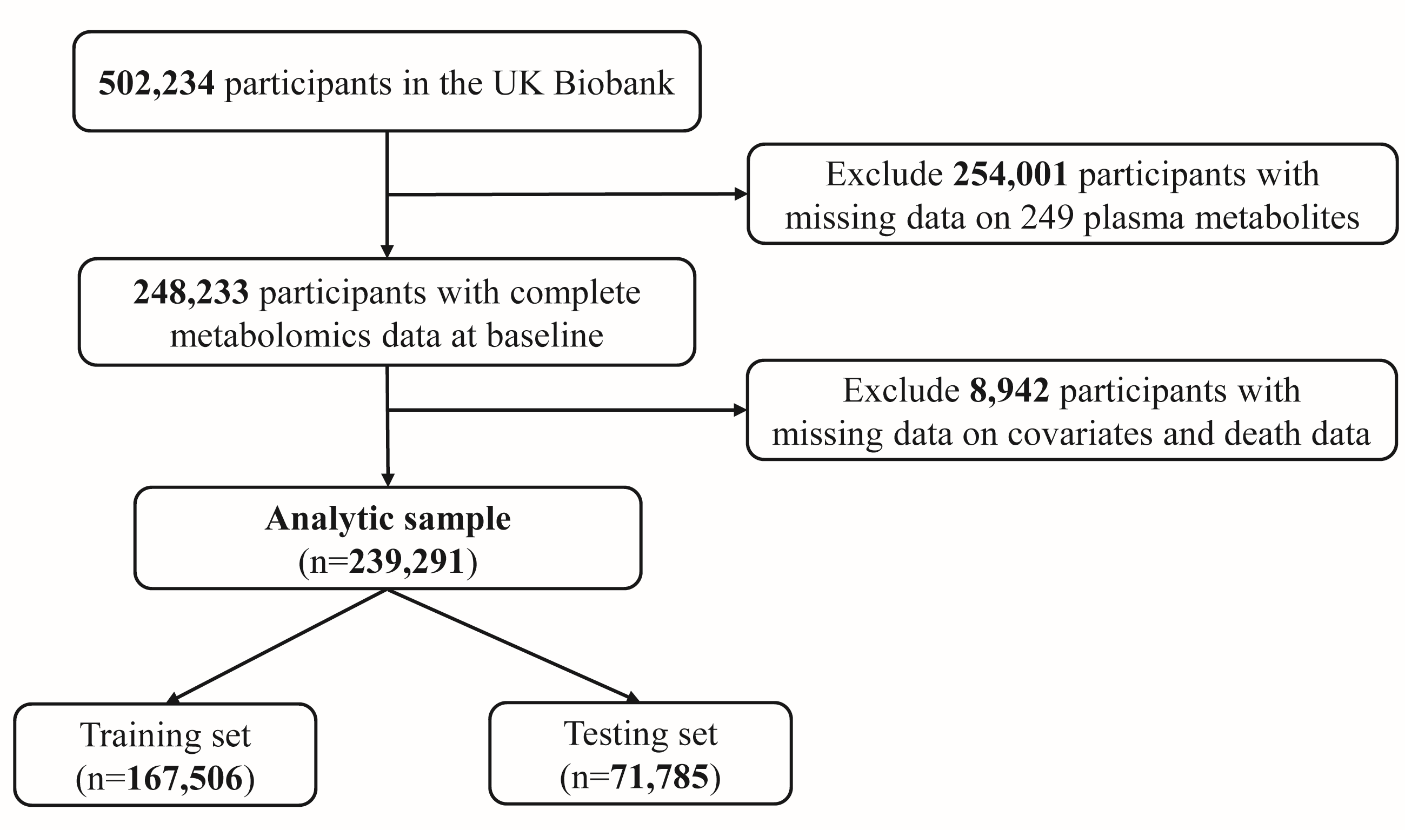


Figure S1. The f**lowchar**t diagram of participant selection.


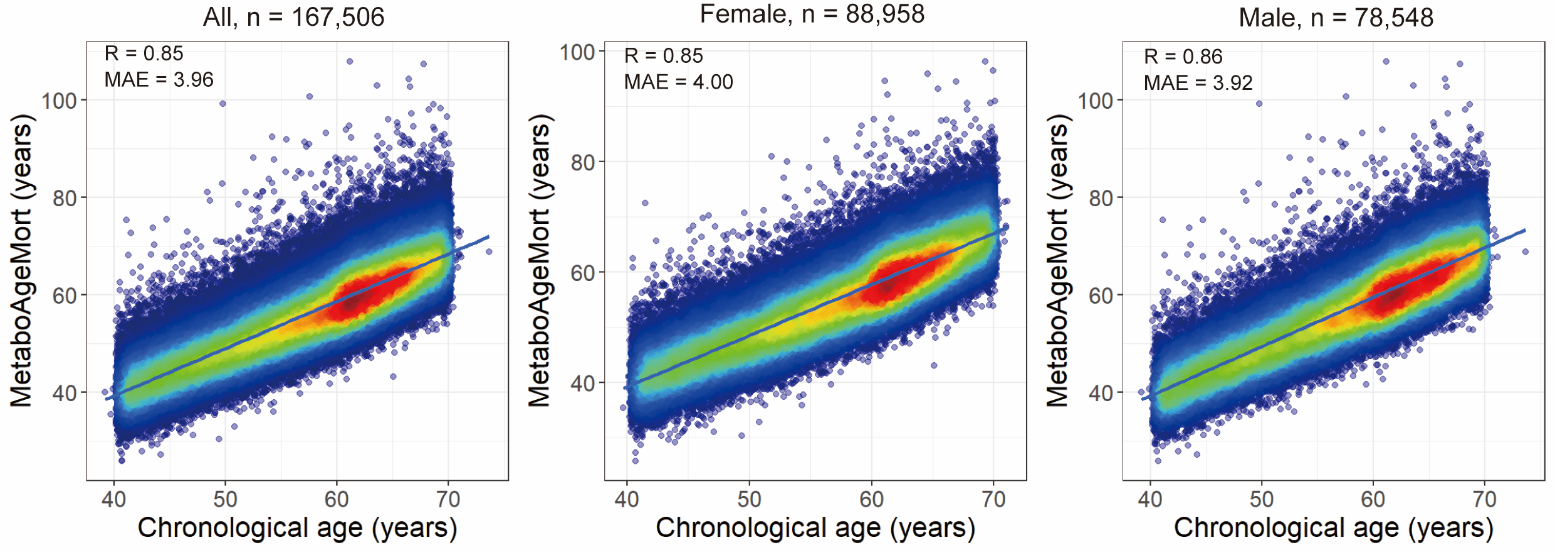


Figure S2. The distributions of MetaboAgeMort and chronological age across all participants and by sex in the training set.

Each scatter indicates a single participant. The correlation coefficient and MAE of the model are shown in the left top part of the plot. MAE, mean absolute error.


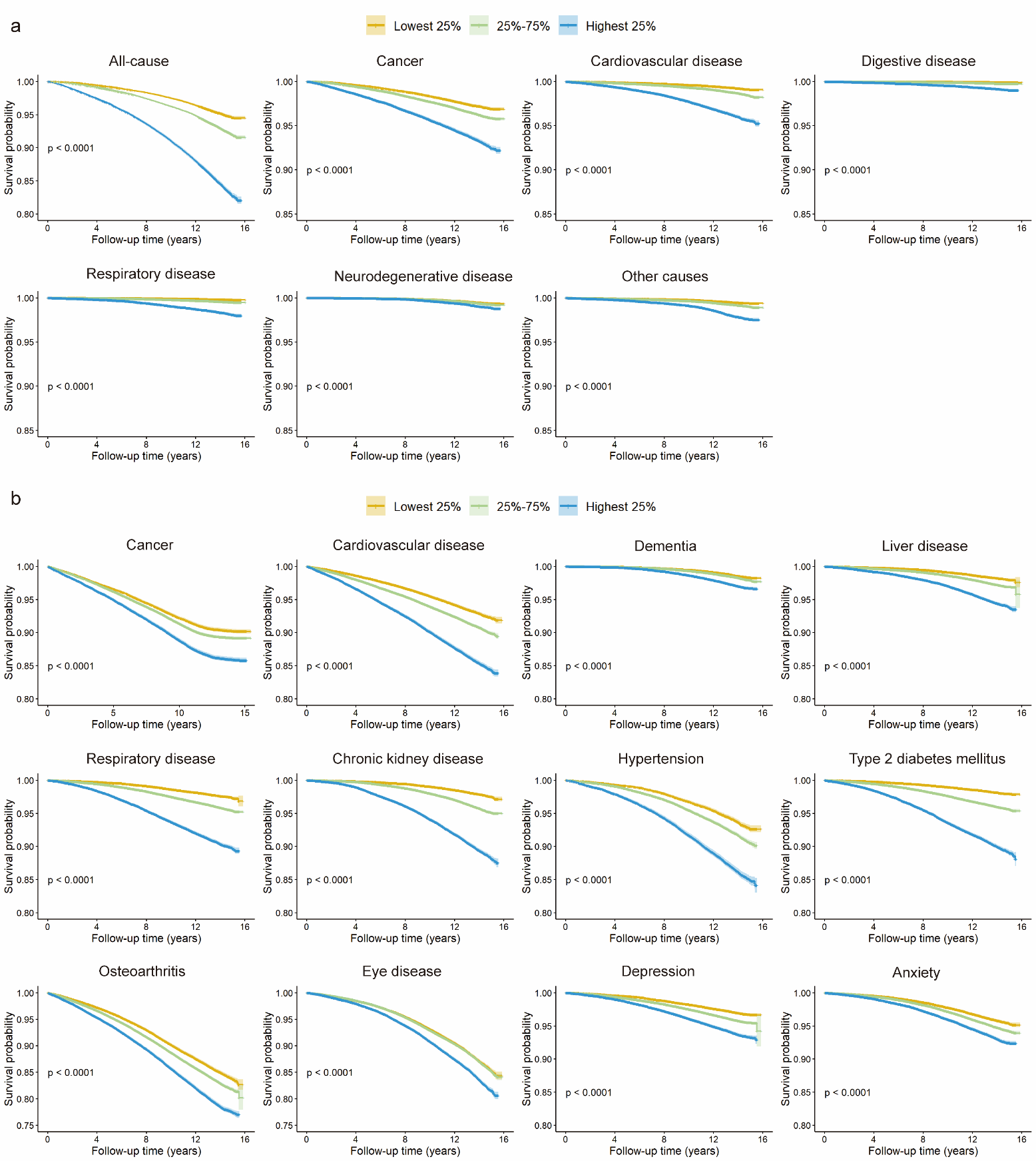


Figure S3. Kaplan-Meier survival plots of all-cause mortality, cause-specific mortality (a) and 12 aging-related diseases according to quartiles of the MetaboAgeMort Acceleration (b) in the training set.

Blue indicates the top quartile, green indicates the second and third quartile, and yellow indicates the bottom quartile. The y-axis indicates the survival rate, and the x-axis indicates follow-up time (in years).


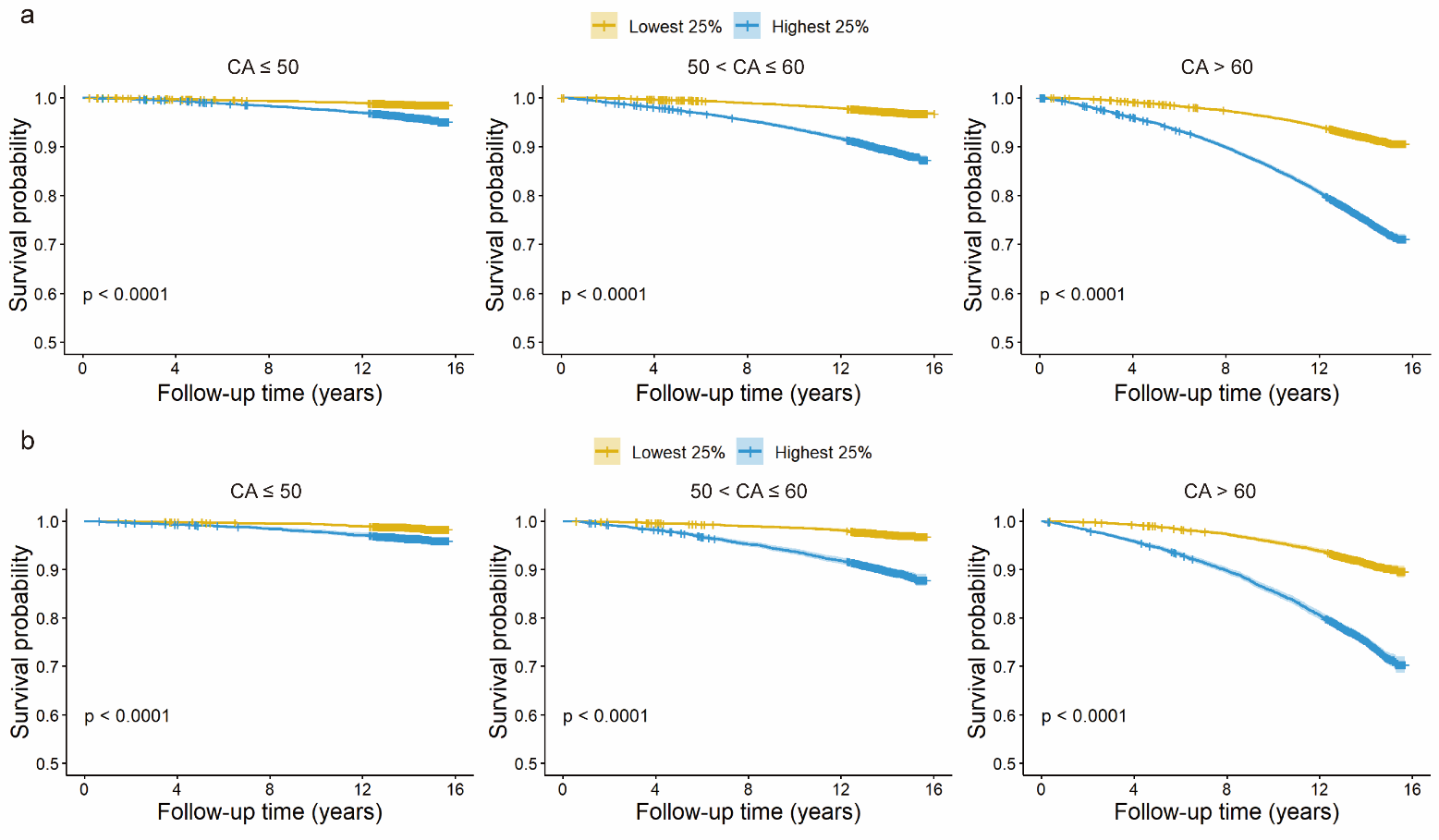


Figure S4. Kaplan-Meier curves for persons in the top quartile versus the bottom quartile of the MetaboAgeMort Acceleration in the training set (a) and testing set (b) across different chronological age subgroups.

Blue indicates the top quartile, and yellow indicates the bottom quartile. The y-axis indicates the survival rate, and the x-axis indicates follow-up time (in years). CA, chronological age.

**
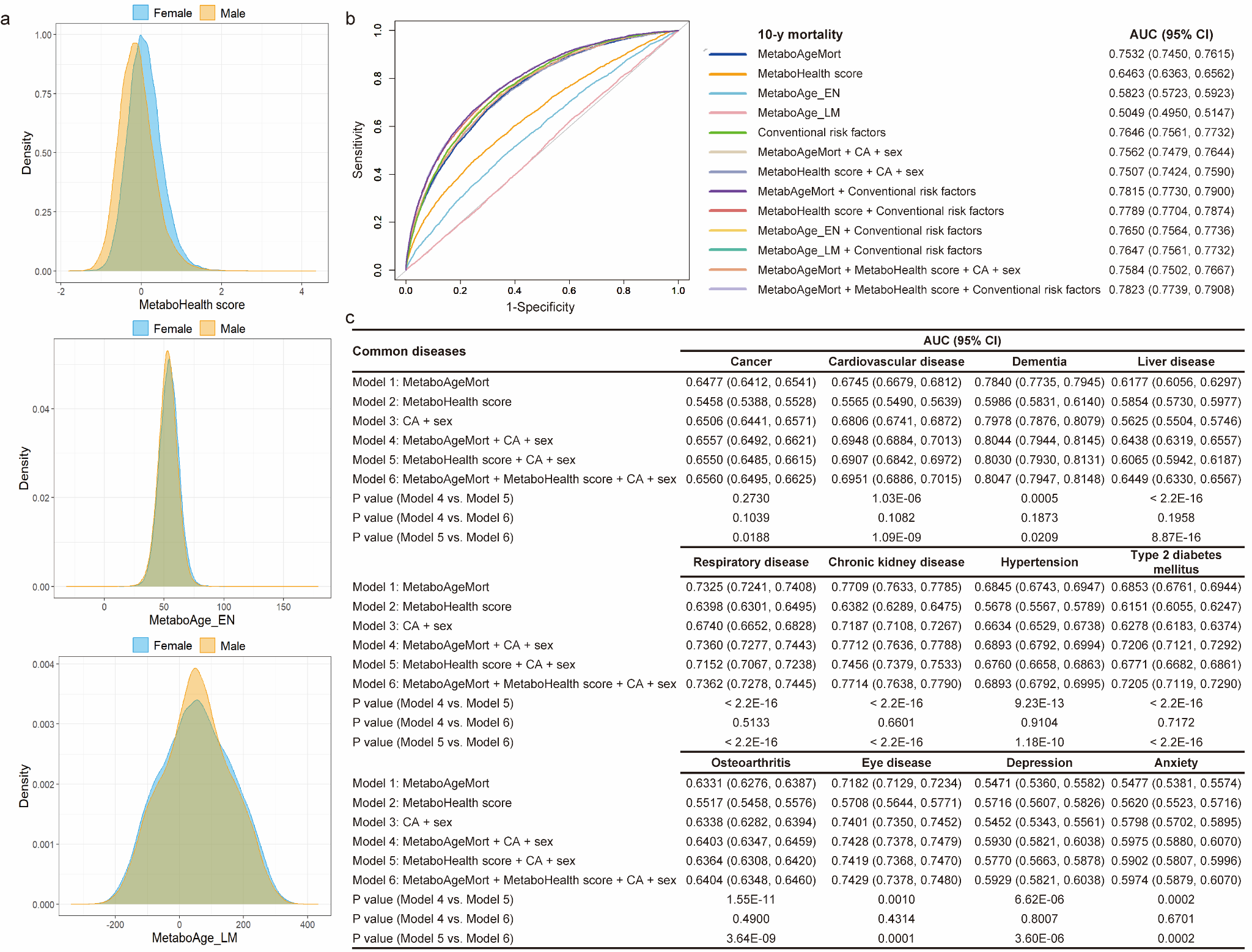
**

Figure S5. The comparison of predictive performance of MetaboAgeMort with MetaboAge and MetaboHealth score in the testing set.

a. The distribution of the MetaboHealth score and MetaboAge in the testing set. Blue indicates the distribution of these scores in female, and yellow indicates the distribution of these scores in male. b. Receiver operating characteristic curves for 10-year all-cause mortality in the testing set. The conventional risk factors included chronological age, sex, alcohol intake frequency, smoking status, body mass index, systolic blood pressure, triglycerides, creatinine, total cholesterol, high-density lipoprotein cholesterol, and prevalent diabetes, cardiovascular and cancer. c. The area under the curve for incident diseases prediction in the testing set. CA, chronological age; AUC, area under the curve.

**
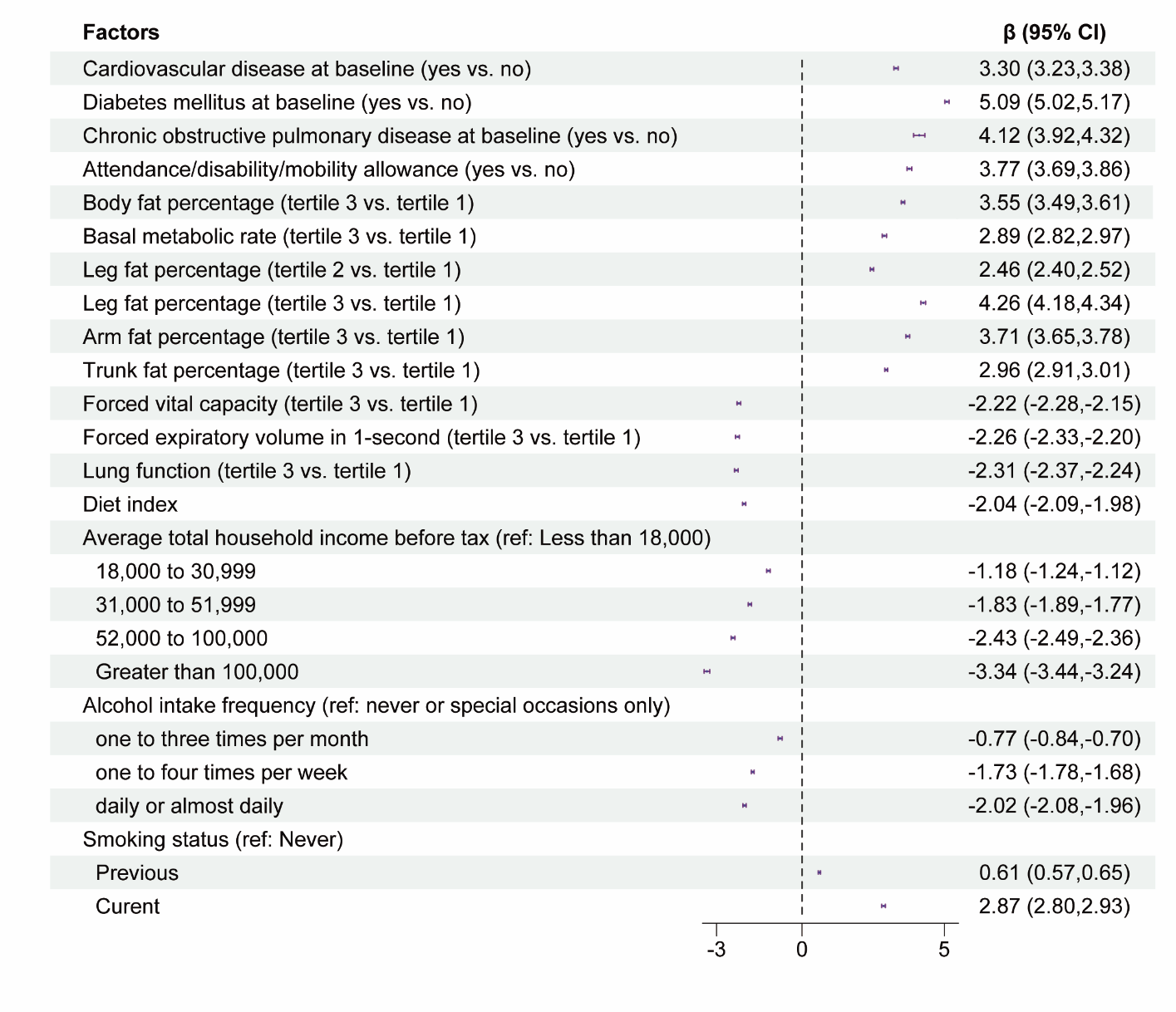
**

Figure S6. Forest plot for the estimated effects of the 16 modifiable factors that are quantitatively more strongly associated with MetaboAgeMort.

**
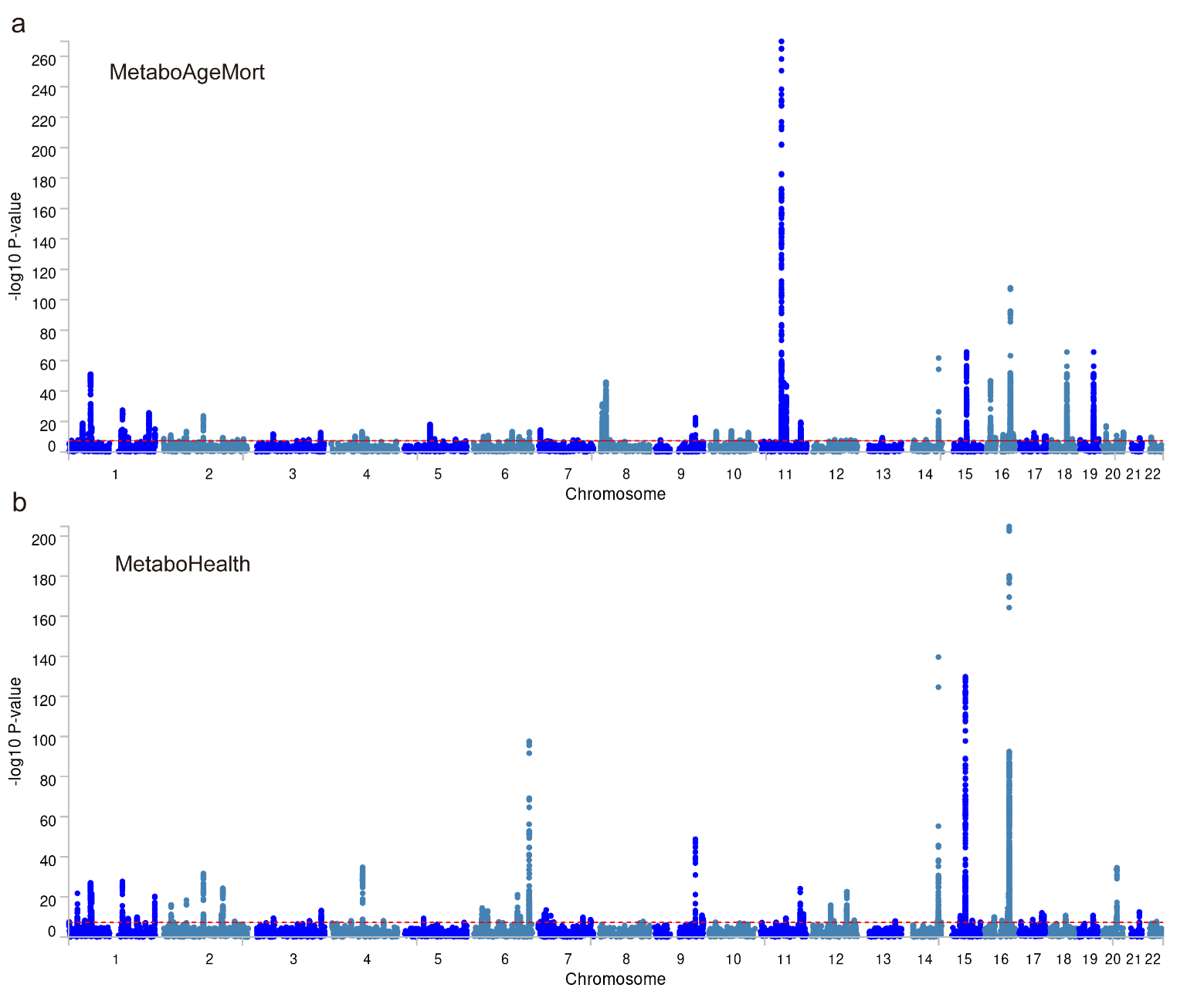
**

Figure S7. The Manhattan plot showing the results of genome-wide association analysis of MetaboAgeMort (a) and MetaboHealth score (b).


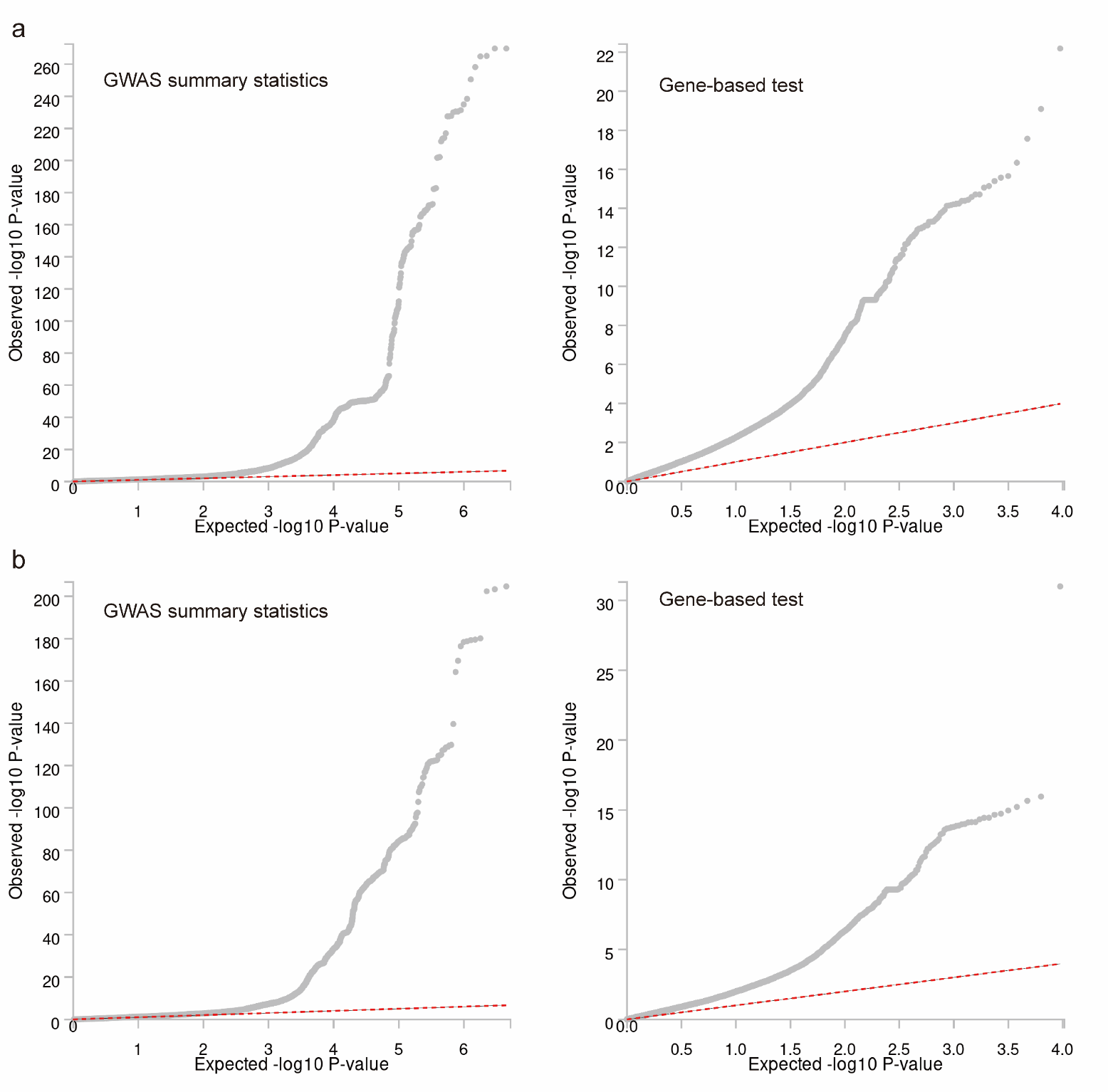


Figure S8. The Q-Q plot of genome-wide association analysis summary statistics and the gene-based test computed by MAGMA of MetaboAgeMort (a) and MetaboHealth score (b)


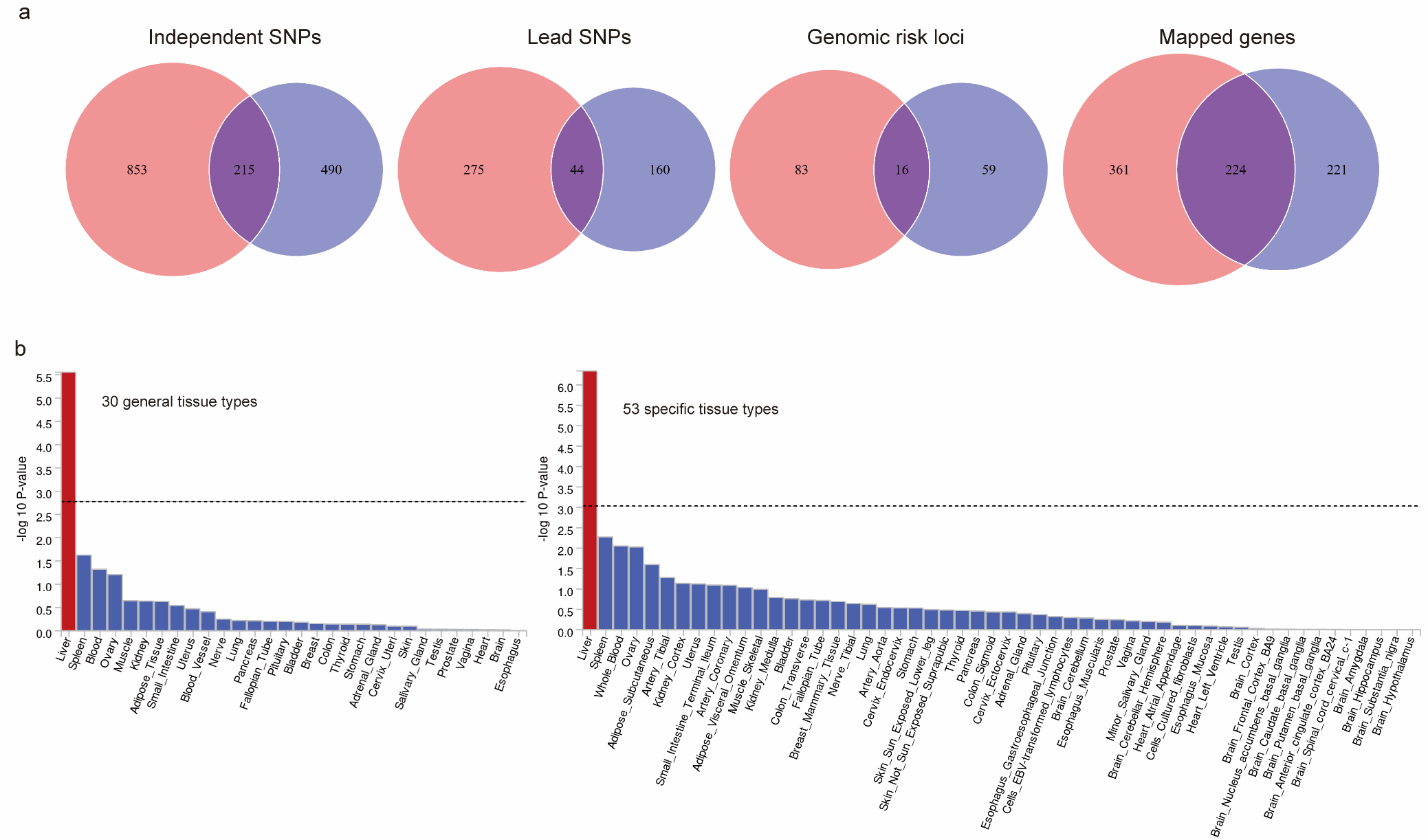


Figure S9. FUMA results overlap between MetaboAgeMort (red) and MetaboHealth score (blue) (a) and the gene-tissue expression results of MetaboHealth score (b).

Table S1. Baseline characteristics of study participants in total and by datasets.

|  | **Total**  **(*n* = 239,291)** | **Training set**  **(*n*=167,506)** | **Testing set**  **(*n*=71,785)** | **P value** |
| --- | --- | --- | --- | --- |
| **Age, years** | 58.3 (50.6, 63.7) | 58.3 (50.6, 63.7) | 58.3 (50.6, 63.7) | 0.854 |
| **Sex** |  |  |  |  |
| Female | 126,828 (53.0) | 88,958 (53.1) | 37,870 (52.8) | 0.114 |
| Male | 112,463 (47.0) | 78,548 (46.9) | 33,915 (47.2) |  |
| **Ethnicity** |  |  |  |  |
| Non-British White | 10,622 (4.4) | 7,361 (4.4) | 3,261 (4.5) | 0.109 |
| British White | 228,669 (95.6) | 160,145 (95.6) | 68,524 (95.5) |  |
| **Education level ^a^** |  |  |  |  |
| High | 77,389 (32.3) | 54,206 (32.4) | 23,183 (32.3) | 0.729 |
| Intermediate | 77,867 (32.5) | 54,559 (32.6) | 23,308 (32.5) |  |
| Low | 84,035 (35.1) | 58,741 (35.1) | 25,294 (35.2) |  |
| **Townsend Deprivation Index** | -2.2 (-3.7, 0.3) | -2.2 (-3.7, 0.3) | -2.2 (-3.7, 0.3) | 0.634 |
| **Smoking status** |  |  |  |  |
| Never smoker | 131,054 (54.8) | 91,781 (54.8) | 39,273 (54.7) | 0.359 |
| Previous smoker | 83,410 (34.9) | 58,443 (34.9) | 24,967 (34.8) |  |
| Current smoker | 24,827 (10.4) | 17,282 (10.3) | 7,545 (10.5) |  |
| **Alcohol intake frequency** |  |  |  |  |
| Never or special occasions | 45,625 (19.1) | 31,828 (19.0) | 13,797 (19.1) | 0.511 |
| 1 to 3 times per month | 27,188 (11.4) | 19,024 (11.4) | 8,164 (11.4) |  |
| 1 to 4 times per week | 119,083 (49.8) | 83,511 (49.9) | 35,572 (49.6) |  |
| Daily or almost daily | 47,395 (19.8) | 33,143 (19.8) | 14,252 (19.9) |  |
| **Regular exercise** |  |  |  |  |
| No | 108,746 (45.4) | 75,987 (45.4) | 32,759 (45.6) | 0.224 |
| Yes | 130,545 (54.6) | 91,519 (54.6) | 39,026 (54.4) |  |
| **Healthy diet** |  |  |  |  |
| No | 49,268 (20.6) | 34,504 (20.6) | 14,764 (20.6) | 0.865 |
| Yes | 190,023 (79.4) | 133,002 (79.4) | 57,021 (79.4) |  |
| **Body mass index** | 26.8 (24.3, 30.0) | 26.8 (24.3, 30.0) | 26.8 (24.3, 30.0) | 0.549 |
| **Cholesterol-lowering medication** |  |  |  |  |
| No | 198,167 (82.8) | 138,764 (82.8) | 59,403 (82.8) | 0.597 |
| Yes | 41,124 (17.2) | 28,742 (17.2) | 12,382 (17.2) |  |
| **Anti-hypertensive medication** |  |  |  |  |
| No | 189,720 (79.3) | 132,884 (79.3) | 56,836 (79.2) | 0.393 |
| Yes | 49,571 (20.7) | 34,622 (20.7) | 14,949 (20.8) |  |
| **Anti-diabetes medication** |  |  |  |  |
| No | 236,957 (99.0) | 165,878 (99.0) | 71,079 (99.0) | 0.809 |
| Yes | 2,334 (1.0) | 1,628 (1.0) | 706 (1.0) |  |

Notes: Continuous and categorical variables were described using median (inter-quartile range, IQR) and number (percentage), respectively. The P values were calculated using the Kruskal-Wallis test for continuous variables, and the Chi-square test for categorical variables.

^a^ Education level was classified as high (college or university degree), intermediate (A/AS levels or equivalent, O levels/General Certificate of Secondary Education levels or equivalent), and low (none of the above).

Table S2. Associations of 249 plasma metabolic biomarkers with all-cause mortality in the total participants.

| **Field ID** | **Title** | **Group** | **Model 1** ^a^ | | **Model 2** ^b^ | |
| --- | --- | --- | --- | --- | --- | --- |
|  |  |  | **HR (95% CI)** | **P value** | **HR (95% CI)** | **P value** |
| **23400** | Total cholesterol | Cholesterol | 0.82 (0.81, 0.83) | 2.49E-182 | 0.94 (0.92, 0.95) | 1.91E-16 |
| **23401** | Total cholesterol minus HDL-C | Cholesterol | 0.84 (0.83, 0.85) | 1.90E-150 | 0.93 (0.92, 0.94) | 1.51E-20 |
| **23402** | Remnant cholesterol (non-HDL, non-LDL -cholesterol) | Cholesterol | 0.86 (0.85, 0.87) | 1.33E-106 | 0.95 (0.94, 0.97) | 2.74E-09 |
| **23403** | VLDL cholesterol | Cholesterol | 0.91 (0.90, 0.92) | 5.84E-39 | 0.96 (0.95, 0.97) | 1.16E-07 |
| **23404** | Clinical LDL cholesterol | Cholesterol | 0.82 (0.81, 0.83) | 7.38E-189 | 0.92 (0.91, 0.93) | 1.64E-27 |
| **23405** | LDL cholesterol | Cholesterol | 0.82 (0.81, 0.83) | 2.90E-178 | 0.91 (0.9, 0.93) | 1.38E-31 |
| **23406** | HDL cholesterol | Cholesterol | 0.86 (0.85, 0.88) | 4.46E-75 | 1.00 (0.98, 1.02) | 8.98E-01 |
| **23407** | Total triglycerides | Triglycerides | 1.02 (1.00, 1.03) | 1.71E-02 | 0.96 (0.94, 0.97) | 5.33E-09 |
| **23408** | Triglycerides in VLDL | Triglycerides | 1.00 (0.99, 1.02) | 5.54E-01 | 0.94 (0.93, 0.96) | 3.80E-15 |
| **23409** | Triglycerides in LDL | Triglycerides | 1.07 (1.05, 1.08) | 2.56E-20 | 1.02 (1.01, 1.04) | 9.97E-04 |
| **23410** | Triglycerides in HDL | Triglycerides | 1.06 (1.04, 1.07) | 3.46E-15 | 1.01 (0.99, 1.02) | 2.49E-01 |
| **23411** | Total phospholipids in lipoprotein particles | Phospholipids | 0.86 (0.85, 0.87) | 2.85E-95 | 0.96 (0.95, 0.98) | 3.97E-07 |
| **23412** | Phospholipids in VLDL | Phospholipids | 0.96 (0.94, 0.97) | 3.89E-09 | 0.96 (0.94, 0.97) | 2.99E-08 |
| **23413** | Phospholipids in LDL | Phospholipids | 0.83 (0.82, 0.85) | 4.35E-146 | 0.92 (0.91, 0.94) | 9.20E-26 |
| **23414** | Phospholipids in HDL | Phospholipids | 0.91 (0.90, 0.93) | 1.64E-31 | 1.00 (0.99, 1.02) | 5.75E-01 |
| **23415** | Total esterified cholesterol | Cholesteryl esters | 0.82 (0.81, 0.83) | 5.68E-190 | 0.93 (0.92, 0.95) | 2.59E-17 |
| **23416** | Cholesteryl esters in VLDL | Cholesteryl esters | 0.90 (0.89, 0.91) | 4.04E-51 | 0.97 (0.95, 0.98) | 1.92E-05 |
| **23417** | Cholesteryl esters in LDL | Cholesteryl esters | 0.83 (0.82, 0.84) | 7.04E-162 | 0.91 (0.90, 0.93) | 6.20E-32 |
| **23418** | Cholesteryl esters in HDL | Cholesteryl esters | 0.86 (0.85, 0.87) | 4.87E-79 | 0.99 (0.98, 1.01) | 3.62E-01 |
| **23419** | Total free cholesterol | Free cholesterol | 0.83 (0.82, 0.85) | 9.63E-148 | 0.94 (0.93, 0.96) | 5.27E-13 |
| **23420** | Free cholesterol in VLDL | Free cholesterol | 0.94 (0.92, 0.95) | 2.63E-20 | 0.95 (0.94, 0.97) | 1.05E-09 |
| **23421** | Free cholesterol in LDL | Free cholesterol | 0.81 (0.80, 0.83) | 1.69E-190 | 0.92 (0.91, 0.94) | 2.88E-25 |
| **23422** | Free cholesterol in HDL | Free cholesterol | 0.89 (0.88, 0.90) | 1.14E-46 | 1.03 (1.01, 1.05) | 8.05E-04 |
| **23423** | Total lipids in lipoprotein particles | Total lipids | 0.86 (0.85, 0.87) | 2.46E-101 | 0.94 (0.93, 0.96) | 2.55E-15 |
| **23424** | Total lipids in VLDL | Total lipids | 0.96 (0.95, 0.98) | 3.06E-07 | 0.95 (0.93, 0.96) | 1.92E-13 |
| **23425** | Total lipids in LDL | Total lipids | 0.83 (0.82, 0.85) | 7.58E-156 | 0.92 (0.91, 0.93) | 1.41E-27 |
| **23426** | Total lipids in HDL | Total lipids | 0.89 (0.88, 0.90) | 5.24E-49 | 1.00 (0.98, 1.02) | 8.81E-01 |
| **23427** | Total concentration of lipoprotein particles | Lipoprotein particle concentrations | 0.84 (0.83, 0.85) | 1.08E-114 | 0.93 (0.92, 0.95) | 4.25E-18 |
| **23428** | Concentration of VLDL particles | Lipoprotein particle concentrations | 0.95 (0.93, 0.96) | 2.47E-14 | 0.97 (0.96, 0.99) | 8.72E-05 |
| **23429** | Concentration of LDL particles | Lipoprotein particle concentrations | 0.86 (0.85, 0.87) | 1.99E-95 | 0.94 (0.93, 0.96) | 1.77E-13 |
| **23430** | Concentration of HDL particles | Lipoprotein particle concentrations | 0.85 (0.84, 0.87) | 3.13E-93 | 0.94 (0.92, 0.95) | 1.27E-15 |
| **23431** | Average diameter for VLDL particles | Lipoprotein particle sizes | 0.99 (0.97, 1.00) | 1.03E-01 | 0.90 (0.88, 0.91) | 8.51E-46 |
| **23432** | Average diameter for LDL particles | Lipoprotein particle sizes | 0.93 (0.92, 0.95) | 2.47E-23 | 1.02 (1.01, 1.04) | 2.34E-03 |
| **23433** | Average diameter for HDL particles | Lipoprotein particle sizes | 0.97 (0.96, 0.99) | 2.92E-04 | 1.13 (1.11, 1.15) | 4.48E-45 |
| **23434** | Phosphoglycerides | Other lipids | 0.88 (0.87, 0.89) | 2.93E-69 | 0.96 (0.95, 0.98) | 1.44E-06 |
| **23435** | Ratio of triglycerides to phosphoglycerides | Other lipids | 1.09 (1.07, 1.10) | 3.13E-30 | 0.97 (0.95, 0.98) | 9.04E-06 |
| **23436** | Total cholines | Other lipids | 0.86 (0.85, 0.87) | 2.59E-92 | 0.96 (0.95, 0.98) | 1.15E-06 |
| **23437** | Phosphatidylcholines | Other lipids | 0.86 (0.85, 0.87) | 1.60E-94 | 0.96 (0.94, 0.97) | 2.87E-08 |
| **23438** | Sphingomyelins | Other lipids | 0.88 (0.86, 0.89) | 6.89E-71 | 1.00 (0.99, 1.02) | 8.05E-01 |
| **23439** | Apolipoprotein B | Apolipoproteins | 0.86 (0.85, 0.87) | 9.17E-98 | 0.95 (0.93, 0.96) | 4.21E-12 |
| **23440** | Apolipoprotein A1 | Apolipoproteins | 0.88 (0.87, 0.89) | 4.21E-59 | 0.97 (0.96, 0.99) | 1.96E-03 |
| **23441** | Ratio of apolipoprotein B to apolipoprotein A1 | Apolipoproteins | 0.94 (0.92, 0.95) | 3.36E-20 | 0.97 (0.96, 0.99) | 4.01E-04 |
| **23442** | Total fatty acids | Fatty acids | 0.94 (0.93, 0.96) | 1.98E-15 | 0.96 (0.95, 0.98) | 2.86E-07 |
| **23443** | Degree of unsaturation | Fatty acids | 0.78 (0.77, 0.79) | 1.29E-265 | 0.89 (0.88, 0.91) | 1.25E-52 |
| **23444** | Omega-3 fatty acids | Fatty acids | 0.82 (0.81, 0.83) | 4.88E-148 | 0.88 (0.87, 0.90) | 2.35E-57 |
| **23445** | Omega-6 fatty acids | Fatty acids | 0.85 (0.83, 0.86) | 3.01E-121 | 0.93 (0.92, 0.94) | 4.22E-21 |
| **23446** | Polyunsaturated fatty acids | Fatty acids | 0.82 (0.81, 0.83) | 1.58E-162 | 0.91 (0.9, 0.92) | 5.48E-35 |
| **23447** | Monounsaturated fatty acids | Fatty acids | 1.05 (1.03, 1.06) | 1.20E-10 | 0.99 (0.98, 1.01) | 4.32E-01 |
| **23448** | Saturated fatty acids | Fatty acids | 0.98 (0.97, 1.00) | 1.20E-02 | 0.99 (0.97, 1.00) | 3.68E-02 |
| **23449** | Linoleic acid | Fatty acids | 0.84 (0.83, 0.85) | 8.03E-133 | 0.93 (0.92, 0.95) | 2.17E-19 |
| **23450** | Docosahexaenoic acid | Fatty acids | 0.79 (0.78, 0.81) | 6.21E-194 | 0.89 (0.88, 0.91) | 5.65E-46 |
| **23451** | Ratio of omega-3 fatty acids to total fatty acids | Fatty acids | 0.82 (0.80, 0.83) | 3.23E-180 | 0.87 (0.86, 0.88) | 6.02E-75 |
| **23452** | Ratio of omega-6 fatty acids to total fatty acids | Fatty acids | 0.88 (0.86, 0.89) | 7.75E-90 | 0.97 (0.96, 0.99) | 1.84E-04 |
| **23453** | Ratio of polyunsaturated fatty acids to total fatty acids | Fatty acids | 0.83 (0.82, 0.84) | 3.26E-193 | 0.93 (0.92, 0.95) | 3.62E-23 |
| **23454** | Ratio of monounsaturated fatty acids to total fatty acids | Fatty acids | 1.24 (1.22, 1.26) | 2.69E-199 | 1.06 (1.04, 1.08) | 3.64E-14 |
| **23455** | Ratio of saturated fatty acids to total fatty acids | Fatty acids | 1.11 (1.10, 1.13) | 1.55E-55 | 1.06 (1.05, 1.08) | 2.15E-18 |
| **23456** | Ratio of linoleic acid to total fatty acids | Fatty acids | 0.83 (0.82, 0.84) | 6.21E-170 | 0.95 (0.94, 0.97) | 2.43E-10 |
| **23457** | Ratio of docosahexaenoic acid to total fatty acids | Fatty acids | 0.82 (0.81, 0.84) | 2.17E-167 | 0.91 (0.90, 0.92) | 4.32E-37 |
| **23458** | Ratio of polyunsaturated fatty acids to monounsaturated fatty acids | Fatty acids | 0.81 (0.8, 0.82) | 3.97E-208 | 0.93 (0.92, 0.95) | 1.04E-19 |
| **23459** | Ratio of omega-6 fatty acids to omega-3 fatty acids | Fatty acids | 1.16 (1.15, 1.18) | 1.39E-106 | 1.13 (1.11, 1.14) | 1.83E-61 |
| **23460** | Alanine | Amino acids | 1.00 (0.99, 1.02) | 7.88E-01 | 0.96 (0.95, 0.98) | 9.61E-08 |
| **23461** | Glutamine | Amino acids | 0.93 (0.92, 0.95) | 2.91E-23 | 0.96 (0.95, 0.98) | 3.06E-07 |
| **23462** | Glycine | Amino acids | 0.97 (0.96, 0.99) | 5.48E-04 | 1.01 (1.00, 1.03) | 9.83E-02 |
| **23463** | Histidine | Amino acids | 0.90 (0.89, 0.92) | 1.06E-40 | 0.93 (0.91, 0.94) | 3.84E-23 |
| **23464** | Total concentration of branched-chain amino acids (leucine + isoleucine + valine) | Amino acids | 0.96 (0.94, 0.97) | 1.12E-09 | 0.92 (0.91, 0.93) | 9.84E-28 |
| **23465** | Isoleucine | Amino acids | 0.99 (0.98, 1.01) | 4.72E-01 | 0.96 (0.95, 0.98) | 7.56E-07 |
| **23466** | Leucine | Amino acids | 0.95 (0.93, 0.96) | 6.37E-13 | 0.92 (0.91, 0.93) | 2.71E-27 |
| **23467** | Valine | Amino acids | 0.95 (0.94, 0.97) | 2.24E-11 | 0.91 (0.90, 0.93) | 1.47E-33 |
| **23468** | Phenylalanine | Amino acids | 1.07 (1.06, 1.08) | 7.21E-31 | 1.05 (1.03, 1.06) | 1.61E-12 |
| **23469** | Tyrosine | Amino acids | 1.04 (1.02, 1.05) | 4.59E-07 | 1.03 (1.01, 1.04) | 6.72E-05 |
| **23470** | Glucose | Glycolysis related metabolites | 1.14 (1.13, 1.16) | 4.02E-101 | 1.04 (1.02, 1.05) | 3.54E-08 |
| **23471** | Lactate | Glycolysis related metabolites | 1.06 (1.04, 1.07) | 4.68E-14 | 1.01 (0.99, 1.02) | 2.10E-01 |
| **23472** | Pyruvate | Glycolysis related metabolites | 1.07 (1.05, 1.08) | 1.42E-21 | 1.04 (1.02, 1.05) | 2.91E-07 |
| **23473** | Citrate | Glycolysis related metabolites | 1.04 (1.02, 1.05) | 7.82E-08 | 1.04 (1.03, 1.06) | 1.45E-09 |
| **23474** | 3-Hydroxybutyrate | Ketone bodies | 1.10 (1.08, 1.11) | 1.52E-54 | 1.09 (1.08, 1.10) | 2.33E-45 |
| **23475** | Acetate | Ketone bodies | 1.00 (0.98, 1.01) | 6.62E-01 | 1.02 (1.01, 1.03) | 4.92E-05 |
| **23476** | Acetoacetate | Ketone bodies | 1.10 (1.09, 1.11) | 6.86E-71 | 1.08 (1.07, 1.09) | 1.86E-45 |
| **23477** | Acetone | Ketone bodies | 1.07 (1.06, 1.08) | 3.75E-28 | 1.07 (1.05, 1.08) | 5.19E-29 |
| **23478** | Creatinine | Fluid balance | 1.10 (1.09, 1.11) | 9.45E-75 | 1.06 (1.04, 1.07) | 1.85E-20 |
| **23479** | Albumin | Fluid balance | 0.82 (0.81, 0.83) | 2.30E-181 | 0.87 (0.86, 0.88) | 3.09E-88 |
| **23480** | Glycoprotein acetyls | Inflammation | 1.25 (1.24, 1.27) | 2.11E-229 | 1.11 (1.10, 1.13) | 9.47E-46 |
| **23481** | Concentration of chylomicrons and extremely large VLDL particles | Lipoprotein subclasses | 1.05 (1.04, 1.07) | 3.16E-14 | 0.98 (0.96, 0.99) | 2.09E-03 |
| **23482** | Total lipids in chylomicrons and extremely large VLDL | Lipoprotein subclasses | 1.05 (1.04, 1.06) | 4.39E-12 | 0.97 (0.96, 0.99) | 2.39E-04 |
| **23483** | Phospholipids in chylomicrons and extremely large VLDL | Lipoprotein subclasses | 1.06 (1.04, 1.07) | 1.13E-15 | 0.98 (0.96, 0.99) | 2.81E-03 |
| **23484** | Cholesterol in chylomicrons and extremely large VLDL | Lipoprotein subclasses | 1.04 (1.03, 1.05) | 3.01E-08 | 0.98 (0.96, 0.99) | 1.86E-03 |
| **23485** | Cholesteryl esters in chylomicrons and extremely large VLDL | Lipoprotein subclasses | 1.03 (1.02, 1.05) | 8.19E-06 | 0.98 (0.96, 0.99) | 2.86E-03 |
| **23486** | Free cholesterol in chylomicrons and extremely large VLDL | Lipoprotein subclasses | 1.05 (1.03, 1.06) | 1.07E-11 | 0.98 (0.96, 0.99) | 1.86E-03 |
| **23487** | Triglycerides in chylomicrons and extremely large VLDL | Lipoprotein subclasses | 1.05 (1.04, 1.06) | 9.14E-13 | 0.97 (0.96, 0.99) | 2.21E-04 |
| **23488** | Concentration of very large VLDL particles | Lipoprotein subclasses | 1.02 (1.00, 1.03) | 2.98E-02 | 0.95 (0.94, 0.97) | 2.10E-10 |
| **23489** | Total lipids in very large VLDL | Lipoprotein subclasses | 1.01 (0.99, 1.02) | 2.10E-01 | 0.95 (0.93, 0.96) | 9.50E-13 |
| **23490** | Phospholipids in very large VLDL | Lipoprotein subclasses | 1.01 (1.00, 1.02) | 1.88E-01 | 0.96 (0.94, 0.97) | 2.00E-09 |
| **23491** | Cholesterol in very large VLDL | Lipoprotein subclasses | 0.97 (0.96, 0.98) | 3.90E-05 | 0.95 (0.93, 0.96) | 7.63E-12 |
| **23492** | Cholesteryl esters in very large VLDL | Lipoprotein subclasses | 0.94 (0.93, 0.96) | 4.67E-15 | 0.94 (0.93, 0.96) | 2.31E-13 |
| **23493** | Free cholesterol in very large VLDL | Lipoprotein subclasses | 1.00 (0.99, 1.01) | 9.54E-01 | 0.96 (0.94, 0.97) | 1.74E-09 |
| **23494** | Triglycerides in very large VLDL | Lipoprotein subclasses | 1.02 (1.01, 1.04) | 6.46E-04 | 0.95 (0.94, 0.96) | 2.62E-12 |
| **23495** | Concentration of large VLDL particles | Lipoprotein subclasses | 0.99 (0.98, 1.01) | 2.27E-01 | 0.94 (0.93, 0.96) | 3.23E-14 |
| **23496** | Total lipids in large VLDL | Lipoprotein subclasses | 0.97 (0.96, 0.99) | 2.93E-04 | 0.93 (0.92, 0.94) | 1.48E-21 |
| **23497** | Phospholipids in large VLDL | Lipoprotein subclasses | 1.00 (0.98, 1.01) | 7.36E-01 | 0.95 (0.93, 0.96) | 2.02E-13 |
| **23498** | Cholesterol in large VLDL | Lipoprotein subclasses | 0.96 (0.95, 0.97) | 8.86E-09 | 0.94 (0.93, 0.96) | 1.67E-15 |
| **23499** | Cholesteryl esters in large VLDL | Lipoprotein subclasses | 0.94 (0.92, 0.95) | 1.36E-18 | 0.94 (0.93, 0.96) | 1.63E-13 |
| **23500** | Free cholesterol in large VLDL | Lipoprotein subclasses | 0.98 (0.97, 1.00) | 1.09E-02 | 0.94 (0.93, 0.95) | 4.81E-16 |
| **23501** | Triglycerides in large VLDL | Lipoprotein subclasses | 0.98 (0.96, 0.99) | 1.63E-03 | 0.93 (0.91, 0.94) | 2.30E-24 |
| **23502** | Concentration of medium VLDL particles | Lipoprotein subclasses | 0.89 (0.88, 0.90) | 5.05E-58 | 0.94 (0.92, 0.95) | 6.17E-18 |
| **23503** | Total lipids in medium VLDL | Lipoprotein subclasses | 0.90 (0.88, 0.91) | 2.07E-52 | 0.93 (0.91, 0.94) | 1.13E-24 |
| **23504** | Phospholipids in medium VLDL | Lipoprotein subclasses | 0.89 (0.87, 0.90) | 2.11E-63 | 0.94 (0.92, 0.95) | 1.45E-16 |
| **23505** | Cholesterol in medium VLDL | Lipoprotein subclasses | 0.85 (0.84, 0.86) | 3.36E-116 | 0.95 (0.93, 0.96) | 1.55E-11 |
| **23506** | Cholesteryl esters in medium VLDL | Lipoprotein subclasses | 0.84 (0.83, 0.85) | 4.34E-131 | 0.96 (0.94, 0.97) | 8.58E-08 |
| **23507** | Free cholesterol in medium VLDL | Lipoprotein subclasses | 0.87 (0.86, 0.88) | 3.79E-79 | 0.94 (0.93, 0.96) | 7.95E-15 |
| **23508** | Triglycerides in medium VLDL | Lipoprotein subclasses | 0.96 (0.94, 0.97) | 1.19E-09 | 0.93 (0.91, 0.94) | 2.44E-25 |
| **23509** | Concentration of small VLDL particles | Lipoprotein subclasses | 0.97 (0.95, 0.98) | 1.38E-06 | 0.97 (0.95, 0.98) | 2.48E-06 |
| **23510** | Total lipids in small VLDL | Lipoprotein subclasses | 0.95 (0.94, 0.96) | 1.51E-12 | 0.96 (0.94, 0.97) | 2.12E-08 |
| **23511** | Phospholipids in small VLDL | Lipoprotein subclasses | 0.91 (0.90, 0.92) | 7.42E-40 | 0.95 (0.94, 0.97) | 8.27E-10 |
| **23512** | Cholesterol in small VLDL | Lipoprotein subclasses | 0.91 (0.90, 0.92) | 1.98E-39 | 0.96 (0.95, 0.98) | 4.11E-06 |
| **23513** | Cholesteryl esters in small VLDL | Lipoprotein subclasses | 0.93 (0.92, 0.94) | 3.21E-24 | 0.97 (0.96, 0.99) | 6.20E-04 |
| **23514** | Free cholesterol in small VLDL | Lipoprotein subclasses | 0.88 (0.87, 0.89) | 1.68E-69 | 0.95 (0.94, 0.97) | 1.58E-10 |
| **23515** | Triglycerides in small VLDL | Lipoprotein subclasses | 1.02 (1.01, 1.04) | 1.78E-03 | 0.97 (0.95, 0.98) | 2.87E-06 |
| **23516** | Concentration of very small VLDL particles | Lipoprotein subclasses | 0.96 (0.94, 0.97) | 2.26E-10 | 1.03 (1.02, 1.05) | 3.12E-05 |
| **23517** | Total lipids in very small VLDL | Lipoprotein subclasses | 0.96 (0.95, 0.97) | 1.84E-08 | 1.04 (1.02, 1.05) | 1.56E-06 |
| **23518** | Phospholipids in very small VLDL | Lipoprotein subclasses | 0.99 (0.98, 1.01) | 2.34E-01 | 1.05 (1.04, 1.07) | 4.79E-11 |
| **23519** | Cholesterol in very small VLDL | Lipoprotein subclasses | 0.90 (0.89, 0.92) | 1.74E-45 | 1.03 (1.01, 1.04) | 8.01E-04 |
| **23520** | Cholesteryl esters in very small VLDL | Lipoprotein subclasses | 0.89 (0.87, 0.90) | 3.15E-62 | 1.02 (1.01, 1.04) | 8.94E-03 |
| **23521** | Free cholesterol in very small VLDL | Lipoprotein subclasses | 0.95 (0.94, 0.96) | 4.17E-13 | 1.04 (1.02, 1.05) | 1.52E-06 |
| **23522** | Triglycerides in very small VLDL | Lipoprotein subclasses | 1.08 (1.07, 1.10) | 5.67E-31 | 1.03 (1.01, 1.04) | 8.18E-05 |
| **23523** | Concentration of IDL particles | Lipoprotein subclasses | 0.86 (0.85, 0.88) | 4.42E-92 | 0.97 (0.95, 0.99) | 2.39E-04 |
| **23524** | Total lipids in IDL | Lipoprotein subclasses | 0.84 (0.83, 0.85) | 8.50E-133 | 0.97 (0.95, 0.99) | 2.48E-04 |
| **23525** | Phospholipids in IDL | Lipoprotein subclasses | 0.86 (0.85, 0.87) | 5.96E-102 | 0.99 (0.97, 1.01) | 1.82E-01 |
| **23526** | Cholesterol in IDL | Lipoprotein subclasses | 0.83 (0.81, 0.84) | 2.80E-165 | 0.96 (0.94, 0.97) | 4.41E-08 |
| **23527** | Cholesteryl esters in IDL | Lipoprotein subclasses | 0.82 (0.81, 0.83) | 1.61E-170 | 0.95 (0.93, 0.96) | 4.42E-10 |
| **23528** | Free cholesterol in IDL | Lipoprotein subclasses | 0.84 (0.83, 0.85) | 3.62E-130 | 0.98 (0.96, 0.99) | 2.73E-03 |
| **23529** | Triglycerides in IDL | Lipoprotein subclasses | 1.08 (1.07, 1.10) | 3.87E-30 | 1.05 (1.03, 1.06) | 2.79E-11 |
| **23530** | Concentration of large LDL particles | Lipoprotein subclasses | 0.86 (0.85, 0.87) | 5.50E-95 | 0.95 (0.94, 0.97) | 2.65E-10 |
| **23531** | Total lipids in large LDL | Lipoprotein subclasses | 0.83 (0.82, 0.84) | 7.53E-166 | 0.93 (0.91, 0.94) | 1.00E-22 |
| **23532** | Phospholipids in large LDL | Lipoprotein subclasses | 0.83 (0.82, 0.84) | 1.46E-159 | 0.93 (0.91, 0.94) | 4.86E-22 |
| **23533** | Cholesterol in large LDL | Lipoprotein subclasses | 0.82 (0.81, 0.83) | 1.02E-186 | 0.92 (0.91, 0.93) | 1.97E-26 |
| **23534** | Cholesteryl esters in large LDL | Lipoprotein subclasses | 0.82 (0.81, 0.83) | 3.30E-175 | 0.92 (0.91, 0.93) | 1.74E-27 |
| **23535** | Free cholesterol in large LDL | Lipoprotein subclasses | 0.81 (0.80, 0.82) | 6.75E-188 | 0.93 (0.91, 0.94) | 7.59E-20 |
| **23536** | Triglycerides in large LDL | Lipoprotein subclasses | 1.07 (1.06, 1.09) | 3.99E-24 | 1.04 (1.02, 1.05) | 3.14E-07 |
| **23537** | Concentration of medium LDL particles | Lipoprotein subclasses | 0.86 (0.85, 0.88) | 1.12E-90 | 0.93 (0.92, 0.95) | 1.25E-19 |
| **23538** | Total lipids in medium LDL | Lipoprotein subclasses | 0.85 (0.84, 0.86) | 9.03E-122 | 0.91 (0.90, 0.92) | 3.14E-34 |
| **23539** | Phospholipids in medium LDL | Lipoprotein subclasses | 0.85 (0.83, 0.86) | 2.10E-118 | 0.91 (0.90, 0.93) | 2.12E-33 |
| **23540** | Cholesterol in medium LDL | Lipoprotein subclasses | 0.84 (0.83, 0.85) | 6.54E-137 | 0.91 (0.89, 0.92) | 3.73E-37 |
| **23541** | Cholesteryl esters in medium LDL | Lipoprotein subclasses | 0.85 (0.84, 0.86) | 5.14E-113 | 0.91 (0.90, 0.92) | 4.70E-35 |
| **23542** | Free cholesterol in medium LDL | Lipoprotein subclasses | 0.82 (0.81, 0.83) | 1.08E-175 | 0.91 (0.90, 0.92) | 2.86E-34 |
| **23543** | Triglycerides in medium LDL | Lipoprotein subclasses | 1.06 (1.04, 1.07) | 1.79E-16 | 1.01 (1.00, 1.02) | 2.01E-01 |
| **23544** | Concentration of small LDL particles | Lipoprotein subclasses | 0.88 (0.87, 0.89) | 2.26E-73 | 0.95 (0.93, 0.96) | 5.22E-13 |
| **23545** | Total lipids in small LDL | Lipoprotein subclasses | 0.86 (0.85, 0.87) | 2.00E-101 | 0.93 (0.91, 0.94) | 9.26E-23 |
| **23546** | Phospholipids in small LDL | Lipoprotein subclasses | 0.87 (0.86, 0.88) | 2.73E-85 | 0.94 (0.93, 0.96) | 7.56E-14 |
| **23547** | Cholesterol in small LDL | Lipoprotein subclasses | 0.84 (0.83, 0.86) | 2.04E-124 | 0.92 (0.90, 0.93) | 1.70E-28 |
| **23548** | Cholesteryl esters in small LDL | Lipoprotein subclasses | 0.86 (0.85, 0.87) | 2.58E-101 | 0.92 (0.91, 0.93) | 1.78E-26 |
| **23549** | Free cholesterol in small LDL | Lipoprotein subclasses | 0.83 (0.82, 0.84) | 1.05E-158 | 0.92 (0.91, 0.94) | 4.65E-26 |
| **23550** | Triglycerides in small LDL | Lipoprotein subclasses | 1.05 (1.03, 1.06) | 9.05E-11 | 0.99 (0.98, 1.00) | 2.01E-01 |
| **23551** | Concentration of very large HDL particles | Lipoprotein subclasses | 0.95 (0.93, 0.96) | 2.99E-12 | 1.12 (1.10, 1.14) | 1.43E-40 |
| **23552** | Total lipids in very large HDL | Lipoprotein subclasses | 0.96 (0.94, 0.97) | 1.33E-07 | 1.13 (1.11, 1.15) | 1.35E-48 |
| **23553** | Phospholipids in very large HDL | Lipoprotein subclasses | 0.98 (0.96, 0.99) | 1.75E-03 | 1.14 (1.12, 1.16) | 5.27E-53 |
| **23554** | Cholesterol in very large HDL | Lipoprotein subclasses | 0.94 (0.92, 0.95) | 2.60E-16 | 1.12 (1.10, 1.14) | 6.83E-39 |
| **23555** | Cholesteryl esters in very large HDL | Lipoprotein subclasses | 0.93 (0.91, 0.94) | 4.41E-21 | 1.11 (1.09, 1.13) | 4.37E-33 |
| **23556** | Free cholesterol in very large HDL | Lipoprotein subclasses | 0.98 (0.96, 0.99) | 3.08E-03 | 1.14 (1.12, 1.16) | 4.37E-58 |
| **23557** | Triglycerides in very large HDL | Lipoprotein subclasses | 1.04 (1.03, 1.06) | 9.86E-09 | 1.04 (1.03, 1.06) | 2.37E-09 |
| **23558** | Concentration of large HDL particles | Lipoprotein subclasses | 0.92 (0.90, 0.93) | 2.14E-24 | 1.08 (1.06, 1.10) | 6.54E-19 |
| **23559** | Total lipids in large HDL | Lipoprotein subclasses | 0.92 (0.91, 0.94) | 1.78E-22 | 1.08 (1.07, 1.10) | 6.40E-20 |
| **23560** | Phospholipids in large HDL | Lipoprotein subclasses | 0.94 (0.92, 0.95) | 4.25E-15 | 1.08 (1.06, 1.10) | 2.85E-19 |
| **23561** | Cholesterol in large HDL | Lipoprotein subclasses | 0.91 (0.90, 0.93) | 6.68E-30 | 1.08 (1.06, 1.10) | 1.12E-18 |
| **23562** | Cholesteryl esters in large HDL | Lipoprotein subclasses | 0.91 (0.89, 0.92) | 4.89E-32 | 1.08 (1.06, 1.09) | 9.17E-17 |
| **23563** | Free cholesterol in large HDL | Lipoprotein subclasses | 0.93 (0.92, 0.95) | 2.13E-18 | 1.10 (1.08, 1.11) | 1.82E-25 |
| **23564** | Triglycerides in large HDL | Lipoprotein subclasses | 1.04 (1.02, 1.05) | 8.43E-07 | 1.05 (1.04, 1.07) | 5.27E-13 |
| **23565** | Concentration of medium HDL particles | Lipoprotein subclasses | 0.90 (0.88, 0.91) | 8.34E-42 | 0.98 (0.96, 0.99) | 5.89E-03 |
| **23566** | Total lipids in medium HDL | Lipoprotein subclasses | 0.91 (0.89, 0.92) | 9.37E-37 | 0.97 (0.96, 0.99) | 4.23E-04 |
| **23567** | Phospholipids in medium HDL | Lipoprotein subclasses | 0.93 (0.91, 0.94) | 2.55E-22 | 0.98 (0.96, 0.99) | 3.60E-03 |
| **23568** | Cholesterol in medium HDL | Lipoprotein subclasses | 0.88 (0.87, 0.90) | 1.03E-56 | 0.97 (0.95, 0.99) | 2.39E-04 |
| **23569** | Cholesteryl esters in medium HDL | Lipoprotein subclasses | 0.88 (0.87, 0.90) | 2.13E-56 | 0.97 (0.95, 0.98) | 6.86E-05 |
| **23570** | Free cholesterol in medium HDL | Lipoprotein subclasses | 0.89 (0.88, 0.91) | 2.75E-46 | 0.99 (0.97, 1.00) | 1.37E-01 |
| **23571** | Triglycerides in medium HDL | Lipoprotein subclasses | 1.05 (1.04, 1.07) | 6.48E-14 | 1.00 (0.99, 1.02) | 7.35E-01 |
| **23572** | Concentration of small HDL particles | Lipoprotein subclasses | 0.86 (0.84, 0.87) | 1.08E-101 | 0.88 (0.87, 0.90) | 7.32E-62 |
| **23573** | Total lipids in small HDL | Lipoprotein subclasses | 0.89 (0.88, 0.91) | 9.94E-54 | 0.90 (0.89, 0.91) | 1.68E-45 |
| **23574** | Phospholipids in small HDL | Lipoprotein subclasses | 0.91 (0.90, 0.93) | 1.14E-34 | 0.91 (0.90, 0.93) | 8.08E-33 |
| **23575** | Cholesterol in small HDL | Lipoprotein subclasses | 0.85 (0.84, 0.86) | 1.05E-115 | 0.88 (0.87, 0.90) | 3.66E-63 |
| **23576** | Cholesteryl esters in small HDL | Lipoprotein subclasses | 0.84 (0.83, 0.86) | 7.55E-123 | 0.88 (0.86, 0.89) | 4.29E-73 |
| **23577** | Free cholesterol in small HDL | Lipoprotein subclasses | 0.88 (0.87, 0.90) | 1.27E-63 | 0.93 (0.91, 0.94) | 3.40E-23 |
| **23578** | Triglycerides in small HDL | Lipoprotein subclasses | 1.07 (1.05, 1.08) | 2.52E-20 | 0.98 (0.96, 0.99) | 2.87E-03 |
| **23579** | Phospholipids to total lipids ratio in chylomicrons and extremely large VLDL | Relative lipoprotein lipid concentrations | 1.08 (1.06, 1.10) | 5.86E-18 | 1.01 (1.00, 1.03) | 1.28E-01 |
| **23580** | Cholesterol to total lipids ratio in chylomicrons and extremely large VLDL | Relative lipoprotein lipid concentrations | 0.97 (0.95, 0.98) | 3.83E-06 | 1.03 (1.01, 1.04) | 1.56E-04 |
| **23581** | Cholesteryl esters to total lipids ratio in chylomicrons and extremely large VLDL | Relative lipoprotein lipid concentrations | 0.96 (0.95, 0.97) | 2.37E-08 | 1.02 (1.01, 1.04) | 3.87E-03 |
| **23582** | Free cholesterol to total lipids ratio in chylomicrons and extremely large VLDL | Relative lipoprotein lipid concentrations | 0.99 (0.97, 1.00) | 4.30E-02 | 1.03 (1.02, 1.05) | 5.21E-05 |
| **23583** | Triglycerides to total lipids ratio in chylomicrons and extremely large VLDL | Relative lipoprotein lipid concentrations | 1.00 (0.98, 1.01) | 7.30E-01 | 0.98 (0.96, 0.99) | 1.71E-03 |
| **23584** | Phospholipids to total lipids ratio in very large VLDL | Relative lipoprotein lipid concentrations | 1.00 (0.99, 1.02) | 6.26E-01 | 1.01 (0.99, 1.03) | 3.80E-01 |
| **23585** | Cholesterol to total lipids ratio in very large VLDL | Relative lipoprotein lipid concentrations | 0.91 (0.90, 0.92) | 4.80E-35 | 1.06 (1.04, 1.07) | 1.87E-11 |
| **23586** | Cholesteryl esters to total lipids ratio in very large VLDL | Relative lipoprotein lipid concentrations | 0.90 (0.89, 0.92) | 5.10E-44 | 1.04 (1.03, 1.06) | 3.03E-07 |
| **23587** | Free cholesterol to total lipids ratio in very large VLDL | Relative lipoprotein lipid concentrations | 0.96 (0.94, 0.97) | 2.65E-09 | 1.09 (1.07, 1.10) | 2.62E-23 |
| **23588** | Triglycerides to total lipids ratio in very large VLDL | Relative lipoprotein lipid concentrations | 1.06 (1.04, 1.07) | 1.96E-11 | 0.93 (0.92, 0.95) | 4.55E-22 |
| **23589** | Phospholipids to total lipids ratio in large VLDL | Relative lipoprotein lipid concentrations | 1.08 (1.05, 1.10) | 4.74E-13 | 1.00 (0.98, 1.02) | 8.27E-01 |
| **23590** | Cholesterol to total lipids ratio in large VLDL | Relative lipoprotein lipid concentrations | 0.96 (0.95, 0.98) | 1.85E-07 | 1.07 (1.06, 1.09) | 8.98E-20 |
| **23591** | Cholesteryl esters to total lipids ratio in large VLDL | Relative lipoprotein lipid concentrations | 0.94 (0.92, 0.95) | 3.70E-21 | 1.08 (1.06, 1.09) | 1.49E-20 |
| **23592** | Free cholesterol to total lipids ratio in large VLDL | Relative lipoprotein lipid concentrations | 1.05 (1.03, 1.07) | 1.11E-09 | 1.05 (1.03, 1.06) | 3.04E-08 |
| **23593** | Triglycerides to total lipids ratio in large VLDL | Relative lipoprotein lipid concentrations | 0.96 (0.95, 0.97) | 2.16E-09 | 0.93 (0.91, 0.94) | 1.41E-36 |
| **23594** | Phospholipids to total lipids ratio in medium VLDL | Relative lipoprotein lipid concentrations | 0.92 (0.91, 0.92) | 7.00E-70 | 1.05 (1.03, 1.06) | 7.39E-09 |
| **23595** | Cholesterol to total lipids ratio in medium VLDL | Relative lipoprotein lipid concentrations | 0.88 (0.87, 0.90) | 2.02E-85 | 1.03 (1.02, 1.05) | 9.32E-06 |
| **23596** | Cholesteryl esters to total lipids ratio in medium VLDL | Relative lipoprotein lipid concentrations | 0.89 (0.88, 0.90) | 3.29E-89 | 1.03 (1.01, 1.04) | 4.50E-04 |
| **23597** | Free cholesterol to total lipids ratio in medium VLDL | Relative lipoprotein lipid concentrations | 0.89 (0.88, 0.90) | 5.26E-73 | 1.04 (1.02, 1.06) | 2.34E-07 |
| **23598** | Triglycerides to total lipids ratio in medium VLDL | Relative lipoprotein lipid concentrations | 1.13 (1.11, 1.14) | 1.11E-60 | 0.95 (0.93, 0.96) | 1.30E-10 |
| **23599** | Phospholipids to total lipids ratio in small VLDL | Relative lipoprotein lipid concentrations | 0.84 (0.83, 0.85) | 5.70E-139 | 0.98 (0.96, 1.00) | 1.06E-02 |
| **23600** | Cholesterol to total lipids ratio in small VLDL | Relative lipoprotein lipid concentrations | 0.89 (0.87, 0.90) | 5.93E-76 | 1.01 (1.00, 1.03) | 5.59E-02 |
| **23601** | Cholesteryl esters to total lipids ratio in small VLDL | Relative lipoprotein lipid concentrations | 0.93 (0.92, 0.94) | 3.34E-29 | 1.04 (1.02, 1.05) | 1.96E-06 |
| **23602** | Free cholesterol to total lipids ratio in small VLDL | Relative lipoprotein lipid concentrations | 0.84 (0.83, 0.85) | 5.49E-148 | 0.98 (0.97, 1.00) | 1.58E-02 |
| **23603** | Triglycerides to total lipids ratio in small VLDL | Relative lipoprotein lipid concentrations | 1.15 (1.13, 1.17) | 1.50E-81 | 0.99 (0.98, 1.01) | 4.30E-01 |
| **23604** | Phospholipids to total lipids ratio in very small VLDL | Relative lipoprotein lipid concentrations | 1.28 (1.26, 1.30) | 1.91E-274 | 1.12 (1.11, 1.14) | 8.59E-56 |
| **23605** | Cholesterol to total lipids ratio in very small VLDL | Relative lipoprotein lipid concentrations | 0.84 (0.83, 0.85) | 9.26E-174 | 0.98 (0.97, 1.00) | 3.14E-02 |
| **23606** | Cholesteryl esters to total lipids ratio in very small VLDL | Relative lipoprotein lipid concentrations | 0.83 (0.82, 0.84) | 3.84E-188 | 0.98 (0.97, 0.99) | 7.45E-03 |
| **23607** | Free cholesterol to total lipids ratio in very small VLDL | Relative lipoprotein lipid concentrations | 0.91 (0.90, 0.92) | 5.92E-49 | 1.01 (1.00, 1.03) | 7.14E-02 |
| **23608** | Triglycerides to total lipids ratio in very small VLDL | Relative lipoprotein lipid concentrations | 1.18 (1.16, 1.20) | 4.74E-121 | 1.00 (0.98, 1.01) | 5.78E-01 |
| **23609** | Phospholipids to total lipids ratio in IDL | Relative lipoprotein lipid concentrations | 1.18 (1.16, 1.19) | 1.68E-116 | 1.10 (1.08, 1.11) | 3.94E-40 |
| **23610** | Cholesterol to total lipids ratio in IDL | Relative lipoprotein lipid concentrations | 0.81 (0.80, 0.82) | 1.85E-283 | 0.92 (0.91, 0.93) | 5.55E-33 |
| **23611** | Cholesteryl esters to total lipids ratio in IDL | Relative lipoprotein lipid concentrations | 0.79 (0.79, 0.80) | 4.26e-319 | 0.90 (0.89, 0.91) | 4.49E-54 |
| **23612** | Free cholesterol to total lipids ratio in IDL | Relative lipoprotein lipid concentrations | 0.91 (0.90, 0.92) | 2.00E-48 | 1.01 (0.99, 1.02) | 3.37E-01 |
| **23613** | Triglycerides to total lipids ratio in IDL | Relative lipoprotein lipid concentrations | 1.25 (1.24, 1.27) | 2.66E-256 | 1.08 (1.06, 1.10) | 4.83E-23 |
| **23614** | Phospholipids to total lipids ratio in large LDL | Relative lipoprotein lipid concentrations | 1.02 (1.00, 1.03) | 1.46E-02 | 1.01 (1.00, 1.02) | 1.36E-01 |
| **23615** | Cholesterol to total lipids ratio in large LDL | Relative lipoprotein lipid concentrations | 0.97 (0.97, 0.97) | 3.70E-78 | 0.98 (0.97, 0.99) | 2.38E-13 |
| **23616** | Cholesteryl esters to total lipids ratio in large LDL | Relative lipoprotein lipid concentrations | 0.97 (0.97, 0.98) | 7.70E-53 | 0.98 (0.97, 0.98) | 8.52E-21 |
| **23617** | Free cholesterol to total lipids ratio in large LDL | Relative lipoprotein lipid concentrations | 0.92 (0.91, 0.92) | 2.38E-149 | 0.99 (0.97, 1.00) | 2.99E-02 |
| **23618** | Triglycerides to total lipids ratio in large LDL | Relative lipoprotein lipid concentrations | 1.28 (1.26, 1.29) | 0.00E+00 | 1.12 (1.10, 1.13) | 4.54E-54 |
| **23619** | Phospholipids to total lipids ratio in medium LDL | Relative lipoprotein lipid concentrations | 1.02 (1.01, 1.04) | 1.60E-03 | 1.01 (0.99, 1.02) | 3.82E-01 |
| **23620** | Cholesterol to total lipids ratio in medium LDL | Relative lipoprotein lipid concentrations | 0.97 (0.97, 0.97) | 1.77E-91 | 0.98 (0.97, 0.98) | 2.26E-18 |
| **23621** | Cholesteryl esters to total lipids ratio in medium LDL | Relative lipoprotein lipid concentrations | 0.97 (0.96, 0.97) | 7.67E-27 | 0.97 (0.97, 0.98) | 2.65E-21 |
| **23622** | Free cholesterol to total lipids ratio in medium LDL | Relative lipoprotein lipid concentrations | 0.91 (0.90, 0.91) | 1.02E-100 | 0.99 (0.97, 1.00) | 3.38E-02 |
| **23623** | Triglycerides to total lipids ratio in medium LDL | Relative lipoprotein lipid concentrations | 1.26 (1.25, 1.28) | 3.33E-305 | 1.12 (1.10, 1.13) | 2.68E-56 |
| **23624** | Phospholipids to total lipids ratio in small LDL | Relative lipoprotein lipid concentrations | 1.11 (1.09, 1.12) | 2.49E-46 | 1.09 (1.08, 1.11) | 1.01E-33 |
| **23625** | Cholesterol to total lipids ratio in small LDL | Relative lipoprotein lipid concentrations | 0.96 (0.96, 0.97) | 1.51E-129 | 0.97 (0.97, 0.98) | 1.31E-31 |
| **23626** | Cholesteryl esters to total lipids ratio in small LDL | Relative lipoprotein lipid concentrations | 0.96 (0.95, 0.97) | 1.47E-45 | 0.97 (0.96, 0.97) | 2.72E-25 |
| **23627** | Free cholesterol to total lipids ratio in small LDL | Relative lipoprotein lipid concentrations | 0.91 (0.90, 0.91) | 5.18E-90 | 0.97 (0.96, 0.99) | 1.21E-05 |
| **23628** | Triglycerides to total lipids ratio in small LDL | Relative lipoprotein lipid concentrations | 1.19 (1.17, 1.20) | 2.39E-145 | 1.05 (1.03, 1.06) | 5.50E-10 |
| **23629** | Phospholipids to total lipids ratio in very large HDL | Relative lipoprotein lipid concentrations | 1.01 (0.99, 1.02) | 2.33E-01 | 1.06 (1.05, 1.08) | 3.18E-14 |
| **23630** | Cholesterol to total lipids ratio in very large HDL | Relative lipoprotein lipid concentrations | 0.93 (0.92, 0.94) | 3.98E-40 | 0.94 (0.93, 0.95) | 3.14E-40 |
| **23631** | Cholesteryl esters to total lipids ratio in very large HDL | Relative lipoprotein lipid concentrations | 0.94 (0.93, 0.95) | 8.61E-58 | 0.98 (0.97, 0.99) | 5.15E-07 |
| **23632** | Free cholesterol to total lipids ratio in very large HDL | Relative lipoprotein lipid concentrations | 1.05 (1.04, 1.07) | 3.14E-13 | 0.93 (0.91, 0.94) | 7.90E-22 |
| **23633** | Triglycerides to total lipids ratio in very large HDL | Relative lipoprotein lipid concentrations | 1.06 (1.05, 1.08) | 9.16E-18 | 0.95 (0.93, 0.96) | 1.67E-13 |
| **23634** | Phospholipids to total lipids ratio in large HDL | Relative lipoprotein lipid concentrations | 1.15 (1.13, 1.17) | 6.33E-78 | 0.98 (0.97, 0.99) | 2.30E-04 |
| **23635** | Cholesterol to total lipids ratio in large HDL | Relative lipoprotein lipid concentrations | 0.93 (0.92, 0.94) | 1.17E-58 | 1.02 (1.01, 1.04) | 7.62E-04 |
| **23636** | Cholesteryl esters to total lipids ratio in large HDL | Relative lipoprotein lipid concentrations | 0.92 (0.91, 0.93) | 4.57E-68 | 1.01 (1.00, 1.02) | 7.94E-02 |
| **23637** | Free cholesterol to total lipids ratio in large HDL | Relative lipoprotein lipid concentrations | 0.96 (0.95, 0.97) | 8.00E-13 | 1.06 (1.05, 1.08) | 5.83E-16 |
| **23638** | Triglycerides to total lipids ratio in large HDL | Relative lipoprotein lipid concentrations | 1.10 (1.09, 1.12) | 1.46E-40 | 0.98 (0.97, 1.00) | 2.19E-02 |
| **23639** | Phospholipids to total lipids ratio in medium HDL | Relative lipoprotein lipid concentrations | 1.19 (1.18, 1.21) | 3.27E-134 | 1.04 (1.02, 1.05) | 3.47E-06 |
| **23640** | Cholesterol to total lipids ratio in medium HDL | Relative lipoprotein lipid concentrations | 0.93 (0.93, 0.94) | 1.18E-128 | 0.97 (0.96, 0.99) | 1.91E-05 |
| **23641** | Cholesteryl esters to total lipids ratio in medium HDL | Relative lipoprotein lipid concentrations | 0.93 (0.92, 0.93) | 1.04E-100 | 0.97 (0.96, 0.98) | 1.56E-06 |
| **23642** | Free cholesterol to total lipids ratio in medium HDL | Relative lipoprotein lipid concentrations | 0.87 (0.86, 0.88) | 6.09E-141 | 1.00 (0.98, 1.02) | 9.33E-01 |
| **23643** | Triglycerides to total lipids ratio in medium HDL | Relative lipoprotein lipid concentrations | 1.13 (1.11, 1.14) | 9.68E-55 | 1.02 (1.00, 1.03) | 1.74E-02 |
| **23644** | Phospholipids to total lipids ratio in small HDL | Relative lipoprotein lipid concentrations | 1.13 (1.11, 1.14) | 1.30E-61 | 1.09 (1.07, 1.10) | 1.91E-28 |
| **23645** | Cholesterol to total lipids ratio in small HDL | Relative lipoprotein lipid concentrations | 0.85 (0.84, 0.86) | 2.18E-129 | 0.93 (0.92, 0.95) | 1.08E-23 |
| **23646** | Cholesteryl esters to total lipids ratio in small HDL | Relative lipoprotein lipid concentrations | 0.88 (0.87, 0.89) | 1.01E-170 | 0.92 (0.91, 0.93) | 4.36E-40 |
| **23647** | Free cholesterol to total lipids ratio in small HDL | Relative lipoprotein lipid concentrations | 0.94 (0.93, 0.96) | 5.24E-15 | 1.08 (1.06, 1.09) | 2.33E-22 |
| **23648** | Triglycerides to total lipids ratio in small HDL | Relative lipoprotein lipid concentrations | 1.15 (1.13, 1.17) | 3.32E-76 | 1.03 (1.01, 1.04) | 9.45E-04 |

Notes: HR, hazard ratio; CI, confidence interval; VLDL, very low-density lipoprotein; LDL, low-density lipoprotein; IDL, intermediate density lipoprotein; HDL, high-density lipoprotein.

^a^ Model 1 was adjusted for chronological age and sex.

^b^ Model 2 was further adjusted for ethnicity, education level, Townsend deprivation index, alcohol intake frequency, smoking status, regular exercise, healthy diet, body mass index, cholesterol-lowering medication, anti-hypertensive medication, anti-diabetes medication, and some prevalent diseases (i.e., cancer, cardiovascular disease, hypertension, diabetes mellitus, and chronic obstructive pulmonary disease) at baseline based on Model 1.

Table S3. Selected variables in our MetaboAgeMort model through LASSO Cox regression.

| **Field ID** | **Title** | **Group** | **Coefficient** |
| --- | --- | --- | --- |
| - | Chronological age | - | 0.095515171 |
| 23431 | Average diameter for VLDL particles | Lipoprotein particle sizes | -0.069924780 |
| 23449 | Linoleic acid | Fatty acids | -0.201116669 |
| 23451 | Ratio of omega-3 fatty acids to total fatty acids | Fatty acids | -0.246749381 |
| 23454 | Ratio of monounsaturated fatty acids to total fatty acids | Fatty acids | 0.023823060 |
| 23456 | Ratio of linoleic acid to total fatty acids | Fatty acids | -0.078028580 |
| 23460 | Alanine | Amino acids | 0.002923644 |
| 23463 | Histidine | Amino acids | -0.017531653 |
| 23466 | Leucine | Amino acids | -0.013850371 |
| 23467 | Valine | Amino acids | -0.099048925 |
| 23468 | Phenylalanine | Amino acids | 0.029094661 |
| 23469 | Tyrosine | Amino acids | 0.039062967 |
| 23470 | Glucose | Glycolysis related metabolites | 0.081136089 |
| 23472 | Pyruvate | Glycolysis related metabolites | 0.000913441 |
| 23473 | Citrate | Glycolysis related metabolites | -0.027884519 |
| 23474 | 3-Hydroxybutyrate | Ketone bodies | 0.056930816 |
| 23475 | Acetate | Ketone bodies | 0.006330656 |
| 23476 | Acetoacetate | Ketone bodies | 0.002026289 |
| 23477 | Acetone | Ketone bodies | 0.024260197 |
| 23478 | Creatinine | Fluid balance | 0.090540901 |
| 23479 | Albumin | Fluid balance | -0.066261250 |
| 23480 | Glycoprotein acetyls | Inflammation | 0.231770781 |
| 23494 | Triglycerides in very large VLDL | Lipoprotein subclasses | -0.000343691 |
| 23556 | Free cholesterol in very large HDL | Lipoprotein subclasses | 0.007832035 |
| 23573 | Total lipids in small HDL | Lipoprotein subclasses | -0.020418898 |
| 23576 | Cholesteryl esters in small HDL | Lipoprotein subclasses | -0.008749055 |
| 23593 | Triglycerides to total lipids ratio in large VLDL | Relative lipoprotein lipid concentrations | -0.024607925 |
| 23604 | Phospholipids to total lipids ratio in very small VLDL | Relative lipoprotein lipid concentrations | 0.054338149 |
| 23609 | Phospholipids to total lipids ratio in IDL | Relative lipoprotein lipid concentrations | 0.001716527 |
| 23611 | Cholesteryl esters to total lipids ratio in IDL | Relative lipoprotein lipid concentrations | -0.004107152 |
| 23618 | Triglycerides to total lipids ratio in large LDL | Relative lipoprotein lipid concentrations | 0.104710479 |
| 23623 | Triglycerides to total lipids ratio in medium LDL | Relative lipoprotein lipid concentrations | 0.006877809 |
| 23624 | Phospholipids to total lipids ratio in small LDL | Relative lipoprotein lipid concentrations | 0.006447548 |
| 23627 | Free cholesterol to total lipids ratio in small LDL | Relative lipoprotein lipid concentrations | 0.041369487 |
| 23631 | Cholesteryl esters to total lipids ratio in very large HDL | Relative lipoprotein lipid concentrations | 0.003232659 |
| 23647 | Free cholesterol to total lipids ratio in small HDL | Relative lipoprotein lipid concentrations | 0.053282046 |

Notes: The parameter λ of 0.0009694623 (which represented a one standard deviation increase over the λ with minimum mean-squared error) was selected using ten-fold cross-validation to develop a sparse MetaboAgeMort estimator. LASSO, least absolute shrinkage and selection operator; VLDL, very low-density lipoprotein; LDL, low-density lipoprotein; IDL, intermediate density lipoprotein; HDL, high-density lipoprotein.

Table S4. The MetaboAgeMort measures and Gompertz coefficients.

| **Model** | **Field ID** | **Parameter** | **Mean** | **SD** | **Coefficient** | **Lower 95% CI** | **Upper 95% CI** |
| --- | --- | --- | --- | --- | --- | --- | --- |
| **S~** Chronological age | - | Shape (γ_0_) | **-** | - | 0.122739 | 0.118483 | 0.126995 |
|  | - | Rate (exp (β_0_)) | **-** | - | 4.33E-06 | 3.64E-06 | 5.14E-06 |
|  | - | Chronological age (β_1_) | **-** | - | 0.105645 | 0.102974 | 0.108316 |
| **S~** Chronological age+35 Metabolic biomarkers | - | Shape (γ_1_) | - | - | 0.135368 | 0.131100 | 0.139635 |
|  | - | Rate | - | - | 5.86E-06 | 4.92E-06 | 6.98E-06 |
|  | - | Chronological age | - | - | 0.096630 | 0.093885 | 0.099375 |
|  | 23431 | Average diameter for VLDL particles | 3.68120 | 0.03022 | -0.042853 | -0.106821 | 0.021116 |
|  | 23449 | Linoleic acid | 1.48838 | 0.15301 | -0.095444 | -0.199349 | 0.008462 |
|  | 23451 | Ratio of omega-3 fatty acids to total fatty acids | 1.64190 | 0.27398 | -0.270218 | -0.297652 | -0.242784 |
|  | 23454 | Ratio of monounsaturated fatty acids to total fatty acids | 3.20821 | 0.10313 | 0.055855 | 0.000699 | 0.111012 |
|  | 23456 | Ratio of linoleic acid to total fatty acids | 3.39041 | 0.11816 | -0.186557 | -0.265523 | -0.107592 |
|  | 23460 | Alanine | 0.25868 | 0.05941 | 0.019525 | 0.000596 | 0.038455 |
|  | 23463 | Histidine | 0.06348 | 0.01039 | -0.016779 | -0.035489 | 0.001931 |
|  | 23466 | Leucine | 0.09944 | 0.02580 | -0.071978 | -0.112484 | -0.031471 |
|  | 23467 | Valine | 0.19106 | 0.03539 | -0.079661 | -0.121967 | -0.037354 |
|  | 23468 | Phenylalanine | 0.04622 | 0.01050 | 0.037781 | 0.018821 | 0.056741 |
|  | 23469 | Tyrosine | 0.06116 | 0.01355 | 0.065474 | 0.044342 | 0.086606 |
|  | 23470 | Glucose | 1.52386 | 0.19852 | 0.082497 | 0.066748 | 0.098245 |
|  | 23472 | Pyruvate | 0.07761 | 0.02643 | 0.013142 | -0.005832 | 0.032116 |
|  | 23473 | Citrate | 0.06336 | 0.01220 | -0.057192 | -0.076252 | -0.038131 |
|  | 23474 | 3-Hydroxybutyrate | 0.05744 | 0.05116 | 0.060319 | 0.025891 | 0.094747 |
|  | 23475 | Acetate | 0.01584 | 0.01034 | 0.015139 | 0.003798 | 0.026480 |
|  | 23476 | Acetoacetate | 0.01292 | 0.01159 | 0.000664 | -0.028408 | 0.029736 |
|  | 23477 | Acetone | 0.01408 | 0.00518 | 0.031093 | 0.004937 | 0.057248 |
|  | 23478 | Creatinine | 0.06529 | 0.01309 | 0.094777 | 0.085277 | 0.104277 |
|  | 23479 | Albumin | 3.69476 | 0.08339 | -0.066562 | -0.085664 | -0.047460 |
|  | 23480 | Glycoprotein acetyls | 0.59394 | 0.06473 | 0.224532 | 0.202035 | 0.247029 |
|  | 23494 | Triglycerides in very large VLDL | 0.10780 | 0.07031 | -0.114985 | -0.197763 | -0.032208 |
|  | 23556 | Free cholesterol in very large HDL | 0.02256 | 0.00680 | 0.002427 | -0.058677 | 0.063531 |
|  | 23573 | Total lipids in small HDL | 0.77232 | 0.07278 | -0.142922 | -0.274986 | -0.010858 |
|  | 23576 | Cholesteryl esters in small HDL | 0.28637 | 0.03559 | 0.031527 | -0.089215 | 0.152269 |
|  | 23593 | Triglycerides to total lipids ratio in large VLDL | 3.93890 | 0.10708 | -0.022566 | -0.042291 | -0.002840 |
|  | 23604 | Phospholipids to total lipids ratio in very small VLDL | 3.40963 | 0.02908 | 0.028942 | -0.008534 | 0.066417 |
|  | 23609 | Phospholipids to total lipids ratio in IDL | 3.21262 | 0.03628 | 0.067321 | 0.023637 | 0.111005 |
|  | 23611 | Cholesteryl esters to total lipids ratio in IDL | 3.92932 | 0.04859 | 0.057180 | -0.001978 | 0.116338 |
|  | 23618 | Triglycerides to total lipids ratio in large LDL | 1.97219 | 0.21074 | 0.241849 | 0.119754 | 0.363945 |
|  | 23623 | Triglycerides to total lipids ratio in medium LDL | 1.84171 | 0.20902 | -0.008525 | -0.106210 | 0.089159 |
|  | 23624 | Phospholipids to total lipids ratio in small LDL | 3.47050 | 0.05352 | -0.019941 | -0.055092 | 0.015210 |
|  | 23627 | Free cholesterol to total lipids ratio in small LDL | 2.89992 | 0.12831 | 0.109706 | 0.064974 | 0.154437 |
|  | 23631 | Cholesteryl esters to total lipids ratio in very large HDL | 3.57643 | 0.13347 | 0.026571 | 0.002915 | 0.050227 |
|  | 23647 | Free cholesterol to total lipids ratio in small HDL | 2.38912 | 0.04640 | 0.055281 | 0.018502 | 0.092059 |

Notes: Two proportional hazards regression models based on the parametric Gompertz distribution were fitted: one used only chronological age as a predictor, and the other used 36 variables selected through LASSO regression as predictors. SD, standard deviation; CI, confidence interval; VLDL, very low-density lipoprotein; LDL, low-density lipoprotein; IDL, intermediate density lipoprotein; HDL, high-density lipoprotein.

| **Mortality category** | **No. of events/No. participants** | **HR (95% CI)** | **P value** |
| --- | --- | --- | --- |
| Training set | | | |
| All-cause mortality ^a^ |  |  |  |
| Full sample | 14,303/167,506 | 1.07 (1.07, 1.08) | <0.001 |
| Those with ≥ 5 years of survival ^b^ | 11,573/164,500 | 1.07 (1.06, 1.07) | <0.001 |
| Cause-specific mortality ^c^ |  |  |  |
| Cancer | 6,976/167,506 | 1.06 (1.05, 1.06) | <0.001 |
| Cardiovascular disease | 2,979/167,506 | 1.08 (1.08, 1.09) | <0.001 |
| Respiratory disease | 1,054/167,506 | 1.11 (1.10, 1.12) | <0.001 |
| Digestive disease | 537/167,506 | 1.13 (1.12, 1.15) | <0.001 |
| Neurodegenerative disease | 1,051/167,506 | 1.03 (1.02, 1.04) | <0.001 |
| Other causes ^d^ | 1,706/167,506 | 1.08 (1.07, 1.09) | <0.001 |
| Testing set | | | |
| All-cause mortality ^a^ |  |  |  |
| Full sample | 6,144/71,785 | 1.07 (1.06, 1.08) | <0.001 |
| Those with ≥ 5 years of survival ^b^ | 4,966/70,493 | 1.06 (1.05, 1.07) | <0.001 |
| Cause-specific mortality ^c^ |  |  |  |
| Cancer | 3,007/71,785 | 1.06 (1.05, 1.07) | <0.001 |
| Cardiovascular disease | 1,299/71,785 | 1.08 (1.07, 1.10) | <0.001 |
| Respiratory disease | 416/71,785 | 1.08 (1.06, 1.10) | <0.001 |
| Digestive disease | 201/71,785 | 1.12 (1.09, 1.14) | <0.001 |
| Neurodegenerative disease | 482/71,785 | 1.03 (1.01, 1.04) | 0.004 |
| Other causes ^d^ | 739/71,785 | 1.09 (1.07, 1.10) | <0.001 |

Table S5. Associations of MetaboAgeMort with all-cause mortality and cause-specific mortality.

Notes: HR, hazard ratio; CI, confidence interval.

^a^ The association of MetaboAgeMort with all-cause mortality was estimated using Cox proportional hazard regression model with adjustment for chronological age, sex, ethnicity, education level, Townsend deprivation index, alcohol intake frequency, smoking status, regular exercise, healthy diet, body mass index, cholesterol-lowering medication, anti-hypertensive medication, anti-diabetes medication, and some prevalent diseases (i.e., cancer, cardiovascular disease, hypertension, diabetes mellitus, and chronic obstructive pulmonary disease) at baseline.

^b^ To reduce the influence of end-of-life metabolomic status, we ran a model that excluded participants who died within 5 years of follow-up.

^c^ The associations of the MetaboAgeMort with cause-specific mortality were estimated Fine and Gray’s competing risk models with adjustment for chronological age, sex, ethnicity, education level, Townsend deprivation index, alcohol intake frequency, smoking status, regular exercise, healthy diet, and body mass index.

^d^ Other causes mortality refers to death from causes other than cancer, cardiovascular disease, respiratory disease, digestive disease, and neurodegenerative disease.

Table S6. Associations of MetaboAgeMort with all-cause mortality in population subgroups in the testing set.

| **Subgroups** | | **No. of events/No. participants** | **HR (95% CI)** | **P *interaction*** |
| --- | --- | --- | --- | --- |
| Chronological age | < 60 years | 1,645/40,356 | 1.08 (1.07, 1.09) | 0.055 |
|  | ≥ 60 years | 4,499/31,429 | 1.07 (1.06, 1.07) |  |
| Sex | Female | 2,510/37,870 | 1.07 (1.06, 1.08) | 0.857 |
|  | Male | 3,634/33,915 | 1.07 (1.06, 1.08) |  |
| Ethnicity | Non-British White | 164/3,261 | 1.07 (1.04, 1.10) | 0.950 |
|  | British White | 5,980/68,524 | 1.07 (1.06, 1.08) |  |
| Educational level | High | 1,449/23,183 | 1.08 (1.07, 1.09) | 0.043 |
|  | Intermediate | 1,718/23,308 | 1.07 (1.06, 1.08) |  |
|  | Low | 2,977/25,294 | 1.06 (1.06, 1.07) |  |
| Smoking status | Never smoker | 2,410/39,273 | 1.07 (1.06, 1.07) | 0.620 |
|  | Previous smoker | 2,582/24,967 | 1.07 (1.06, 1.08) |  |
|  | Current smoker | 1,152/7,545 | 1.07 (1.06, 1.08) |  |
| Alcohol intake frequency | Never or special occasions | 1,544/13,797 | 1.07 (1.06, 1.08) | 0.004 |
|  | 1 to 3 times per month | 638/8,164 | 1.07 (1.05, 1.09) |  |
|  | 1 to 4 times per week | 2,662/35,572 | 1.06 (1.05, 1.07) |  |
|  | Daily or almost daily | 1,300/14,252 | 1.08 (1.07, 1.10) |  |
| Regular exercise | No | 3,151/32,759 | 1.07 (1.06, 1.08) | 0.682 |
|  | Yes | 2,993/39,026 | 1.07 (1.06, 1.08) |  |
| Healthy diet | No | 1,407/14,764 | 1.07 (1.06, 1.08) | 0.075 |
|  | Yes | 4,737/57,021 | 1.07 (1.06, 1.08) |  |
| Body mass index ^a^ | Underweight | 47/302 | 1.01 (0.96, 1.05) | 0.014 |
|  | Normal | 1,607/22,639 | 1.08 (1.07, 1.09) |  |
|  | Overweight | 2,516/31,059 | 1.06 (1.05, 1.07) |  |
|  | Obese | 1,974/17,785 | 1.06 (1.05, 1.07) |  |

Notes: The Cox proportional hazard regression model was adjusted for chronological age, sex, ethnicity, education level, Townsend deprivation index, alcohol intake frequency, smoking status, regular exercise, healthy diet, body mass index, cholesterol-lowering medication, anti-hypertensive medication, anti-diabetes medication, and some prevalent diseases (i.e., cancer, cardiovascular disease, hypertension, diabetes mellitus, and chronic obstructive pulmonary disease) at baseline. In each stratified analysis, the stratification variable (except chronological age) was excluded in the models. The interactions were all not significant after the Bonferroni correction. HR, hazard ratio; CI, confidence interval.

^a^ Underweight was defined as BMI < 18.5 kg/m^2^, normal was defined as 18.5 kg/m^2^ ≤ BMI < 25.0 kg/m^2^, overweight was defined as 25.0 kg/m^2^ ≤ BMI < 30.0 kg/m^2^, and obese was defined as BMI ≥ 30 kg/m^2^.

Table S7. Associations of MetaboAgeMort with all-cause mortality in diseases-free participants.

| **Dataset** | **No. of events/No. participants** | **HR (95% CI)** | **P value** |
| --- | --- | --- | --- |
| Training set | 2,507/61,139 | 1.07 (1.06, 1.08) | <0.001 |
| Testing set | 1,058/26,048 | 1.06 (1.05, 1.08) | <0.001 |

Notes: Diseases-free participants were defined as those who were free of several common prevalent diseases (i.e., cancer, cardiovascular disease, hypertension, diabetes mellitus, chronic pulmonary disease, and depression) at baseline. The Cox proportional hazard regression model was adjusted for chronological age, sex, ethnicity, education level, Townsend deprivation index, alcohol intake frequency, smoking status, regular exercise, healthy diet, and body mass index. HR, hazard ratio; CI, confidence interval.

Table S8. Associations of the MetaboAgeMort Acceleration with all-cause mortality and cause-specific mortality.

| **Mortality category** | **No. of events/No. participants** | **1-year increment** | | **Highest quartile** ^a^ | |
| --- | --- | --- | --- | --- | --- |
|  |  | **HR (95% CI)** | **P value** | **HR (95% CI)** | **P value** |
| Training set | | | | | |
| All-cause mortality ^b^ |  |  |  |  |  |
| Full sample | 14,303/167,506 | 1.07 (1.07, 1.08) | <0.001 | 2.10 (1.99, 2.22) | <0.001 |
| Those with ≥ 5 years of survival ^c^ | 11,573/164,500 | 1.07 (1.06, 1.07) | <0.001 | 1.95 (1.83, 2.07) | <0.001 |
| Cause-specific mortality ^d^ |  |  |  |  |  |
| Cancer | 6,976/167,506 | 1.06 (1.05, 1.06) | <0.001 | 1.87 (1.73, 2.01) | <0.001 |
| Cardiovascular disease | 2,979/167,506 | 1.08 (1.08, 1.09) | <0.001 | 2.73 (2.40, 3.11) | <0.001 |
| Respiratory disease | 1,054/167,506 | 1.11 (1.10, 1.12) | <0.001 | 5.29 (4.07, 6.88) | <0.001 |
| Digestive disease | 537/167,506 | 1.13 (1.12, 1.15) | <0.001 | 5.90 (3.98, 8.75) | <0.001 |
| Neurodegenerative disease | 1,051/167,506 | 1.03 (1.02, 1.04) | <0.001 | 1.54 (1.28, 1.85) | <0.001 |
| Other causes ^e^ | 1,706/167,506 | 1.08 (1.07, 1.09) | <0.001 | 2.40 (2.04, 2.83) | <0.001 |
| Testing set | | | | | |
| All-cause mortality ^b^ |  |  |  |  |  |
| Full sample | 6,144/71,785 | 1.07 (1.06, 1.08) | <0.001 | 2.01 (1.85, 2.19) | <0.001 |
| Those with ≥ 5 years of survival ^c^ | 4,966/70,493 | 1.06 (1.05, 1.07) | <0.001 | 1.84 (1.68, 2.02) | <0.001 |
| Cause-specific mortality ^d^ |  |  |  |  |  |
| Cancer | 3,007/71,785 | 1.06 (1.05, 1.07) | <0.001 | 1.95 (1.74, 2.19) | <0.001 |
| Cardiovascular disease | 1,299/71,785 | 1.08 (1.07, 1.10) | <0.001 | 2.25 (1.87, 2.71) | <0.001 |
| Respiratory disease | 416/71,785 | 1.08 (1.06, 1.10) | <0.001 | 4.07 (2.78, 5.95) | <0.001 |
| Digestive disease | 201/71,785 | 1.12 (1.09, 1.14) | <0.001 | 4.17 (2.40, 7.24) | <0.001 |
| Neurodegenerative disease | 482/71,785 | 1.03 (1.01, 1.04) | 0.004 | 1.37 (1.04, 1.80) | 0.026 |
| Other causes ^e^ | 739/71,785 | 1.09 (1.07, 1.10) | <0.001 | 2.32 (1.83, 2.95) | <0.001 |

Notes: HR, hazard ratio; CI, confidence interval.

^a^ The lowest quartile of the MetaboAgeMort Acceleration (Q1) was set as the reference.

^b^ The association of the MetaboAgeMort Acceleration with all-cause mortality was estimated using Cox proportional hazard regression model with adjustment for chronological age, sex, ethnicity, education level, Townsend deprivation index, alcohol intake frequency, smoking status, regular exercise, healthy diet, body mass index, cholesterol-lowering medication, anti-hypertensive medication, anti-diabetes medication, and some prevalent diseases (i.e., cancer, cardiovascular disease, hypertension, diabetes mellitus, and chronic obstructive pulmonary disease) at baseline.

^c^ To reduce the influence of end-of-life metabolomic status, we ran a model that excluded participants who died within 5 years of follow-up.

^d^ The associations of the MetaboAgeMort Acceleration with cause-specific mortality were estimated Fine and Gray’s competing risk models with adjustment for chronological age, sex, ethnicity, education level, Townsend deprivation index, alcohol intake frequency, smoking status, regular exercise, healthy diet, and body mass index.

^e^ Other causes mortality refers to death from causes other than cancer, cardiovascular disease, respiratory disease, digestive disease, and neurodegenerative disease.

Table S9. Associations of MetaboAgeMort with some common disease incidence.

| **Disease category** | **No. of events/No. participants** | **MetaboAgeMort** | |
| --- | --- | --- | --- |
|  |  | **HR (95% CI)** | **P value** |
| Training set | | | |
| Cancer | 16,761/153,306 | 1.02 (1.02, 1.02) | <0.001 |
| Cardiovascular disease | 14,991/156,279 | 1.03 (1.03, 1.04) | <0.001 |
| Dementia | 3,007/167,434 | 1.04 (1.03, 1.05) | <0.001 |
| Liver disease | 4,976/166,726 | 1.06 (1.05, 1.07) | <0.001 |
| Respiratory disease | 7,799/163,836 | 1.06 (1.06, 1.07) | <0.001 |
| Chronic kidney disease | 8,173/166,772 | 1.10 (1.10, 1.11) | <0.001 |
| Hypertension | 6,477/74,170 | 1.05 (1.04, 1.05) | <0.001 |
| Type 2 diabetes mellitus | 7,406/159,008 | 1.07 (1.06, 1.07) | <0.001 |
| Osteoarthritis | 24,853/148,793 | 1.01 (1.01, 1.01) | <0.001 |
| Eye disease | 20,812/161,643 | 1.02 (1.01, 1.02) | <0.001 |
| Depression | 6,505/157,037 | 1.03 (1.03, 1.04) | <0.001 |
| Anxiety | 8,279/164,398 | 1.03 (1.02, 1.03) | <0.001 |
| Testing set | | | |
| Cancer | 7,274/65,790 | 1.02 (1.02, 1.03) | <0.001 |
| Cardiovascular disease | 6,364/66,998 | 1.03 (1.03, 1.04) | <0.001 |
| Dementia | 1,321/71,758 | 1.04 (1.03, 1.05) | <0.001 |
| Liver disease | 2,183/71,438 | 1.06 (1.05, 1.07) | <0.001 |
| Respiratory disease | 3,221/70,127 | 1.07 (1.06, 1.07) | <0.001 |
| Chronic kidney disease | 3,596/71,483 | 1.09 (1.09, 1.10) | <0.001 |
| Hypertension | 2,846/31,558 | 1.05 (1.04, 1.06) | <0.001 |
| Type 2 diabetes mellitus | 3,308/68,038 | 1.07 (1.06, 1.08) | <0.001 |
| Osteoarthritis | 10,780/63,835 | 1.00 (1.00, 1.01) | 0.069 |
| Eye disease | 8,965/69,225 | 1.02 (1.01, 1.02) | <0.001 |
| Depression | 2,745/67,254 | 1.03 (1.02, 1.03) | <0.001 |
| Anxiety | 3,504/70,458 | 1.02 (1.01, 1.02) | <0.001 |

Notes: The Fine and Gray’s competing risk models were adjusted for chronological age, sex, ethnicity, education level, Townsend deprivation index, alcohol intake frequency, smoking status, regular exercise, healthy diet, and body mass index. The participants with the specific diagnosis before or at the time of recruitment were excluded from the models. HR, hazard ratio; CI, confidence interval.

Table S10. Associations of the MetaboAgeMort Acceleration with some common disease incidence in training set.

| **Disease category** | **No. of events/No. participants** | **1-year increment** | | **Highest quartile ^a^** | |
| --- | --- | --- | --- | --- | --- |
|  |  | **HR (95 %CI)** | **P value** | **HR (95% CI)** | **P value** |
| Cancer | 16,761/153,306 | 1.02 (1.02, 1.02) | <0.001 | 1.26 (1.20, 1.32) | <0.001 |
| Cardiovascular disease | 14,991/156,279 | 1.03 (1.03, 1.04) | <0.001 | 1.47 (1.39, 1.54) | <0.001 |
| Dementia | 3,007/167,434 | 1.04 (1.03, 1.05) | <0.001 | 1.60 (1.43, 1.79) | <0.001 |
| Liver disease | 4,976/166,726 | 1.06 (1.05, 1.07) | <0.001 | 2.01 (1.83, 2.20) | <0.001 |
| Respiratory disease | 7,799/163,836 | 1.06 (1.06, 1.07) | <0.001 | 2.36 (2.19, 2.55) | <0.001 |
| Chronic kidney disease | 8,173/166,772 | 1.10 (1.10, 1.11) | <0.001 | 3.40 (3.14, 3.68) | <0.001 |
| Hypertension | 6,477/74,170 | 1.05 (1.04, 1.05) | <0.001 | 1.70 (1.58, 1.84) | <0.001 |
| Type 2 diabetes mellitus | 7,406/159,008 | 1.07 (1.06, 1.07) | <0.001 | 2.72 (2.50, 2.96) | <0.001 |
| Osteoarthritis | 24,853/148,793 | 1.01 (1.01, 1.01) | <0.001 | 1.13 (1.09, 1.17) | <0.001 |
| Eye disease | 20,812/161,643 | 1.02 (1.01, 1.02) | <0.001 | 1.46 (1.35, 1.58) | <0.001 |
| Depression | 6,505/157,037 | 1.03 (1.03, 1.04) | <0.001 | 1.35 (1.26, 1.44) | <0.001 |
| Anxiety | 8,279/164,398 | 1.03 (1.02, 1.03) | <0.001 | 1.19 (1.15, 1.25) | <0.001 |

Notes: The Fine and Gray’s competing risk models were adjusted for chronological age, sex, ethnicity, education level, Townsend deprivation index, alcohol intake frequency, smoking status, regular exercise, healthy diet, and body mass index. The participants with the specific diagnosis before or at the time of recruitment were excluded from the models. HR, hazard ratio; CI, confidence interval.

^a^ The lowest quartile of the MetaboAgeMort acceleration (Q1) was set as the reference.

Table S11. Associations of the MetaboAgeMort Acceleration with some common disease incidence in the testing set.

| **Disease category** | **No. of events/No. participants** | **1-year increment** | | **Highest quartile ^a^** | |
| --- | --- | --- | --- | --- | --- |
|  |  | **HR (95% CI)** | **P value** | **HR (95% CI)** | **P value** |
| Cancer | 7,274/65,790 | 1.02 (1.02, 1.03) | <0.001 | 1.27 (1.18, 1.36) | <0.001 |
| Cardiovascular disease | 6,364/66,998 | 1.03 (1.03, 1.04) | <0.001 | 1.46 (1.35, 1.57) | <0.001 |
| Dementia | 1,321/71,758 | 1.04 (1.03, 1.05) | <0.001 | 1.51 (1.27, 1.79) | 0.004 |
| Liver disease | 2,183/71,438 | 1.06 (1.05, 1.07) | <0.001 | 2.05 (1.77, 2.36) | <0.001 |
| Respiratory disease | 3,221/70,127 | 1.07 (1.06, 1.07) | <0.001 | 2.46 (2.18, 2.78) | <0.001 |
| Chronic kidney disease | 3,596/71,483 | 1.09 (1.09, 1.10) | <0.001 | 3.21 (2.86, 3.61) | <0.001 |
| Hypertension | 2,846/31,558 | 1.05 (1.04, 1.06) | <0.001 | 1.67 (1.49, 1.87) | <0.001 |
| Type 2 diabetes mellitus | 3,308/68,038 | 1.07 (1.06, 1.08) | <0.001 | 3.03 (2.64, 3.46) | <0.001 |
| Osteoarthritis | 10,780/63,835 | 1.00 (1.00, 1.01) | 0.069 | 1.09 (1.03, 1.16) | 0.004 |
| Eye disease | 8,965/69,225 | 1.02 (1.01, 1.02) | <0.001 | 1.25 (1.18, 1.33) | <0.001 |
| Depression | 2,745/67,254 | 1.03 (1.02, 1.03) | <0.001 | 1.32 (1.17, 1.48) | <0.001 |
| Anxiety | 3,504/70,458 | 1.02 (1.01, 1.02) | <0.001 | 1.27 (1.14, 1.41) | <0.001 |

Notes: The Fine and Gray’s competing risk models were was adjusted for chronological age, sex, ethnicity, education level, Townsend deprivation index, alcohol intake frequency, smoking status, regular exercise, healthy diet, and body mass index. The participants with the specific diagnosis before or at the time of recruitment were excluded from the models. HR, hazard ratio; CI, confidence interval.

^a^ The lowest quartile of the MetaboAgeMort acceleration (Q1) was set as the reference.

Table S12. Results of the discrimination and reclassification analyses for 10-years all-cause mortality comparing the conventional risk factor score with the combined model.

| **Group** | **C-statistic** | | | **IDI** |
| --- | --- | --- | --- | --- |
|  | **Conventional risk factors** | **Combined model** | **P value** |  |
| Training set | | | | |
| All | 0.7556 (0.7499, 0.7613) | 0.7713 (0.7656, 0.7770) | <0.001 | 0.019 (0.005, 0.021) |
| > 60 | 0.6944 (0.6867, 0.7021) | 0.7153 (0.7076, 0.7231) | <0.001 | 0.021 (0.004, 0.023) |
| Testing set | | | | |
| All | 0.7647 (0.7561, 0.7732) | 0.7815 (0.7730, 0.7900) | <0.001 | 0.018 (0.004, 0.021) |
| > 60 | 0.7011 (0.6894, 0.7128) | 0.7215 (0.7098, 0.7332) | <0.001 | 0.018 (0.003, 0.024) |

Notes: The conventional risk factors included chronological age, sex, body mass index, systolic blood pressure, total cholesterol, high-density lipoprotein cholesterol, triglycerides, creatinine, smoking status, alcohol intake frequency, and prevalent diabetes, cardiovascular disease, and cancer. The combined model included conventional risk factors and the MetaboAgeMort. IDI, integrated discrimination improvement.

Table S13. Associations of several multi-metabolites scores with health-related outcomes in the testing set.

| **Health-related outcomes** | **MetaboHealth** | | **MetaboAge_EN** | | **MetaboAge_LM** | |
| --- | --- | --- | --- | --- | --- | --- |
|  | **HR (95% CI)** | **P value** | **HR (95% CI)** | **P value** | **HR (95% CI)** | **P value** |
| **All-cause mortality** | 1.96 (1.81, 2.13) | <0.001 | 1.16 (1.08, 1.24) | <0.001 | 0.98 (0.91, 1.05) | 0.584 |
| **Cause-specific mortality** |  |  |  |  |  |  |
| Cancer | 1.85 (1.65, 2.08) | <0.001 | 1.08 (0.97, 1.19) | 0.150 | 0.98 (0.89, 1.08) | 0.690 |
| Cardiovascular disease | 2.23 (1.87, 2.66) | <0.001 | 1.35 (1.16, 1.57) | <0.001 | 1.05 (0.89, 1.23) | 0.560 |
| Respiratory disease | 3.62 (2.51, 5.20) | <0.001 | 1.29 (0.99, 1.67) | 0.057 | 0.73 (0.56, 0.96) | 0.024 |
| Digestive disease | 4.03 (2.37, 6.87) | <0.001 | 1.10 (0.78, 1.56) | 0.590 | 1.00 (0.68, 1.48) | 1.000 |
| Neurodegenerative disease | 1.19 (0.91, 1.55) | 0.210 | 0.98 (0.76, 1.26) | 0.870 | 1.17 (0.90, 1.54) | 0.250 |
| Other causes | 2.36 (1.87, 2.97) | <0.001 | 1.30 (1.05, 1.59) | 0.014 | 0.95 (0.77, 1.17) | 0.640 |
| **Common diseases** |  |  |  |  |  |  |
| Cancer | 1.30 (1.21, 1.40) | <0.001 | 0.97 (0.91, 1.03) | 0.340 | 1.06 (0.99, 1.13) | 0.072 |
| Cardiovascular disease | 1.37 (1.27, 1.47) | <0.001 | 1.15 (1.07, 1.23) | <0.001 | 1.05 (0.98, 1.13) | 0.140 |
| Dementia | 1.35 (1.14, 1.60) | <0.001 | 1.08 (0.92, 1.26) | 0.340 | 0.99 (0.85, 1.16) | 0.930 |
| Liver disease | 1.56 (1.36, 1.79) | <0.001 | 0.96 (0.85, 1.08) | 0.490 | 0.98 (0.87, 1.11) | 0.760 |
| Respiratory disease | 2.19 (1.95, 2.47) | <0.001 | 1.01 (0.92, 1.12) | 0.790 | 0.96 (0.87, 1.06) | 0.460 |
| Chronic kidney disease | 2.09 (1.87, 2.34) | <0.001 | 1.38 (1.26, 1.52) | <0.001 | 1.10 (1.00, 1.21) | 0.049 |
| Hypertension | 1.44 (1.28, 1.62) | <0.001 | 1.07 (0.96, 1.19) | 0.210 | 0.96 (0.87, 1.07) | 0.470 |
| Type 2 diabetes mellitus | 1.91 (1.70, 2.15) | <0.001 | 1.18 (1.07, 1.30) | 0.001 | 0.92 (0.84, 1.02) | 0.100 |
| Osteoarthritis | 1.04 (0.98, 1.11) | 0.160 | 0.93 (0.88, 0.98) | 0.008 | 0.99 (0.94, 1.04) | 0.700 |
| Eye disease | 1.16 (1.09, 1.24) | <0.001 | 1.01 (0.96, 1.08) | 0.640 | 0.98 (0.93, 1.04) | 0.530 |
| Depression | 1.28 (1.14, 1.44) | <0.001 | 0.96 (0.86, 1.06) | 0.410 | 1.05 (0.95, 1.17) | 0.350 |
| Anxiety | 1.19 (1.07, 1.32) | 0.001 | 0.88 (0.80, 0.97) | 0.009 | 0.99 (0.90, 1.09) | 0.900 |

Notes: For all-cause mortality, Cox proportional hazard model adjusted for chronological age, sex, ethnicity, education level, Townsend deprivation index, alcohol intake frequency, smoking status, regular exercise, healthy diet, body mass index, medication use, and prevalent diseases at baseline was used. For cause-specific mortality and incident diseases, Fine and Gray’s competing risk models adjusted for chronological age, sex, ethnicity, education level, Townsend deprivation index, alcohol intake frequency, smoking status, regular exercise, healthy diet, and body mass index were used. The lowest quartiles of these scores were set as the reference, and the HRs (95% CI) of the highest quartile group were showed. CA, chronological age; AUC, area under the curve; HR, hazard ratio; CI, confidence interval.

Table S14. Associations of MetaboAgeMort and MetaboHealth score with all-cause mortality when mutually adjusted in the testing set.

|  | **AIC** | **MetaboAgeMort** | | **MetaboHealth** | |
| --- | --- | --- | --- | --- | --- |
|  |  | **HR (95% CI)** | **P value** | **HR (95% CI)** | **P value** |
| MetaboAgeMort + CA + sex | 113308.8 | 1.10 (1.10, 1.11) | --- | --- |  |
| MetaboHealth score + CA + sex | 113551.9 | --- | <2e-16 | 2.66 (2.52, 2.80) | <2e-16 |
| MetaboAgeMort + MetaboHealth score + CA + sex | 113238.2 | 1.07 (1.06, 1.08) | <2e-16 | 1.45 (1.33, 1.58) | <2e-16 |
| (Stepwise) MetaboAgeMort + MetaboHealth score + CA + sex | 113238.2 | 1.07 (1.06, 1.08) | <2e-16 | 1.45 (1.33, 1.58) | <2e-16 |
|  | | | | | |
| MetaboAgeMort + more covariates^a^ | 112262.6 | 1.07 (1.06,1.08) | --- | --- |  |
| MetaboHealth score + more covariates^a^ | 112268.8 | --- | <2e-16 | 2.01 (1.90, 2.13) | <2e-16 |
| MetaboAgeMort + MetaboHealth score + more covariates^a^ | 112191.1 | 1.04 (1.03, 1.05) | <2e-16 | 1.48 (1.35, 1.61) | <2e-16 |
| (Stepwise) MetaboAgeMort + MetaboHealth score + more covariates^a^ | 112184.9 | 1.04 (1.03, 1.05) | <2e-16 | 1.48 (1.35, 1.61) | <2e-16 |

Notes: AIC, Akaike Information Criterion; HR, hazard ratio; CI, confidence interval.

^a^ More covariates included ethnicity, education level, Townsend deprivation index, alcohol intake frequency, smoking status, regular exercise, healthy diet, body mass index, medication use, and prevalent diseases at baseline. AIC, Akaike Information Criterion; HR, hazard ratio; CI, confidence interval.

Table S15. Association of single modifiable factors with MetaboAgeMort.

| **Category** | **Field ID** | **Field** |  | **N** | | **Mean (SD)/N (%)** | | **Model 1** | | | | | | | | | | | | **Model 2** | |
| --- | --- | --- | --- | --- | --- | --- | --- | --- | --- | --- | --- | --- | --- | --- | --- | --- | --- | --- | --- | --- | --- |
|  |  |  |  |  |  |  |  | **All** | | | | **<60** | | **>=60** | | **Female** | | **Male** | |  |  |
|  |  |  |  |  |  |  |  | **β (95% CI)** | | **P value** | | **β (95% CI)** | | **β (95% CI)** | | **β (95% CI)** | | **β (95% CI)** | | **β (95% CI)** | |
| **Local environment** | Derived | Particulate matter air pollution (pm10) | 1-point | | 236,158 | | 19.10 (1.88) | | 0.17 (0.15, 0.19) | | 1.27E-65 | | -0.10 (-0.14, -0.06) | | 0.20 (0.16, 0.23) | | 0.15 (0.13, 0.18) | | 0.19 (0.17, 0.22) | | 0.14 (0.12, 0.16) |
|  |  |  | tertile 1 | |  | | 17.17 (0.93) | | ref. | |  | | ref. | | ref. | | ref. | | ref. | | ref. |
|  |  |  | tertile 2 | |  | | 19.00 (0.40) | | 0.43 (0.38, 0.48) | | 4.95E-73 | | 0.08 (-0.01, 0.17) | | 0.52 (0.43, 0.60) | | 0.42 (0.35, 0.48) | | 0.45 (0.38, 0.52) | | 0.34 (0.29, 0.38) |
|  |  |  | tertile 3 | |  | | 21.15 (1.28) | | 0.44 (0.39, 0.48) | | 2.75E-73 | | -0.17 (-0.26, -0.08) | | 0.49 (0.41, 0.58) | | 0.39 (0.33, 0.45) | | 0.50 (0.43, 0.57) | | 0.36 (0.31, 0.40) |
|  | Derived | Nitrogen dioxide air pollution | 1-point | | 236,162 | | 31.78 (9.40) | | 0.30 (0.28, 0.32) | | 4.57E-203 | | -0.00 (-0.04, 0.04) | | 0.36 (0.32, 0.39) | | 0.27 (0.25, 0.30) | | 0.34 (0.31, 0.37) | | 0.25 (0.23, 0.27) |
|  |  |  | tertile 1 | |  | | 22.48 (3.53) | | ref. | |  | | ref. | | ref. | | ref. | | ref. | | ref. |
|  |  |  | tertile 2 | |  | | 30.84 (1.98) | | 0.54 (0.49, 0.58) | | 8.35E-112 | | 0.25 (0.16, 0.34) | | 0.64 (0.56, 0.73) | | 0.48 (0.42, 0.54) | | 0.61 (0.54, 0.68) | | 0.43 (0.38, 0.47) |
|  |  |  | tertile 3 | |  | | 42.03 (7.50) | | 0.69 (0.64, 0.74) | | 1.90E-179 | | 0.04 (-0.05, 0.13) | | 0.83 (0.74, 0.91) | | 0.61 (0.55, 0.67) | | 0.79 (0.72, 0.86) | | 0.57 (0.53, 0.62) |
|  | 24006 | Particulate matter air pollution (pm2.5); 2010 | 1-point | | 222,977 | | 9.99 (1.06) | | 0.38 (0.36, 0.40) | | 3.96E-312 | | 0.08 (0.05, 0.12) | | 0.43 (0.40, 0.47) | | 0.35 (0.32, 0.37) | | 0.42 (0.39, 0.45) | | 0.31 (0.29, 0.33) |
|  |  |  | tertile 1 | |  | | 8.90 (0.43) | | ref. | |  | | ref. | | ref. | | ref. | | ref. | | ref. |
|  |  |  | tertile 2 | |  | | 9.93 (0.23) | | 0.43 (0.38, 0.48) | | 1.16E-68 | | 0.17 (0.08, 0.27) | | 0.51 (0.42, 0.59) | | 0.39 (0.33, 0.46) | | 0.48 (0.40, 0.55) | | 0.34 (0.30, 0.39) |
|  |  |  | tertile 3 | |  | | 11.14 (0.79 | | 0.80 (0.75, 0.85) | | 4.45E-229 | | 0.14 (0.05, 0.24) | | 0.93 (0.84, 1.02) | | 0.71 (0.65, 0.78) | | 0.90 (0.83, 0.97) | | 0.64 (0.60, 0.69) |
|  | 24008 | Particulate matter air pollution 2.5-10um; 2010 | 1-point | | 222,977 | | 6.41 (0.89) | | 0.09 (0.07, 0.11) | | 6.69E-19 | | 0.02 (-0.02, 0.06) | | 0.09 (0.06, 0.13) | | 0.09 (0.06, 0.11) | | 0.09 (0.06, 0.12) | | 0.06 (0.04, 0.08) |
|  |  |  | tertile 1 | |  | | 5.76 (0.10) | | ref. | |  | | ref. | | ref. | | ref. | | ref. | | ref. |
|  |  |  | tertile 2 | |  | | 6.11 (0.13) | | 0.25 (0.20, 0.30) | | 1.11E-24 | | 0.23 (0.14, 0.33) | | 0.23 (0.14, 0.32) | | 0.21 (0.14, 0.27) | | 0.31 (0.24, 0.38) | | 0.20 (0.16, 0.25) |
|  |  |  | tertile 3 | |  | | 7.36 (0.97) | | 0.28 (0.24, 0.33) | | 8.42E-31 | | 0.12 (0.03, 0.22) | | 0.32 (0.23, 0.40) | | 0.26 (0.20, 0.33) | | 0.31 (0.24, 0.38) | | 0.20 (0.16, 0.25) |
|  | 24020 | Average daytime sound level of noise pollution | 1-point | | 236,162 | | 55.43 (4.29) | | 0.06 (0.04, 0.08) | | 7.76E-10 | | -0.01 (-0.05, 0.03) | | 0.07 (0.04, 0.11) | | 0.05 (0.03, 0.08) | | 0.07 (0.04, 0.10) | | 0.03 (0.02, 0.05) |
|  |  |  | tertile 1 | |  | | 52.09 (0.80) | | ref. | |  | | ref. | | ref. | | ref. | | ref. | | ref. |
|  |  |  | tertile 2 | |  | | 54.33 (0.57) | | 0.12 (0.08, 0.17) | | 2.84E-07 | | -0.00 (-0.10, 0.09) | | 0.14 (0.06, 0.23) | | 0.10 (0.04, 0.16) | | 0.15 (0.08, 0.22) | | 0.11 (0.06, 0.15) |
|  |  |  | tertile 3 | |  | | 59.90 (4.68) | | 0.16 (0.11, 0.20) | | 6.65E-11 | | -0.03 (-0.12, 0.06) | | 0.14 (0.05, 0.22) | | 0.12 (0.06, 0.19) | | 0.19 (0.12, 0.27) | | 0.11 (0.06, 0.15) |
|  | 24021 | Average evening sound level of noise pollution | 1-point | | 236,162 | | 51.69 (4.29) | | 0.06 (0.04, 0.08) | | 7.32E-10 | | -0.01 (-0.05, 0.03) | | 0.07 (0.04, 0.11) | | 0.05 (0.03, 0.08) | | 0.07 (0.04, 0.10) | | 0.03 (0.02, 0.05) |
|  |  |  | tertile 1 | |  | | 48.34 (0.80) | | ref. | |  | | ref. | | ref. | | ref. | | ref. | | ref. |
|  |  |  | tertile 2 | |  | | 50.58 (0.57) | | 0.12 (0.07, 0.17) | | 6.38E-07 | | -0.01 (-0.10, 0.08) | | 0.14 (0.06, 0.23) | | 0.10 (0.04, 0.16) | | 0.15 (0.08, 0.22) | | 0.10 (0.06, 0.15) |
|  |  |  | tertile 3 | |  | | 56.15 (4.68) | | 0.15 (0.11, 0.20) | | 8.53E-11 | | -0.04 (-0.13, 0.05) | | 0.14 (0.05, 0.22) | | 0.12 (0.06, 0.19) | | 0.19 (0.12, 0.27) | | 0.11 (0.06, 0.15) |
|  | 24022 | Average night-time sound level of noise pollution | 1-point | | 236,162 | | 46.61 (4.29) | | 0.06 (0.04, 0.08) | | 6.78E-10 | | -0.01 (-0.05, 0.03) | | 0.07 (0.04, 0.11) | | 0.05 (0.03, 0.08) | | 0.07 (0.04, 0.10) | | 0.03 (0.02, 0.05) |
|  |  |  | tertile 1 | |  | | 43.27 (0.80) | | ref. | |  | | ref. | | ref. | | ref. | | ref. | | ref. |
|  |  |  | tertile 2 | |  | | 45.51 (0.57) | | 0.12 (0.08, 0.17) | | 2.97E-07 | | -0.00 (-0.09, 0.09) | | 0.14 (0.06, 0.23) | | 0.10 (0.04, 0.16) | | 0.15 (0.08, 0.22) | | 0.11 (0.06, 0.15) |
|  |  |  | tertile 3 | |  | | 51.08 (4.68) | | 0.16 (0.11, 0.20) | | 6.74E-11 | | -0.03 (-0.12, 0.06) | | 0.14 (0.05, 0.22) | | 0.12 (0.06, 0.19) | | 0.19 (0.12, 0.26) | | 0.11 (0.06, 0.15) |
|  | 24023 | Average 16-hour sound level of noise pollution | 1-point | | 236,162 | | 54.50 (4.29) | | 0.06 (0.04, 0.08) | | 7.57E-10 | | -0.01 (-0.05, 0.03) | | 0.07 (0.04, 0.11) | | 0.05 (0.03, 0.08) | | 0.07 (0.04, 0.10) | | 0.03 (0.02, 0.05) |
|  |  |  | tertile 1 | |  | | 51.15 (0.80) | | ref. | |  | | ref. | | ref. | | ref. | | ref. | | ref. |
|  |  |  | tertile 2 | |  | | 53.39 (0.57) | | 0.12 (0.07, 0.16) | | 1.07E-06 | | -0.01 (-0.10, 0.08) | | 0.14 (0.06, 0.23) | | 0.10 (0.04, 0.16) | | 0.15 (0.07, 0.22) | | 0.10 (0.06, 0.15) |
|  |  |  | tertile 3 | |  | | 58.96 (4.68) | | 0.15 (0.11, 0.20) | | 1.06E-10 | | -0.04 (-0.13, 0.05) | | 0.14 (0.05, 0.22) | | 0.12 (0.06, 0.18) | | 0.19 (0.12, 0.26) | | 0.11 (0.06, 0.15) |
|  | 24024 | Average 24-hour sound level of noise pollution | 1-point | | 236,162 | | 56.08 (4.29) | | 0.06 (0.04, 0.08) | | 7.73E-10 | | -0.01 (-0.05, 0.03) | | 0.07 (0.04, 0.11) | | 0.05 (0.03, 0.08) | | 0.07 (0.04, 0.10) | | 0.03 (0.02, 0.05) |
|  |  |  | tertile 1 | |  | | 52.73 (0.80) | | ref. | |  | | ref. | | ref. | | ref. | | ref. | | ref. |
|  |  |  | tertile 2 | |  | | 54.97 (0.57) | | 0.12 (0.07, 0.16) | | 9.92E-07 | | -0.01 (-0.10, 0.08) | | 0.14 (0.06, 0.23) | | 0.10 (0.04, 0.16) | | 0.15 (0.07, 0.22) | | 0.10 (0.06, 0.15) |
|  |  |  | tertile 3 | |  | | 60.54 (4.68) | | 0.15 (0.11, 0.20) | | 9.42E-11 | | -0.04 (-0.13, 0.05) | | 0.14 (0.05, 0.22) | | 0.12 (0.06, 0.19) | | 0.19 (0.12, 0.26) | | 0.11 (0.06, 0.15) |
|  | 24500 | Greenspace percentage, buffer 1000m | 1-point | | 215,353 | | 46.16 (21.31) | | -0.11 (-0.13, -0.09) | | 7.41E-28 | | 0.16 (0.12, 0.20) | | -0.14 (-0.17, -0.10) | | -0.10 (-0.12, -0.07) | | -0.14 (-0.17, -0.11) | | -0.11 (-0.13, -0.09) |
|  |  |  | tertile 1 | |  | | 23.65 (6.11) | | ref. | |  | | ref. | | ref. | | ref. | | ref. | | ref. |
|  |  |  | tertile 2 | |  | | 43.55 (6.15) | | 0.16 (0.11, 0.20) | | 6.00E-10 | | 0.53 (0.43, 0.63) | | 0.16 (0.07, 0.25) | | 0.15 (0.09, 0.22) | | 0.15 (0.07, 0.22) | | 0.11 (0.06, 0.16) |
|  |  |  | tertile 3 | |  | | 71.29 (11.95) | | -0.34 (-0.39, -0.29) | | 6.12E-42 | | 0.29 (0.19, 0.38) | | -0.42 (-0.50, -0.33) | | -0.28 (-0.35, -0.22) | | -0.41 (-0.49, -0.34) | | -0.31 (-0.36, -0.26) |
|  | 24501 | Domestic garden percentage, buffer 1000m | 1-point | | 215,353 | | 23.84 (11.04) | | 0.07 (0.05, 0.09) | | 3.61E-13 | | -0.01 (-0.05, 0.03) | | 0.12 (0.08, 0.15) | | 0.07 (0.05, 0.10) | | 0.07 (0.04, 0.10) | | 0.07 (0.05, 0.09) |
|  |  |  | tertile 1 | |  | | 11.71 (5.18) | | ref. | |  | | ref. | | ref. | | ref. | | ref. | | ref. |
|  |  |  | tertile 2 | |  | | 23.73 (2.77) | | 0.20 (0.15, 0.25) | | 6.05E-16 | | 0.06 (-0.03, 0.16) | | 0.28 (0.19, 0.37) | | 0.16 (0.10, 0.22) | | 0.24 (0.17, 0.32) | | 0.18 (0.13, 0.22) |
|  |  |  | tertile 3 | |  | | 36.07 (5.85) | | -0.16 (-0.21, -0.12) | | 3.75E-11 | | -0.32 (-0.42, -0.23) | | -0.07 (-0.16, 0.02) | | -0.15 (-0.22, -0.09) | | -0.18 (-0.25, -0.10) | | -0.10 (-0.14, -0.05) |
|  | 24502 | Water percentage, buffer 1000m | 1-point | | 215,353 | | 1.26 (2.40) | | -0.03 (-0.05, -0.01) | | 0.006 | | 0.00 (-0.04, 0.04) | | -0.05 (-0.08, -0.01) | | -0.03 (-0.06, -0.01) | | -0.02 (-0.05, 0.01) | | -0.01 (-0.03, 0.00) |
|  |  |  | tertile 1 | |  | | 0.14 (0.09) | | ref. | |  | | ref. | | ref. | | ref. | | ref. | | ref. |
|  |  |  | tertile 2 | |  | | 0.58 (0.18) | | -0.05 (-0.10, -0.01) | | 0.027 | | -0.05 (-0.15, 0.04) | | -0.04 (-0.13, 0.05) | | -0.06 (-0.13, -0.00) | | -0.03 (-0.11, 0.04) | | -0.03 (-0.08, 0.02) |
|  |  |  | tertile 3 | |  | | 3.06 (3.50) | | -0.07 (-0.12, -0.02) | | 0.007 | | 0.04 (-0.05, 0.14) | | -0.15 (-0.24, -0.07) | | -0.08 (-0.15, -0.02) | | -0.05 (-0.12, 0.03) | | -0.04 (-0.08, 0.01) |
|  | 24503 | Greenspace percentage, buffer 300m | 1-point | | 215,353 | | 36.23 (23.14) | | -0.06 (-0.08, -0.04) | | 1.56E-09 | | 0.15 (0.11, 0.19) | | -0.09 (-0.13, -0.06) | | -0.05 (-0.07, -0.02) | | -0.08 (-0.11, -0.05) | | -0.07 (-0.09, -0.05) |
|  |  |  | tertile 1 | |  | | 13.78 (5.18) | | ref. | |  | | ref. | | ref. | | ref. | | ref. | | ref. |
|  |  |  | tertile 2 | |  | | 31.26 (5.77) | | 0.30 (0.25, 0.35) | | 1.10E-32 | | 0.48 (0.39, 0.58) | | 0.28 (0.19, 0.37) | | 0.28 (0.22, 0.35) | | 0.31 (0.24, 0.39) | | 0.21 (0.17, 0.26) |
|  |  |  | tertile 3 | |  | | 63.66 (16.29) | | -0.22 (-0.27, -0.17) | | 2.30E-18 | | 0.28 (0.18, 0.37) | | -0.35 (-0.44, -0.26) | | -0.19 (-0.25, -0.13) | | -0.26 (-0.34, -0.19) | | -0.21 (-0.26, -0.17) |
|  | 24504 | Domestic garden percentage, buffer 300m | 1-point | | 215,353 | | 30.84 (14.59) | | 0.05 (0.03, 0.07) | | 8.82E-08 | | 0.03 (-0.01, 0.07) | | 0.09 (0.05, 0.13) | | 0.06 (0.03, 0.09) | | 0.05 (0.02, 0.08) | | 0.05 (0.03, 0.07) |
|  |  |  | tertile 1 | |  | | 14.31 (7.14) | | ref. | |  | | ref. | | ref. | | ref. | | ref. | | ref. |
|  |  |  | tertile 2 | |  | | 31.37 (3.89) | | 0.07 (0.02, 0.12) | | 0.005 | | -0.05 (-0.15, 0.04) | | 0.11 (0.02, 0.20) | | 0.04 (-0.03, 0.10) | | 0.11 (0.03, 0.18) | | 0.07 (0.02, 0.11) |
|  |  |  | tertile 3 | |  | | 46.82 (6.60) | | -0.22 (-0.27, -0.17) | | 8.89E-19 | | -0.15 (-0.25, -0.06) | | -0.15 (-0.24, -0.06) | | -0.22 (-0.28, -0.16) | | -0.22 (-0.30, -0.15) | | -0.15 (-0.19, -0.10) |
|  | 24505 | Water percentage, buffer 300m | 1-point | | 215,353 | | 0.89 (2.84) | | -0.07 (-0.09, -0.05) | | 1.95E-13 | | -0.02 (-0.06, 0.02) | | -0.10 (-0.14, -0.06) | | -0.07 (-0.10, -0.05) | | -0.08 (-0.11, -0.05) | | -0.06 (-0.08, -0.04) |
|  |  |  | tertile 1 | |  | | 0.01 (0.01) | | ref. | |  | | ref. | | ref. | | ref. | | ref. | | ref. |
|  |  |  | tertile 2 | |  | | 0.17 (0.11) | | -0.10 (-0.15, -0.05) | | 4.05E-05 | | 0.05 (-0.04, 0.15) | | -0.17 (-0.25, -0.08) | | -0.09 (-0.16, -0.03) | | -0.12 (-0.19, -0.04) | | -0.08 (-0.13, -0.03) |
|  |  |  | tertile 3 | |  | | 2.49 (4.50) | | -0.19 (-0.24, -0.14) | | 5.93E-14 | | 0.04 (-0.06, 0.14) | | -0.31 (-0.40, -0.22) | | -0.18 (-0.25, -0.12) | | -0.19 (-0.27, -0.12) | | -0.15 (-0.20, -0.11) |
|  | 24506 | Natural environment percentage, buffer 1000m | 1-point | | 237,173 | | 41.92 (25.30) | | -0.15 (-0.17, -0.13) | | 5.08E-52 | | 0.14 (0.10, 0.17) | | -0.17 (-0.21, -0.14) | | -0.13 (-0.16, -0.11) | | -0.17 (-0.20, -0.14) | | -0.13 (-0.15,- 0.12) |
|  |  |  | tertile 1 | |  | | 15.03 (6.74) | | ref. | |  | | ref. | | ref. | | ref. | | ref. | | ref. |
|  |  |  | tertile 2 | |  | | 38.90 (7.54) | | 0.02 (-0.03, 0.06) | | 0.527 | | 0.37 (0.28, 0.46) | | 0.03 (-0.06, 0.11) | | 0.01 (-0.05, 0.07) | | 0.01 (-0.06, 0.08) | | 0.00 (-0.05, 0.04) |
|  |  |  | tertile 3 | |  | | 71.84 (13.84) | | -0.46 (-0.51, -0.41) | | 7.73E-82 | | 0.14 (0.05, 0.24) | | -0.53 (-0.62, -0.45) | | -0.40 (-0.46, -0.34) | | -0.54 (-0.61, -0.46) | | -0.40 (-0.44, -0.35) |
|  | 24507 | Natural environment percentage, buffer 300m | 1-point | | 237,173 | | 27.15 (25.19) | | -0.12 (-0.14, -0.10) | | 6.21E-33 | | 0.11 (0.07, 0.14) | | -0.14 (-0.18, -0.11) | | -0.09 (-0.12, -0.07) | | -0.15 (-0.18, -0.12) | | -0.11 (-0.13, -0.09) |
|  |  |  | tertile 1 | |  | | 3.83 (3.62) | | ref. | |  | | ref. | | ref. | | ref. | | ref. | | ref. |
|  |  |  | tertile 2 | |  | | 20.94 (6.26) | | 0.02 (-0.03, 0.07) | | 0.371 | | 0.27 (0.18, 0.36) | | 0.01 (-0.08, 0.09) | | 0.04 (-0.02, 0.10) | | 0.00 (-0.07, 0.07) | | -0.01 (-0.05, 0.04) |
|  |  |  | tertile 3 | |  | | 57.02 (19.47) | | -0.36 (-0.40, -0.31) | | 1.19E-49 | | 0.16 (0.07, 0.25) | | -0.43 (-0.51, -0.34) | | -0.29 (-0.35, -0.23) | | -0.44 (-0.51, -0.37) | | -0.31 (-0.36, -0.27) |
| **Psychosocial** | 1920 | Mood swings | yes vs. no (ref.) | | 233,589 | | 105,705 (45.25%) | | 0.71 (0.67, 0.75) | | 1.54E-280 | | -0.12 (-0.19, -0.04) | | 0.47 (0.40, 0.54) | | 0.63 (0.58, 0.68) | | 0.77 (0.71, 0.83) | | 0.49 (0.45, 0.53) |
|  | 1930 | Miserableness | yes vs. no (ref.) | | 235,352 | | 100,446 (42.68%) | | 0.62 (0.58, 0.66) | | 7.27E-210 | | 0.06 (-0.02, 0.13) | | 0.31 (0.23, 0.38) | | 0.53 (0.48, 0.58) | | 0.70 (0.64, 0.76) | | 0.44 (0.40, 0.47) |
|  | 1940 | Irritability | yes vs. no (ref.) | | 228,777 | | 64,303 (28.11%) | | 0.33 (0.29, 0.38) | | 3.54E-51 | | -0.54 (-0.62, -0.46) | | 0.04 (-0.05, 0.12) | | 0.28 (0.22, 0.34) | | 0.34 (0.27, 0.40) | | 0.21 (0.17, 0.26) |
|  | 1950 | Sensitivity / hurt feelings | yes vs. no (ref.) | | 232,538 | | 128,439 (55.23%) | | 0.19 (0.15, 0.23) | | 8.59E-21 | | 0.22 (0.15, 0.30) | | 0.02 (-0.05, 0.09) | | 0.23 (0.18, 0.29) | | 0.14 (0.08, 0.20) | | 0.16 (0.12, 0.19) |
|  | 1960 | Fed-up feelings | yes vs. no (ref.) | | 234,335 | | 94,679 (40.40%) | | 1.04 (1.00, 1.08) | | 0.00E+00 | | 0.39 (0.31, 0.46) | | 0.85 (0.78, 0.93) | | 0.98 (0.93, 1.03) | | 1.10 (1.03, 1.16) | | 0.74 (0.70, 0.78) |
|  | 1970 | Nervous feelings | yes vs. no (ref.) | | 233,204 | | 54,313 (23.29%) | | 0.07 (0.02, 0.11) | | 0.003 | | 0.10 (0.01, 0.18) | | -0.12 (-0.20, -0.03) | | 0.10 (0.05, 0.16) | | 0.08 (0.00, 0.15) | | 0.32 (0.28, 0.36) |
|  | 1980 | Worrier / anxious feelings | yes vs. no (ref.) | | 233,032 | | 130,727 (56.10%) | | -0.02 (-0.05, 0.02) | | 0.450 | | 0.13 (0.06, 0.21) | | -0.18 (-0.25, -0.11) | | -0.02 (-0.07, 0.03) | | 0.02 (-0.04, 0.08) | | 0.11 (0.08, 0.15) |
|  | 1990 | Tense / 'highly strung' | yes vs. no (ref.) | | 231,221 | | 40,163 (17.37%) | | 0.22 (0.17, 0.27) | | 3.17E-17 | | -0.09 (-0.19, 0.00) | | 0.05 (-0.05, 0.15) | | 0.11 (0.05, 0.17) | | 0.40 (0.32, 0.48) | | 0.33 (0.28, 0.38) |
|  | 2000 | Worry too long after embarrassment | yes vs. no (ref.) | | 229,749 | | 109,330 (47.59%) | | -0.18 (-0.22, -0.14) | | 7.28E-20 | | -0.14 (-0.21, -0.06) | | -0.51 (-0.59, -0.44) | | -0.12 (-0.17, -0.07) | | -0.24 (-0.30, -0.18) | | -0.08 (-0.12, -0.05) |
|  | 2010 | Suffer from 'nerves' | yes vs. no (ref.) | | 230,391 | | 48,618 (21.10%) | | 0.31 (0.26, 0.36) | | 1.24E-37 | | 0.09 (-0.00, 0.18) | | 0.06 (-0.03, 0.15) | | 0.40 (0.34, 0.46) | | 0.26 (0.19, 0.33) | | 0.36 (0.32, 0.41) |
|  | 2020 | Loneliness, isolation | yes vs. no (ref.) | | 235,535 | | 42,654 (18.11%) | | 1.01 (0.96, 1.06) | | 0.00E+00 | | 0.82 (0.72, 0.91) | | 0.89 (0.80, 0.99) | | 0.85 (0.79, 0.91) | | 1.29 (1.20, 1.37) | | 0.75 (0.70, 0.79) |
|  | 2030 | Guilty feelings | yes vs. no (ref.) | | 232,936 | | 66,608 (28.59%) | | 0.06 (0.02, 0.10) | | 0.006 | | 0.04 (-0.04, 0.13) | | -0.26 (-0.34, -0.18) | | -0.02 (-0.07, 0.03) | | 0.19 (0.12, 0.26) | | 0.04 (0.00, 0.08) |
|  | 2110 | Able to confide | yes vs. no (ref.) | | 231,722 | | 59,410 (25.64%) | | 0.49 (0.44, 0.53) | | 5.29E-103 | | 0.94 (0.85, 1.03) | | 0.51 (0.44, 0.59) | | 0.46 (0.40, 0.52) | | 0.57 (0.50, 0.63) | | 0.41 (0.36, 0.45) |
| **Socioeconomic** | 738 | Average total household income before tax |  | | 206,846 | |  | | *Global P* | | 0.00E+00 | |  | |  | |  | |  | |  |
|  |  |  | Less than 18,000 | |  | | 47,323 (22.88%) | | ref. | |  | | ref. | | ref. | | ref. | | ref. | | ref. |
|  |  |  | 18,000 to 30,999 | |  | | 53,735 (25.98%) | | -1.18 (-1.24, -1.12) | | 0.00E+00 | | -1.33 (-1.46, -1.20) | | -1.71 (-1.80, -1.62) | | -1.04 (-1.11, -0.97) | | -1.42 (-1.50, -1.33) | | -1.00 (-1.06, -0.95) |
|  |  |  | 31,000 to 51,999 | |  | | 54,110 (26.16%) | | -1.83 (-1.89, -1.77) | | 0.00E+00 | | -2.72 (-2.84, -2.60) | | -3.12 (-3.22, -3.01) | | -1.67 (-1.75, -1.59) | | -2.11 (-2.20, -2.02) | | -1.58 (-1.64, -1.53) |
|  |  |  | 52,000 to 100,000 | |  | | 41,344 (19.99%) | | -2.43 (-2.49, -2.36) | | 0.00E+00 | | -3.71 (-3.83, -3.58) | | -4.11 (-4.25, -3.98) | | -2.25 (-2.34, -2.16) | | -2.69 (-2.78, -2.59) | | -2.07 (-2.13, -2.01) |
|  |  |  | Greater than 100,000 | |  | | 10,334 (5.00%) | | -3.34 (-3.44, -3.24) | | 0.00E+00 | | -4.84 (-5.01, -4.66) | | -4.91 (-5.17, -4.65) | | -3.09 (-3.23, -2.94) | | -3.64 (-3.79, -3.50) | | -2.81 (-2.91, -2.71) |
|  | Derived | Qualifications | Higher education | | 239,291 | | 77,389 (32.34%) | | ref. | |  | | ref. | | ref. | | ref. | | ref. | | ref. |
|  |  |  | Lower education | |  | | 161,902 (67.66%) | | 1.36 (1.32, 1.41) | | 0.00E+00 | | 1.79 (1.71, 1.86) | | 1.92 (1.85, 2.00) | | 1.22 (1.17, 1.28) | | 1.56 (1.50, 1.62) | | 1.00 (0.96, 1.04) |
|  | Derived | Current employment status | Unemployed | | 238,714 | | 17,447 (7.31%) | | ref. | |  | | ref. | | ref. | | ref. | | ref. | | ref. |
|  |  |  | Employed | |  | | 221,267 (92.69%) | | -1.94 (-2.01, -1.87) | | 0.00E+00 | | -2.53 (-2.65, -2.41) | | 0.05 (-0.14, 0.25) | | -1.11 (-1.20, -1.01) | | -2.87 (-2.98, -2.75) | | -1.60 (-1.67, -1.53) |
|  | 189 | Townsend deprivation index at recruitment | 1-point | | 239,291 | | -1.41 (3.04) | | 0.69 (0.67, 0.71) | | 0.00E+00 | | 0.34 (0.30, 0.37) | | 0.72 (0.68, 0.75) | | 0.63 (0.61, 0.66) | | 0.76 (0.73, 0.79) | | 0.54 (0.53, 0.56) |
|  |  |  | tertile 1 | |  | | -4.27 (0.72) | | ref. | |  | | ref. | | ref. | | ref. | | ref. | | ref. |
|  |  |  | tertile 2 | |  | | -2.16 (0.69) | | 0.44 (0.40, 0.49) | | 2.72E-78 | | 0.17 (0.08, 0.26) | | 0.53 (0.45, 0.62) | | 0.36 (0.30, 0.42) | | 0.53 (0.46, 0.60) | | 0.34 (0.29, 0.38) |
|  |  |  | tertile 3 | |  | | 2.19 (2.22) | | 1.41 (1.37, 1.46) | | 0.00E+00 | | 0.63 (0.54, 0.72) | | 1.46 (1.37, 1.54) | | 1.28 (1.21, 1.34) | | 1.59 (1.52, 1.66) | | 1.11 (1.06, 1.15) |
| **Medical history** | Derived | Cardiovascular disease at baseline | yes vs. no (ref.) | | 102,933 | | 16,014 (6.69%) | | 3.30 (3.23, 3.38) | | 0.00E+00 | | 6.09 (5.89, 6.29) | | 3.93 (3.82, 4.04) | | 3.16 (3.04, 3.28) | | 3.27 (3.17, 3.37) | | 2.79 (2.72, 2.86) |
|  | Derived | Hypertension at baseline | yes vs. no (ref.) | | 102,933 | | 133,563 (55.82%) | | 1.43 (1.39, 1.47) | | 0.00E+00 | | 3.59 (3.52, 3.66) | | 2.19 (2.12, 2.27) | | 1.39 (1.34, 1.44) | | 1.54 (1.48, 1.60) | | 0.69 (0.65, 0.72) |
|  | Derived | Cancer at baseline | yes vs. no (ref.) | | 102,933 | | 19,949 (8.34%) | | 0.48 (0.41, 0.54) | | 3.38E-41 | | 2.10 (1.94, 2.26) | | 0.92 (0.81, 1.03) | | 0.40 (0.32, 0.48) | | 0.61 (0.49, 0.73) | | 0.51 (0.44, 0.57) |
|  | Derived | Diabetes mellitus at baseline | yes vs. no (ref.) | | 102,933 | | 13,980 (5.84%) | | 5.09 (5.02, 5.17) | | 0.00E+00 | | 7.22 (7.04, 7.40) | | 5.26 (5.14, 5.39) | | 5.52 (5.39, 5.64) | | 4.77 (4.67, 4.88) | | 3.94 (3.86, 4.02) |
|  | Derived | Depression at baseline | yes vs. no (ref.) | | 102,933 | | 14,985 (6.26%) | | 1.30 (1.22, 1.38) | | 2.93E-231 | | 1.27 (1.13, 1.41) | | 0.84 (0.69, 1.00) | | 1.16 (1.07, 1.25) | | 1.50 (1.36, 1.63) | | 0.92 (0.84, 0.99) |
|  | Derived | Chronic obstructive pulmonary disease at baseline | yes vs. no (ref.) | | 102,933 | | 2,225 (0.93%) | | 4.12 (3.92, 4.32) | | 0.00E+00 | | 7.40 (6.86, 7.94) | | 4.57 (4.29, 4.85) | | 3.76 (3.49, 4.03) | | 4.44 (4.15, 4.73) | | 3.75 (3.56, 3.94) |
|  | 6146 | Attendance/disability/mobility allowance | yes vs. no (ref.) | | 102,239 | | 13,190 (5.55%) | | 3.77 (3.69, 3.86) | | 0.00E+00 | | 4.92 (4.74, 5.10) | | 4.33 (4.19, 4.46) | | 3.48 (3.37, 3.59) | | 4.05 (3.93, 4.17) | | 2.96 (2.88, 3.04) |
|  | 6149 | Mouth/teeth dental problems | yes vs. no (ref.) | | 102,695 | | 95,021 (39.80%) | | 1.01 (0.97, 1.05) | | 0.00E+00 | | 1.30 (1.23, 1.38) | | 1.58 (1.51, 1.65) | | 0.97 (0.92, 1.02) | | 1.09 (1.03, 1.15) | | 0.82 (0.78, 0.86) |
| **Early life and sexual health** | 1677 | Breastfed as a baby | yes vs. no (ref.) | | 182,575 | | 132,014 (72.31%) | | -0.48 (-0.53, -0.43) | | 4.36E-80 | | 1.50 (1.42, 1.59) | | -0.01 (-0.11, 0.09) | | -0.49 (-0.55, -0.43) | | -0.47 (-0.55, -0.39) | | -0.41 (-0.46, -0.37) |
|  | 1787 | Maternal smoking around birth | yes vs. no (ref.) | | 206,576 | | 61,246 (29.65%) | | 0.58 (0.54, 0.63) | | 3.62E-142 | | 1.10 (1.01, 1.18) | | -0.17 (-0.25, -0.08) | | 0.50 (0.44, 0.56) | | 0.67 (0.60, 0.74) | | 0.32 (0.28, 0.36) |
|  | 1687 | Comparative body size at age 10 |  | | 235,237 | |  | | *Global P* | | 0.00E+00 | |  | |  | |  | |  | |  |
|  |  |  | About average | |  | | 119,613 (50.85%) | | ref. | |  | | ref. | | ref. | | ref. | | ref. | | ref. |
|  |  |  | Thinner | |  | | 78,524 (33.38%) | | 0.18 (0.14, 0.22) | | 6.07E-17 | | 0.02 (-0.07, 0.10) | | 0.30 (0.22, 0.38) | | 0.21 (0.16, 0.27) | | 0.15 (0.08, 0.21) | | 0.42 (0.38, 0.46) |
|  |  |  | Plumper | |  | | 37,100 (15.77%) | | 0.92 (0.87, 0.98) | | 1.54E-236 | | 0.85 (0.74, 0.95) | | 0.56 (0.46, 0.67) | | 0.97 (0.90, 1.03) | | 0.86 (0.77, 0.95) | | 0.09 (0.04, 0.14) |
|  | 1697 | Comparative height size at age 10 |  | | 235,456 | |  | | *Global P* | | 1.10E-16 | |  | |  | |  | |  | |  |
|  |  |  | About average | |  | | 128,013 (54.37%) | | ref. | |  | | ref. | | ref. | | ref. | | ref. | | ref. |
|  |  |  | Shorter | |  | | 47,663 (20.24%) | | -0.01 (-0.06, 0.04) | | 0.746 | | -0.15 (-0.24, -0.05) | | -0.03 (-0.12, 0.06) | | 0.00 (-0.06, 0.07) | | -0.02 (-0.09, 0.06) | | 0.05 (0.00, 0.10) |
|  |  |  | Taller | |  | | 59,780 (25.39%) | | 0.04 (-0.00, 0.09) | | 0.071 | | -0.16 (-0.25, -0.07) | | -0.07 (-0.16, 0.01) | | 0.02 (-0.04, 0.08) | | 0.07 (-0.00, 0.14) | | 0.05 (0.01, 0.09) |
|  | 2139 | Age first sexual intercourse | 1-point | | 210,104 | | 19.10 (3.88) | | -0.35 (-0.37, -0.33) | | 4.88E-250 | | 0.07 (0.03, 0.11) | | -0.09 (-0.12, -0.05) | | -0.23 (-0.25, -0.20) | | -0.45 (-0.48, -0.42) | | -0.19 (-0.21, -0.17) |
|  |  |  | tertile 1 | |  | | 15.85 (1.31) | | ref. | |  | | ref. | | ref. | | ref. | | ref. | | ref. |
|  |  |  | tertile 2 | |  | | 18.81 (0.82) | | -0.72 (-0.77, -0.68) | | 2.45E-195 | | 0.01 (-0.08, 0.11) | | -0.44 (-0.53, -0.36) | | -0.46 (-0.52, -0.39) | | -0.95 (-1.02, -0.88) | | -0.49 (-0.54, -0.44) |
|  |  |  | tertile 3 | |  | | 23.76 (3.89) | | -1.02 (-1.07, -0.97) | | 9.88E-324 | | 0.47 (0.38, 0.56) | | -0.23 (-0.33, -0.14) | | -0.68 (-0.75, -0.61) | | -1.27 (-1.34, -1.19) | | -0.61 (-0.66, -0.56) |
|  | 2149 | Lifetime number of sexual partners | 1-point | | 195,903 | | 7.58 (66.50) | | 0.02 (0.00, 0.04) | | 0.033 | | -0.06 (-0.10, -0.02) | | -0.05 (-0.08, -0.01) | | -0.02 (-0.04, 0.01) | | 0.04 (0.01, 0.07) | | 0.02 (-0.00, 0.04) |
|  |  |  | tertile 1 | |  | | 1.31 (0.46) | | ref. | |  | | ref. | | ref. | | ref. | | ref. | | ref. |
|  |  |  | tertile 2 | |  | | 3.88 (0.83) | | 0.21 (0.16, 0.26) | | 9.61E-16 | | -0.85 (-0.95, -0.75) | | -0.15 (-0.24, -0.06) | | 0.09 (0.03, 0.16) | | 0.40 (0.32, 0.48) | | 0.13 (0.08, 0.18) |
|  |  |  | tertile 3 | |  | | 19.46 (119.16) | | 0.17 (0.11, 0.22) | | 4.33E-10 | | -1.96 (-2.05, -1.87) | | -0.62 (-0.71, -0.52) | | -0.09 (-0.16, -0.02) | | 0.43 (0.35, 0.51) | | 0.08 (0.03, 0.13) |
|  | 2159 | Ever had same-sex intercourse | yes vs. no (ref.) | | 216,172 | | 7334 (3.39%) | | 0.40 (0.29, 0.51) | | 5.88E-13 | | -1.00 (-1.19, -0.82) | | -0.18 (-0.44, 0.08) | | 0.06 (-0.10, 0.22) | | 0.64 (0.48, 0.79) | | 0.39 (0.29, 0.50) |
| **Physical measures** | 23099 | Body fat percentage | 1-point | | 235,635 | | 31.44 (8.54) | | 1.80 (1.78, 1.83) | | 0.00E+00 | | 2.68 (2.64, 2.73) | | 2.01 (1.96, 2.05) | | 1.30 (1.28, 1.33) | | 1.39 (1.36, 1.42) | | 0.31 (0.27, 0.36) |
|  |  |  | tertile 1 | |  | | 22.14 (3.88) | | ref. | |  | | ref. | | ref. | | ref. | | ref. | | ref. |
|  |  |  | tertile 2 | |  | | 31.16 (2.39) | | 1.79 (1.74, 1.84) | | 0.00E+00 | | 2.82 (2.72, 2.91) | | 2.16 (2.07, 2.25) | | 0.75 (0.69, 0.81) | | 1.09 (1.02, 1.16) | | 0.44 (0.39, 0.50) |
|  |  |  | tertile 3 | |  | | 41.14 (4.12) | | 3.55 (3.49, 3.61) | | 0.00E+00 | | 5.51 (5.39, 5.62) | | 4.13 (4.02, 4.25) | | 2.94 (2.89, 3.00) | | 3.11 (3.04, 3.18) | | 0.35 (0.26, 0.43) |
|  | 23105 | Basal metabolic rate | 1-point | | 235,757 | | 6654.18 (1366.48) | | 1.51 (1.48, 1.54) | | 0.00E+00 | | 0.71 (0.65, 0.77) | | 1.00 (0.94, 1.05) | | 1.19 (1.17, 1.22) | | 0.85 (0.82, 0.88) | | -0.51 (-0.55, -0.47) |
|  |  |  | tertile 1 | |  | | 5250.19 (326.78) | | ref. | |  | | ref. | | ref. | | ref. | | ref. | | ref. |
|  |  |  | tertile 2 | |  | | 6450.23 (451.48) | | 1.79 (1.74, 1.84) | | 0.00E+00 | | 1.00 (0.90, 1.11) | | 1.49 (1.39, 1.59) | | 0.58 (0.52, 0.64) | | 0.31 (0.24, 0.38) | | -0.30 (-0.36, -0.25) |
|  |  |  | tertile 3 | |  | | 8265.93 (826.61) | | 2.89 (2.82, 2.97) | | 0.00E+00 | | 1.26 (1.12, 1.41) | | 2.07 (1.94, 2.21) | | 2.35 (2.28, 2.41) | | 1.73 (1.66, 1.80) | | -0.92 (-1.01, -0.83) |
|  | Derived | Leg fat percentage | 1-point | | 235,747 | | 31.85 (10.66) | | 2.63 (2.59, 2.66) | | 0.00E+00 | | 4.10 (4.03, 4.16) | | 2.97 (2.90, 3.04) | | 1.35 (1.33, 1.38) | | 1.30 (1.28, 1.33) | | 0.31 (0.24, 0.38) |
|  |  |  | tertile 1 | |  | | 19.51 (3.33) | | ref. | |  | | ref. | | ref. | | ref. | | ref. | | ref. |
|  |  |  | tertile 2 | |  | | 32.22 (4.53) | | 2.46 (2.40, 2.52) | | 0.00E+00 | | 3.57 (3.45, 3.69) | | 2.49 (2.38, 2.60) | | 0.75 (0.69, 0.81) | | 1.01 (0.94, 1.08) | | 0.78 (0.72, 0.84) |
|  |  |  | tertile 3 | |  | | 43.86 (3.52) | | 4.26 (4.18, 4.34) | | 0.00E+00 | | 6.96 (6.81, 7.11) | | 4.81 (4.66, 4.95) | | 3.01 (2.95, 3.07) | | 2.87 (2.80, 2.94) | | 0.46 (0.37, 0.56) |
|  | Derived | Arm fat percentage | 1-point | | 235,702 | | 29.91 (10.18) | | 1.91 (1.89, 1.94) | | 0.00E+00 | | 2.40 (2.35, 2.45) | | 2.07 (2.02, 2.11) | | 1.44 (1.42, 1.46) | | 1.41 (1.38, 1.44) | | 0.35 (0.29, 0.40) |
|  |  |  | tertile 1 | |  | | 19.39 (2.93) | | ref. | |  | | ref. | | ref. | | ref. | | ref. | | ref. |
|  |  |  | tertile 2 | |  | | 28.46 (3.06) | | 1.83 (1.78, 1.88) | | 0.00E+00 | | 2.48 (2.38, 2.58) | | 2.06 (1.96, 2.15) | | 0.81 (0.75, 0.86) | | 1.08 (1.01, 1.15) | | 0.43 (0.38, 0.49) |
|  |  |  | tertile 3 | |  | | 41.93 (5.93) | | 3.71 (3.65, 3.78) | | 0.00E+00 | | 4.90 (4.78, 5.03) | | 4.12 (4.01, 4.24) | | 3.13 (3.07, 3.19) | | 3.03 (2.96, 3.10) | | 0.36 (0.28, 0.45) |
|  | 23127 | Trunk fat percentage | 1-point | | 235,599 | | 31.25 (7.96) | | 1.34 (1.32, 1.36) | | 0.00E+00 | | 2.05 (2.01, 2.09) | | 1.48 (1.44, 1.52) | | 1.15 (1.13, 1.18) | | 1.31 (1.28, 1.34) | | 0.19 (0.16, 0.22) |
|  |  |  | tertile 1 | |  | | 22.64 (4.49) | | ref. | |  | | ref. | | ref. | | ref. | | ref. | | ref. |
|  |  |  | tertile 2 | |  | | 31.28 (1.91) | | 1.20 (1.15, 1.24) | | 0.00E+00 | | 2.04 (1.95, 2.12) | | 1.54 (1.45, 1.62) | | 0.78 (0.73, 0.84) | | 1.04 (0.97, 1.11) | | 0.21 (0.16, 0.25) |
|  |  |  | tertile 3 | |  | | 39.91 (4.15) | | 2.96 (2.91, 3.01) | | 0.00E+00 | | 4.59 (4.49, 4.68) | | 3.35 (3.26, 3.44) | | 2.67 (2.61, 2.73) | | 3.02 (2.95, 3.09) | | 0.47 (0.40, 0.53) |
|  | Derived | Forced vital capacity (FVC) | 1-point | | 218,594 | | 3.62 (1.03) | | -1.05 (-1.08, -1.02) | | 0.00E+00 | | -2.44 (-2.48, -2.39) | | -1.77 (-1.82, -1.73) | | -0.72 (-0.74, -0.69) | | -0.96 (-0.99, -0.93) | | -0.69 (-0.72, -0.67) |
|  |  |  | tertile 1 | |  | | 2.59 (0.41) | | ref. | |  | | ref. | | ref. | | ref. | | ref. | | ref. |
|  |  |  | tertile 2 | |  | | 3.52 (0.25) | | -0.95 (-1.00, -0.90) | | 3.86E-296 | | -2.79 (-2.89, -2.70) | | -1.95 (-2.04, -1.86) | | -1.18 (-1.25, -1.12) | | -1.38 (-1.45, -1.31) | | -0.48 (-0.53, -0.43) |
|  |  |  | tertile 3 | |  | | 4.76 (0.76) | | -2.22 (-2.28, -2.15) | | 0.00E+00 | | -5.58 (-5.70, -5.46) | | -4.07 (-4.18, -3.95) | | -1.68 (-1.75, -1.61) | | -2.27 (-2.35, -2.19) | | -1.39 (-1.45, -1.32) |
|  | Derived | Forced expiratory volume in 1-second (FEV1) | 1-point | | 218,594 | | 2.74 (0.79) | | -1.15 (-1.17, -1.12) | | 0.00E+00 | | -2.77 (-2.81, -2.72) | | -1.93 (-1.97, -1.88) | | -0.80 (-0.83, -0.77) | | -1.07 (-1.10, -1.04) | | -0.89 (-0.91, -0.86) |
|  |  |  | tertile 1 | |  | | 1.93 (0.34) | | ref. | |  | | ref. | | ref. | | ref. | | ref. | | ref. |
|  |  |  | tertile 2 | |  | | 2.67 (0.19) | | -1.01 (-1.06, -0.96) | | 0.00E+00 | | -3.02 (-3.12, -2.93) | | -2.03 (-2.11, -1.94) | | -1.19 (-1.26, -1.13) | | -1.48 (-1.55, -1.41) | | -0.68 (-0.73, -0.64) |
|  |  |  | tertile 3 | |  | | 3.63 (0.51) | | -2.26 (-2.33, -2.20) | | 0.00E+00 | | -5.96 (-6.08, -5.85) | | -4.13 (-4.24, -4.02) | | -1.73 (-1.80, -1.66) | | -2.36 (-2.43, -2.28) | | -1.68 (-1.74, -1.62) |
|  | Derived | Peak expiratory flow (PEF) | 1-point | | 218,594 | | 372.45 (129.25) | | -0.76 (-0.78, -0.73) | | 0.00E+00 | | -1.80 (-1.84, -1.75) | | -1.35 (-1.39, -1.31) | | -0.48 (-0.51, -0.45) | | -0.78 (-0.81, -0.74) | | -0.72 (-0.74, -0.69) |
|  |  |  | tertile 1 | |  | | 237.96 (57.76) | | ref. | |  | | ref. | | ref. | | ref. | | ref. | | ref. |
|  |  |  | tertile 2 | |  | | 361.52 (30.24) | | -0.68 (-0.73, -0.63) | | 6.10E-165 | | -1.97 (-2.06, -1.88) | | -1.41 (-1.50, -1.32) | | -0.77 (-0.83, -0.71) | | -1.06 (-1.13, -0.99) | | -0.63 (-0.67, -0.58) |
|  |  |  | tertile 3 | |  | | 518.23 (80.12) | | -1.57 (-1.63, -1.51) | | 0.00E+00 | | -3.91 (-4.02, -3.80) | | -2.91 (-3.02, -2.81) | | -1.10 (-1.16, -1.03) | | -1.76 (-1.84, -1.69) | | -1.47 (-1.53, -1.42) |
|  | Derived | Lung function | 1-point | | 218,594 | | 0.03 (0.94) | | -1.16 (-1.19, -1.14) | | 0.00E+00 | | -2.72 (-2.77, -2.68) | | -1.97 (-2.01, -1.92) | | -0.77 (-0.80, -0.74) | | -1.07 (-1.10, -1.03) | | -0.91 (-0.94, -0.89) |
|  |  |  | tertile 1 | |  | | -0.93 (0.39) | | ref. | |  | | ref. | | ref. | | ref. | | ref. | | ref. |
|  |  |  | tertile 2 | |  | | -0.06 (0.23) | | -0.96 (-1.01, -0.91) | | 2.51E-308 | | -2.97 (-3.06, -2.87) | | -2.05 (-2.13, -1.96) | | -1.16 (-1.22, -1.10) | | -1.48 (-1.56, -1.41) | | -0.65 (-0.70, -0.60) |
|  |  |  | tertile 3 | |  | | 1.09 (0.60) | | -2.31 (-2.37, -2.24) | | 0.00E+00 | | -5.88 (-6.00, -5.76) | | -4.24 (-4.35, -4.13) | | -1.69 (-1.75, -1.62) | | -2.39 (-2.46, -2.31) | | -1.75 (-1.81, -1.69) |
|  | Derived | Diastolic blood pressure, automated reading | 1-point | | 227,513 | | 82.29 (10.11) | | 0.23 (0.21, 0.24) | | 4.32E-111 | | 1.03 (0.99, 1.07) | | -0.21 (-0.25, -0.17) | | 0.33 (0.31, 0.36) | | 0.12 (0.09, 0.15) | | -0.19 (-0.21, -0.17) |
|  |  |  | tertile 1 | |  | | 71.54 (4.81) | | ref. | |  | | ref. | | ref. | | ref. | | ref. | | ref. |
|  |  |  | tertile 2 | |  | | 82.17 (2.56) | | 0.18 (0.14, 0.23) | | 2.01E-14 | | 1.34 (1.24, 1.43) | | -0.37 (-0.45, -0.28) | | 0.36 (0.30, 0.42) | | -0.01 (-0.08, 0.06) | | -0.33 (-0.37, -0.28) |
|  |  |  | tertile 3 | |  | | 93.77 (5.98) | | 0.52 (0.47, 0.57) | | 2.87E-97 | | 2.41 (2.32, 2.50) | | -0.42 (-0.51, -0.33) | | 0.77 (0.70, 0.83) | | 0.30 (0.23, 0.38) | | -0.41 (-0.46, -0.36) |
|  | Derived | Systolic blood pressure, automated reading | 1-point | | 227,510 | | 138.03 (18.58) | | 0.20 (0.18, 0.22) | | 5.15E-77 | | 1.58 (1.54, 1.62) | | 0.38 (0.35, 0.42) | | 0.29 (0.26, 0.32) | | 0.15 (0.12, 0.18) | | -0.07 (-0.09, -0.05) |
|  |  |  | tertile 1 | |  | | 119.00 (7.60) | | ref. | |  | | ref. | | ref. | | ref. | | ref. | | ref. |
|  |  |  | tertile 2 | |  | | 136.79 (4.42) | | 0.22 (0.17, 0.27) | | 2.98E-19 | | 1.54 (1.45, 1.63) | | 0.49 (0.40, 0.58) | | 0.44 (0.38, 0.50) | | 0.15 (0.07, 0.22) | | -0.23(-0.27, -0.18) |
|  |  |  | tertile 3 | |  | | 158.94 (12.27) | | 0.47 (0.42, 0.52) | | 9.22E-76 | | 3.60 (3.51, 3.70) | | 0.90 (0.81, 0.99) | | 0.69 (0.63, 0.76) | | 0.38 (0.31, 0.46) | | -0.14(-0.18, -0.09) |
|  | Derived | Hand grip strength | 1-point | | 239,034 | | 31.08 (11.08) | | -0.74 (-0.77, -0.71) | | 0.00E+00 | | -2.08 (-2.14, -2.03) | | -1.44 (-1.49, -1.39) | | -0.42 (-0.45, -0.39) | | -0.57 (-0.60, -0.54) | | -0.74(-0.77, -0.71) |
|  |  |  | tertile 1 | |  | | 19.76 (4.24) | | ref. | |  | | ref. | | ref. | | ref. | | ref. | | ref. |
|  |  |  | tertile 2 | |  | | 30.42 (3.14) | | -0.45 (-0.50, -0.40) | | 5.62E-66 | | -2.25 (-2.34, -2.15) | | -1.23 (-1.32, -1.14) | | -0.65 (-0.71, -0.59) | | -0.83 (-0.90, -0.76) | | -0.37 (-0.41, -0.32) |
|  |  |  | tertile 3 | |  | | 44.45 (5.97) | | -1.36 (-1.43, -1.29) | | 4.46E-308 | | -4.07 (-4.20, -3.93) | | -2.84 (-2.97, -2.72) | | -0.74 (-0.81, -0.68) | | -1.10 (-1.17, -1.03) | | -1.36 (-1.43, -1.29) |
| **lifestyle** | Derived | Fruit eaten above recommendation | yes vs. no (ref.) | | 239,291 | | 119475 (49.93%) | | -1.03 (-1.07, -1.00) | | 0.00E+00 | | -0.01 (-0.09, 0.06) | | -0.90 (-0.97, -0.83) | | -1.02 (-1.07, -0.97) | | -1.00 (-1.06, -0.94) | | -0.93 (-0.96, -0.89) |
|  | Derived | Fish eaten above recommendation | yes vs. no (ref.) | | 239,291 | | 184416 (77.07%) | | -1.39 (-1.44, -1.35) | | 0.00E+00 | | -0.64 (-0.73, -0.56) | | -1.02 (-1.11, -0.93) | | -1.40 (-1.46, -1.34) | | -1.38 (-1.44, -1.31) | | -1.33 (-1.38, -1.29) |
|  | Derived | Processed meat intake reduced | yes vs. no (ref.) | | 238,936 | | 92712 (38.80%) | | -0.64 (-0.68, -0.60) | | 6.91E-216 | | -0.10 (-0.18, -0.02) | | -0.80 (-0.88, -0.73) | | -0.54 (-0.59, -0.49) | | -0.80 (-0.86, -0.73) | | -0.36 (-0.40, -0.32) |
|  | Derived | Red meat intake reduced | yes vs. no (ref.) | | 239,291 | | 104210 (43.55%) | | -0.41 (-0.45, -0.37) | | 1.67E-95 | | -0.61 (-0.68, -0.53) | | -0.67 (-0.74, -0.60) | | -0.30 (-0.35, -0.25) | | -0.54 (-0.60, -0.48) | | -0.18 (-0.22, -0.15) |
|  | Derived | Wholegrain eaten above recommendation | yes vs. no (ref.) | | 217,985 | | 134845 (61.86%) | | -1.36 (-1.40, -1.32) | | 0.00E+00 | | -0.59 (-0.66, -0.51) | | -1.46 (-1.53, -1.38) | | -1.14 (-1.20, -1.09) | | -1.57 (-1.63, -1.51) | | -1.17 (-1.21, -1.13) |
|  | Derived | White bread/refined cereals intake reduced | yes vs. no (ref.) | | 209,465 | | 3852 (1.84%) | | -0.22 (-0.37, -0.07) | | 0.004 | | -0.68 (-0.95, -0.41) | | -0.62 (-0.93, -0.30) | | -0.11 (-0.31, 0.09) | | -0.37 (-0.60, -0.14) | | -0.21 (-0.35, -0.07) |
|  | Derived | Vegetables eaten above recommendation | yes vs. no (ref.) | | 239,291 | | 194848 (81.43%) | | -1.22 (-1.27, -1.17) | | 0.00E+00 | | -0.44 (-0.54, -0.35) | | -1.22 (-1.32, -1.13) | | -1.31 (-1.38, -1.24) | | -1.19 (-1.26, -1.12) | | -1.16 (-1.21, -1.11) |
|  | Derived | Healthy diet | yes vs. no (ref.) | | 239,291 | | 149820 (62.61%) | | -1.52 (-1.56, -1.48) | | 0.00E+00 | | -0.67 (-0.75, -0.59) | | -1.55 (-1.62, -1.47) | | -1.39 (-1.45, -1.34) | | -1.65 (-1.70, -1.59) | | -1.29 (-1.32, -1.25) |
|  | Derived | Diet index | 1-point | | 239,291 | | 4.04 (1.70) | | -0.90 (-0.92, -0.88) | | 0.00E+00 | | -0.37 (-0.41, -0.34) | | -0.89 (-0.93, -0.86) | | -0.78 (-0.81, -0.76) | | -0.99 (-1.02, -0.96) | | -0.76 (-0.78 ,-0.74) |
|  |  |  | tertile 1 | |  | | 2.19 (0.85) | | ref. | |  | | ref. | | ref. | | ref. | | ref. | | ref. |
|  |  |  | tertile 2 | |  | | 4.52 (0.50) | | -1.27 (-1.31, -1.22) | | 0.00E+00 | | -0.62 (-0.71, -0.54) | | -1.25 (-1.33, -1.17) | | -0.99 (-1.05, -0.93) | | -1.39 (-1.45, -1.33) | | -1.10 (-1.14, -1.06) |
|  |  |  | tertile 3 | |  | | 6.31 (0.46) | | -2.04 (-2.09, -1.98) | | 0.00E+00 | | -0.77 (-0.87, -0.67) | | -2.11 (-2.20, -2.01) | | -1.45 (-1.51, -1.39) | | -2.31 (-2.39, -2.22) | | -1.66 (-1.71, -1.62) |
|  | 1488 | Tea intake | 1-point | | 238,903 | | 3.46 (2.88) | | 0.09 (0.07, 0.10) | | 1.96E-18 | | 0.36 (0.32, 0.40) | | 0.12 (0.09, 0.16) | | 0.06 (0.03, 0.08) | | 0.11 (0.08, 0.14) | | 0.13 (0.11, 0.14) |
|  |  |  | tertile 1 | |  | | 0.94 (0.86) | | ref. | |  | | ref. | | ref. | | ref. | | ref. | | ref. |
|  |  |  | tertile 2 | |  | | 3.49 (0.50) | | -0.40 (-0.45, -0.36) | | 5.80E-65 | | 0.27 (0.18, 0.36) | | -0.25 (-0.33, -0.16) | | -0.41 (-0.47, -0.35) | | -0.41 (-0.48, -0.34) | | -0.25 (-0.29, -0.20) |
|  |  |  | tertile 3 | |  | | 6.69 (2.67) | | 0.11 (0.06, 0.15) | | 2.83E-06 | | 0.85 (0.76, 0.94) | | 0.24 (0.15, 0.32) | | 0.07 (0.01, 0.13) | | 0.14 (0.07, 0.21) | | 0.20 (0.16, 0.24) |
|  | 1498 | Coffee intake | 1-point | | 238,882 | | 2.04 (2.10) | | 0.20 (0.18, 0.22) | | 1.94E-96 | | 0.25 (0.21, 0.29) | | 0.09 (0.05, 0.12) | | 0.21 (0.18, 0.23) | | 0.22 (0.20, 0.25) | | 0.13 (0.11, 0.15) |
|  |  |  | tertile 1 | |  | | 0.48 (0.46) | | ref. | |  | | ref. | | ref. | | ref. | | ref. | | ref. |
|  |  |  | tertile 2 | |  | | 2.00 (0.00) | | -0.50 (-0.55, -0.45) | | 3.48E-79 | | -0.14 (-0.24, -0.04) | | -0.57 (-0.66, -0.48) | | -0.45 (-0.52, -0.38) | | -0.45 (-0.52, -0.38) | | -0.46 (-0.51, -0.41) |
|  |  |  | tertile 3 | |  | | 4.44 (1.98) | | 0.25 (0.21, 0.29) | | 5.35E-30 | | 0.43 (0.34, 0.51) | | 0.02 (-0.06, 0.10) | | 0.35 (0.29, 0.40) | | 0.51 (0.44, 0.59) | | 0.09 (0.05, 0.13) |
|  | 1528 | Water intake | 1-point | | 237,800 | | 2.67 (2.23) | | -0.12 (-0.14, -0.10) | | 1.45E-32 | | -0.56 (-0.59, -0.52) | | -0.11 (-0.14, -0.07) | | -0.14 (-0.17, -0.12) | | -0.06 (-0.09, -0.04) | | -0.19 (-0.21, -0.17) |
|  |  |  | tertile 1 | |  | | 0.65 (0.42) | | ref. | |  | | ref. | | ref. | | ref. | | ref. | | ref. |
|  |  |  | tertile 2 | |  | | 2.40 (0.49) | | -0.30 (-0.35, -0.26) | | 1.39E-39 | | -0.55 (-0.65, -0.44) | | -0.15 (-0.23, -0.07) | | -0.30 (-0.37, -0.23) | | -0.24 (-0.31, -0.18) | | -0.42 (-0.47, -0.38) |
|  |  |  | tertile 3 | |  | | 5.48 (2.07) | | -0.39 (-0.44, -0.34) | | 1.00E-52 | | -1.17 (-1.26, -1.09) | | -0.28 (-0.38, -0.19) | | -0.34 (-0.40, -0.29) | | -0.23 (-0.31, -0.15) | | -0.58 (-0.63, -0.54) |
|  | 6155 | Vitamin A | yes vs. no (ref.) | | 239,290 | | 2428 (1.91%) | | -0.80 (-0.93, -0.66) | | 1.15E-29 | | -0.18 (-0.47, 0.12) | | -0.52 (-0.75, -0.29) | | -0.94 (-1.12, -0.76) | | -0.64 (-0.85, -0.43) | | -0.66 (-0.79, -0.53) |
|  |  | Vitamin B | yes vs. no (ref.) | | 239,290 | | 6457 (5.09%) | | -0.30 (-0.39, -0.20) | | 8.19E-10 | | -0.04 (-0.23, 0.14) | | -0.35 (-0.53, -0.18) | | -0.61 (-0.72, -0.49) | | 0.22 (0.05, 0.38) | | -0.18 (-0.27, -0.09) |
|  |  | Vitamin C | yes vs. no (ref.) | | 239,290 | | 11065 (8.72%) | | -0.73 (-0.80, -0.67) | | 4.59E-100 | | -0.43 (-0.56, -0.29) | | -0.70 (-0.82, -0.58) | | -0.76 (-0.85, -0.67) | | -0.70 (-0.81, -0.60) | | -0.58 (-0.65, -0.52) |
|  |  | Vitamin D | yes vs. no (ref.) | | 239,290 | | 6391 (5.04%) | | -0.87 (-0.97, -0.77) | | 9.90E-66 | | 0.43 (0.21, 0.64) | | -0.66 (-0.82, -0.50) | | -0.88 (-1.00, -0.77) | | -0.69 (-0.88, -0.51) | | -0.59 (-0.68, -0.49) |
|  |  | Vitamin E | yes vs. no (ref.) | | 239,290 | | 4328 (3.41%) | | -1.28 (-1.39, -1.16) | | 1.29E-108 | | -0.23 (-0.46, 0.01) | | -1.24 (-1.43, -1.05) | | -1.37 (-1.51, -1.24) | | -1.10 (-1.29, -0.91) | | -1.10 (-1.21, -0.99) |
|  |  | Folic acid or Folate (Vit B9) | yes vs. no (ref.) | | 239,290 | | 3491 (2.75%) | | 0.88 (0.75, 1.02) | | 3.31E-40 | | 0.51 (0.25, 0.77) | | 1.19 (0.96, 1.41) | | 0.73 (0.58, 0.88) | | 1.09 (0.85, 1.33) | | 0.88 (0.76, 1.00) |
|  |  | Multivitamins +/- minerals | yes vs. no (ref.) | | 239,290 | | 31036 (24.47%) | | -0.77 (-0.82, -0.72) | | 1.03E-233 | | -0.60 (-0.69, -0.51) | | -0.96 (-1.05, -0.88) | | -0.79 (-0.85, -0.73) | | -0.74 (-0.81, -0.67) | | -0.68 (-0.72,- 0.63) |
|  | 6179 | Fish oil (including cod liver oil) | yes vs. no (ref.) | | 239,290 | | 41393 (32.64%) | | -1.41 (-1.46, -1.37) | | 0.00E+00 | | 0.32 (0.24, 0.41) | | -1.29 (-1.36, -1.22) | | -1.44 (-1.50, -1.39) | | -1.35 (-1.41, -1.29) | | -1.28 (-1.32, -1.24) |
|  |  | Glucosamine | yes vs. no (ref.) | | 239,290 | | 27825 (21.94%) | | -1.30 (-1.35, -1.25) | | 0.00E+00 | | 0.82 (0.72, 0.93) | | -1.27 (-1.35, -1.19) | | -1.16 (-1.22, -1.10) | | -1.41 (-1.49, -1.33) | | -1.27 (-1.31, -1.22) |
|  |  | Calcium | yes vs. no (ref.) | | 239,290 | | 13047 (10.29%) | | -0.81 (-0.89, -0.73) | | 4.52E-94 | | 0.87 (0.71, 1.04) | | -0.59 (-0.72, -0.46) | | -0.85 (-0.93, -0.76) | | -0.29 (-0.47, -0.11) | | -0.46 (-0.53, -0.39) |
|  |  | Zinc | yes vs. no (ref.) | | 239,290 | | 5968 (4.71%) | | -1.04 (-1.13, -0.94) | | 1.34E-98 | | -1.00 (-1.19, -0.81) | | -1.15 (-1.32, -0.98) | | -1.01 (-1.12, -0.89) | | -1.07 (-1.23, -0.91) | | -0.86 (-0.95, -0.76) |
|  |  | Iron | yes vs. no (ref.) | | 239,290 | | 4561 (3.60%) | | 0.52 (0.41, 0.64) | | 8.64E-19 | | -1.01 (-1.21, -0.81) | | 0.92 (0.67, 1.17) | | 0.23 (0.09, 0.36) | | 0.85 (0.63, 1.06) | | 0.62 (0.51, 0.73) |
|  |  | Selenium | yes vs. no (ref.) | | 239,290 | | 2672 (2.11%) | | -1.21 (-1.35, -1.07) | | 2.81E-67 | | -0.15 (-0.44, 0.14) | | -1.17 (-1.40, -0.94) | | -1.12 (-1.29, -0.95) | | -1.33 (-1.55, -1.12) | | -1.05 (-1.18, -0.92) |
|  | 1190 | Nap during day |  | | 239,055 | |  | | *Global P* | | 0.00E+00 | |  | |  | |  | |  | |  |
|  |  |  | Never/rarely | |  | | 133856 (55.99%) | | ref. | |  | | ref. | | ref. | | ref. | | ref. | | ref. |
|  |  |  | Sometimes | |  | | 92305 (38.61%) | | 0.91 (0.87, 0.95) | | 0.00E+00 | | 1.70 (1.62, 1.77) | | 1.35 (1.28, 1.42) | | 0.83 (0.77, 0.88) | | 0.94 (0.88, 1.01) | | 0.62 (0.58, 0.65) |
|  |  |  | Usually | |  | | 12894 (5.39%) | | 1.80 (1.71, 1.88) | | 0.00E+00 | | 3.23 (3.04, 3.42) | | 2.59 (2.45, 2.72) | | 1.85 (1.72, 1.99) | | 1.67 (1.56, 1.78) | | 1.32 (1.24, 1.40) |
|  | 1200 | Sleeplessness / insomnia |  | | 239,144 | |  | | *Global P* | | 0.00E+00 | |  | |  | |  | |  | |  |
|  |  |  | Never/rarely | |  | | 57776 (24.16%) | | ref. | |  | | ref. | | ref. | | ref. | | ref. | | ref. |
|  |  |  | Sometimes | |  | | 114280 (47.79%) | | 0.19 (0.14, 0.23) | | 2.07E-14 | | 1.09 (1.00, 1.17) | | 0.29 (0.20, 0.38) | | 0.05 (-0.02, 0.12) | | 0.33 (0.26, 0.40) | | 0.12 (0.08, 0.17) |
|  |  |  | Usually | |  | | 67088 (28.05%) | | 0.68 (0.62, 0.73) | | 1.82E-135 | | 2.40 (2.30, 2.50) | | 0.76 (0.66, 0.86) | | 0.50 (0.42, 0.57) | | 0.89 (0.81, 0.97) | | 0.43 (0.38, 0.48) |
|  | 1210 | Snoring | yes (ref.) vs. no | | 222,789 | | 138623 (62.22%) | | -0.61 (-0.65, -0.57) | | 6.33E-187 | | -1.59 (-1.67, -1.52) | | -0.04 (-0.12, 0.03) | | -0.85 (-0.90, -0.79) | | -0.42 (-0.48, -0.36) | | -0.01 (-0.05, 0.03) |
|  | 1220 | Daytime dozing / sleeping (narcolepsy) |  | | 238,386 | |  | | *Global P* | | 0.00E+00 | |  | |  | |  | |  | |  |
|  |  |  | Never/rarely | |  | | 181565 (76.16%) | | ref. | |  | | ref. | | ref. | | ref. | | ref. | | ref. |
|  |  |  | Sometimes | |  | | 50356 (21.12%) | | 0.56 (0.51, 0.61) | | 1.05E-120 | | 1.53 (1.44, 1.63) | | 0.97 (0.89, 1.05) | | 0.63 (0.56, 0.69) | | 0.49 (0.42, 0.56) | | 0.34 (0.30, 0.39) |
|  |  |  | Often | |  | | 6445 (2.70%) | | 1.63 (1.51, 1.75) | | 7.00E-163 | | 2.65 (2.41, 2.89) | | 2.01 (1.81, 2.21) | | 1.64 (1.48, 1.80) | | 1.57 (1.40, 1.74) | | 1.10 (0.99, 1.21) |
|  |  |  | All of the time | |  | | 20 (0.01%) | | 1.74 (-0.33, 3.81) | | 0.099 | | 3.34 (-0.91, 7.59) | | 2.23 (-1.30, 5.76) | | 1.37 (-1.18, 3.92) | | 2.13 (-1.29, 5.55) | | 1.14 (-0.83, 3.10) |
|  | Derived | Moderate sleep | yes (ref.) vs. no | | 238,184 | | 62311 (26.16%) | | 0.67 (0.63, 0.71) | | 1.22E-201 | | 1.30 (1.22, 1.39) | | 0.52 (0.44, 0.60) | | 0.63 (0.57, 0.69) | | 0.80 (0.73, 0.86) | | 0.41 (0.37, 0.45) |
|  | 22037 | MET minutes per week for walking | 1-point | | 195,962 | | 1046.14 (1091.18) | | -0.09 (-0.11, -0.07) | | 2.02E-18 | | -0.03 (-0.07, 0.01) | | -0.20 (-0.24, -0.16) | | -0.13 (-0.15, -0.10) | | -0.06 (-0.09, -0.03) | | -0.01 (-0.03, 0.01) |
|  |  |  | tertile 1 | |  | | 208.21 (124.98) | | ref. | |  | | ref. | | ref. | | ref. | | ref. | | ref. |
|  |  |  | tertile 2 | |  | | 689.49 (185.46) | | -0.45 (-0.50, -0.40) | | 4.40E-69 | | -0.34 (-0.44, -0.24) | | -0.54 (-0.64, -0.45) | | -0.52 (-0.59, -0.45) | | -0.40 (-0.48, -0.33) | | -0.23 (-0.28, -0.19) |
|  |  |  | tertile 3 | |  | | 2323.13 (1065.39) | | -0.42 (-0.48, -0.37) | | 8.99E-60 | | -0.18 (-0.28, -0.09) | | -0.56 (-0.66, -0.47) | | -0.52 (-0.59, -0.45) | | -0.34 (-0.41, -0.26) | | -0.15 (-0.20, -0.10) |
|  | 22038 | MET minutes per week for moderate activity | 1-point | | 195,962 | | 937.88 (1230.59) | | -0.24 (-0.26, -0.22) | | 5.68E-111 | | -0.05 (-0.09, -0.01) | | -0.20 (-0.24, -0.16) | | -0.24 (-0.27, -0.22) | | -0.22 (-0.25, -0.19) | | -0.13 (-0.15, -0.11) |
|  |  |  | tertile 1 | |  | | 90.30 (92.67) | | ref. | |  | | ref. | | ref. | | ref. | | ref. | | ref. |
|  |  |  | tertile 2 | |  | | 539.17 (169.77) | | -0.79 (-0.84, -0.74) | | 8.09E-202 | | -0.83 (-0.92, -0.73) | | -0.78 (-0.87, -0.68) | | -0.72 (-0.79, -0.65) | | -0.87 (-0.95, -0.79) | | -0.54 (-0.59, -0.49) |
|  |  |  | tertile 3 | |  | | 2330.58 (1312.95) | | -0.85 (-0.90, -0.80) | | 2.01E-245 | | -0.60 (-0.70, -0.50) | | -0.75 (-0.85, -0.66) | | -0.85 (-0.91, -0.78) | | -0.82 (-0.89, -0.74) | | -0.53 (-0.58, -0.48) |
|  | 22039 | MET minutes per week for vigorous activity | 1-point | | 195,962 | | 679.24 (1215.25) | | -0.36 (-0.38, -0.34) | | 1.69E-244 | | -0.63 (-0.67, -0.59) | | -0.40 (-0.44, -0.37) | | -0.31 (-0.33, -0.28) | | -0.38 (-0.41, -0.35) | | -0.25 (-0.27, -0.23) |
|  |  |  | tertile 1 | |  | | 0.00 (0.00) | | ref. | |  | | ref. | | ref. | | ref. | | ref. | | ref. |
|  |  |  | tertile 2 | |  | | 288.11 (146.77) | | -1.33 (-1.39, -1.28) | | 0.00E+00 | | -1.74 (-1.84, -1.64) | | -1.68 (-1.78, -1.59) | | -1.19 (-1.26, -1.12) | | -1.57 (-1.64, -1.49) | | -1.02 (-1.07, -0.97) |
|  |  |  | tertile 3 | |  | | 1828.24 (1563.10) | | -1.70 (-1.75, -1.66) | | 0.00E+00 | | -2.77 (-2.86, -2.67) | | -1.92 (-2.01, -1.83) | | -1.37 (-1.44, -1.31) | | -2.01 (-2.08, -1.93) | | -1.30 (-1.34, -1.25) |
|  | 22040 | Summed MET minutes per week for all activity | 1-point | | 195,962 | | 2663.26 (2729.44) | | -0.30 (-0.32, -0.28) | | 4.09E-177 | | -0.31 (-0.35, -0.27) | | -0.35 (-0.39, -0.31) | | -0.30 (-0.32, -0.27) | | -0.29 (-0.32, -0.26) | | -0.17 (-0.19, -0.15) |
|  |  |  | tertile 1 | |  | | 538.90 (313.28) | | ref. | |  | | ref. | | ref. | | ref. | | ref. | | ref. |
|  |  |  | tertile 2 | |  | | 1827.23 (481.13) | | -1.03 (-1.08, -0.98) | | 0.00E+00 | | -1.22 (-1.31, -1.12) | | -1.01 (-1.11, -0.92) | | -0.86 (-0.93, -0.79) | | -1.21 (-1.29, -1.14) | | -0.71 (-0.76, -0.66) |
|  |  |  | tertile 3 | |  | | 5624.97 (2835.66) | | -1.13 (-1.18, -1.08) | | 0.00E+00 | | -1.28 (-1.38, -1.18) | | -1.09 (-1.19, -1.00) | | -1.00 (-1.07, -0.93) | | -1.22 (-1.30, -1.15) | | -0.73 (-0.78, -0.68) |
|  | 22036 | Above moderate/vigorous/walking recommendation | yes (ref.) vs. no | | 195,922 | | 159695 (81.51%) | | -1.18 (-1.24, -1.13) | | 0.00E+00 | | -1.19 (-1.29, -1.09) | | -1.24 (-1.34, -1.13) | | -1.08 (-1.15, -1.00) | | -1.28 (-1.36, -1.20) | | -0.74 (-0.79, -0.69) |
|  | Derived | Leisure activity | yes vs. no (ref.) | | 238,732 | | 166878 (69.90%) | | -0.81 (-0.86, -0.77) | | 0.00E+00 | | -0.70 (-0.78, -0.62) | | -0.66 (-0.74, -0.59) | | -0.95 (-1.01, -0.90) | | -0.63 (-0.69, -0.57) | | -0.65 (-0.69, -0.61) |
|  | 1558 | Alcohol intake frequency |  | | 239,291 | |  | | *Global P* | | 0.00E+00 | |  | |  | |  | |  | |  |
|  |  |  | never or special occasions only | |  | | 30651 (24.17%) | | ref. | |  | | ref. | | ref. | | ref. | | ref. | | ref. |
|  |  |  | one to three times per month | |  | | 17097 (13.48%) | | -0.77 (-0.84, -0.70) | | 1.19E-99 | | -1.46 (-1.60, -1.32) | | -1.26 (-1.40, -1.13) | | -0.82 (-0.91, -0.74) | | -0.75 (-0.87, -0.62) | | -0.66 (-0.73, -0.59) |
|  |  |  | one to four times per week | |  | | 59438 (46.87%) | | -1.73 (-1.78, -1.68) | | 0.00E+00 | | -2.01 (-2.11, -1.90) | | -2.19 (-2.28, -2.10) | | -1.73 (-1.79, -1.67) | | -1.80 (-1.89, -1.71) | | -1.36 (-1.41, -1.31) |
|  |  |  | daily or almost daily | |  | | 19642 (15.49%) | | -2.02 (-2.08, -1.96) | | 0.00E+00 | | -1.19 (-1.31, -1.06) | | -2.61 (-2.72, -2.51) | | -2.02 (-2.10, -1.94) | | -2.13 (-2.23, -2.03) | | -1.47 (-1.53, -1.41) |
|  | 20116 | Smoking status |  | | 239,291 | |  | | *Global P* | | 0.00E+00 | |  | |  | |  | |  | |  |
|  |  |  | Never | |  | | 75720 (59.70%) | | ref. | |  | | ref. | | ref. | | ref. | | ref. | | ref. |
|  |  |  | Previous | |  | | 39848 (31.42%) | | 0.61 (0.57, 0.65) | | 1.03E-185 | | 1.39 (1.31, 1.47) | | 0.95 (0.87, 1.02) | | 0.35 (0.29, 0.40) | | 0.82 (0.76, 0.88) | | 0.39 (0.35, 0.43) |
|  |  |  | Curent | |  | | 11260 (8.88%) | | 2.87 (2.80, 2.93) | | 0.00E+00 | | 2.24 (2.12, 2.35) | | 2.85 (2.72, 2.98) | | 2.53 (2.44, 2.62) | | 3.18 (3.09, 3.27) | | 2.92 (2.86, 2.98) |
|  | 1269 | Exposure to tobacco smoke at home | yes vs. no (ref.) | | 216,258 | | 12096 (5.59%) | | 1.34 (1.25, 1.42) | | 1.22E-209 | | 1.32 (1.16, 1.48) | | 1.26 (1.10, 1.42) | | 1.25 (1.14, 1.36) | | 1.43 (1.30, 1.56) | | 0.86 (0.78, 0.94) |
|  | 1279 | Exposure to tobacco smoke outside home | yes vs. no (ref.) | | 202,681 | | 39477 (19.48%) | | 0.74 (0.69, 0.79) | | 1.42E-178 | | 0.23 (0.13, 0.33) | | 0.56 (0.46, 0.66) | | 0.71 (0.64, 0.78) | | 0.77 (0.69, 0.84) | | 0.40 (0.36, 0.45) |
|  | 1120 | Weekly usage of mobile phone in last 3 months |  | | 201,004 | |  | | *Global P* | | 0.00E+00 | |  | |  | |  | |  | |  |
|  |  |  | Less than 5mins | |  | | 42957 (21.37%) | | ref. | |  | | ref. | | ref. | | ref. | | ref. | | ref. |
|  |  |  | 5-29 mins | |  | | 78391 (39.00%) | | 0.13 (0.07, 0.18) | | 8.58E-06 | | -0.51 (-0.62, -0.39) | | -0.19 (-0.28, -0.09) | | 0.17 (0.10, 0.24) | | 0.10 (0.02, 0.19) | | 0.03 (-0.03, 0.08) |
|  |  |  | 30-59 mins | |  | | 34366 (17.10%) | | 0.23 (0.16, 0.30) | | 1.71E-11 | | -0.91 (-1.04, -0.78) | | -0.39 (-0.52, -0.27) | | 0.31 (0.22, 0.40) | | 0.18 (0.08, 0.29) | | 0.02 (-0.04, 0.08) |
|  |  |  | 1-3 hours | |  | | 28461 (14.16%) | | 0.31 (0.24, 0.39) | | 1.47E-17 | | -1.54 (-1.68, -1.41) | | -0.52 (-0.67, -0.37) | | 0.43 (0.33, 0.52) | | 0.27 (0.16, 0.38) | | 0.03 (-0.04, 0.10) |
|  |  |  | 4-6 hours | |  | | 8282 (4.12%) | | 0.42 (0.31, 0.54) | | 1.25E-13 | | -2.00 (-2.19, -1.80) | | -0.55 (-0.83, -0.26) | | 0.73 (0.57, 0.89) | | 0.26 (0.09, 0.42) | | 0.00 (-0.11, 0.10) |
|  |  |  | More than 6 hours | |  | | 8547 (4.25%) | | 0.70 (0.59, 0.81) | | 1.03E-34 | | -2.17 (-2.36, -1.99) | | -0.34 (-0.67, -0.02) | | 1.03 (0.87, 1.19) | | 0.59 (0.43, 0.75) | | 0.18 (0.07, 0.28) |
|  | 1130 | Hands-free device/speakerphone use with mobile phone in last 3 month |  | | 201,710 | |  | | *Global P* | | 0.00E+00 | |  | |  | |  | |  | |  |
|  |  |  | Never or almost never | |  | | 165614 (82.11%) | | ref. | |  | | ref. | | ref. | | ref. | | ref. | | ref. |
|  |  |  | Less than half the time | |  | | 19092 (9.47%) | | -0.35 (-0.42, -0.28) | | 1.42E-21 | | -1.56 (-1.68, -1.44) | | -1.06 (-1.23, -0.90) | | -0.29 (-0.40, -0.17) | | -0.34 (-0.43, -0.24) | | -0.49 (-0.56, -0.42) |
|  |  |  | About half the time | |  | | 7482 (3.71%) | | -0.25 (-0.36, -0.14) | | 1.02E-05 | | -1.71 (-1.90, -1.53) | | -0.85 (-1.12, -0.57) | | -0.25 (-0.43, -0.06) | | -0.18 (-0.32, -0.04) | | -0.50 (-0.60, -0.40) |
|  |  |  | More than half the time | |  | | 4132 (2.05%) | | -0.05 (-0.19, 0.10) | | 5.41E-01 | | -1.44 (-1.68, -1.20) | | -0.96 (-1.34, -0.57) | | -0.21 (-0.47, 0.06) | | 0.10 (-0.08, 0.28) | | -0.38 (-0.52, -0.24) |
|  |  |  | Always or almost always | |  | | 5390 (2.67%) | | 0.40 (0.27, 0.53) | | 7.33E-10 | | -0.73 (-0.95, -0.50) | | -0.33 (-0.61, -0.04) | | 0.14 (-0.08, 0.35) | | 0.60 (0.43, 0.76) | | -0.01 (-0.13, 0.11) |
|  | 1050 | Time spend outdoors in summer | 1-point | | 226,682 | | 3.81 (2.39) | | 0.17 (0.15, 0.19) | | 1.26E-59 | | 0.36 (0.32, 0.40) | | 0.27 (0.24, 0.31) | | 0.07 (0.05, 0.10) | | 0.23 (0.20, 0.26) | | 0.12 (0.11, 0.14) |
|  |  |  | tertile 1 | |  | | 1.55 (0.59) | | ref. | |  | | ref. | | ref. | | ref. | | ref. | | ref. |
|  |  |  | tertile 2 | |  | | 3.49 (0.50) | | 0.06 (0.02, 0.11) | | 9.42E-03 | | 0.44 (0.35, 0.52) | | 0.51 (0.43, 0.59) | | 0.01 (-0.05, 0.07) | | 0.32 (0.24, 0.39) | | 0.05 (0.01, 0.10) |
|  |  |  | tertile 3 | |  | | 6.63 (1.92) | | 0.37 (0.32, 0.42) | | 4.97E-50 | | 1.02 (0.92, 1.11) | | 0.74 (0.65, 0.83) | | 0.19 (0.13, 0.26) | | 0.57 (0.49, 0.64) | | 0.29 (0.24, 0.34) |
|  | 1060 | Time spent outdoors in winter | 1-point | | 226,554 | | 1.94 (1.80) | | 0.14 (0.12, 0.16) | | 1.42E-41 | | 0.23 (0.19, 0.27) | | 0.21 (0.17, 0.24) | | 0.07 (0.05, 0.10) | | 0.19 (0.16, 0.22) | | 0.10 (0.08, 0.11) |
|  |  |  | tertile 1 | |  | | 0.78 (0.30) | | ref. | |  | | ref. | | ref. | | ref. | | ref. | | ref. |
|  |  |  | tertile 2 | |  | | 2.00 (0.00) | | -0.03 (-0.08, 0.02) | | 2.62E-01 | | 0.32 (0.23, 0.42) | | 0.22 (0.13, 0.30) | | -0.02 (-0.08, 0.04) | | -0.03 (-0.10, 0.05) | | 0.00 (-0.05, 0.04) |
|  |  |  | tertile 3 | |  | | 4.54 (2.00) | | 0.32 (0.27, 0.37) | | 4.20E-36 | | 0.85 (0.75, 0.95) | | 0.61 (0.52, 0.69) | | 0.26 (0.19, 0.33) | | 0.36 (0.29, 0.43) | | 0.24 (0.19, 0.28) |
|  | 1070 | Time spent watching television (TV) | 1-point | | 237,759 | | 2.81 (1.64) | | 0.90 (0.88, 0.92) | | 0.00E+00 | | 1.39 (1.36, 1.43) | | 1.13 (1.10, 1.16) | | 0.84 (0.81, 0.86) | | 0.98 (0.95, 1.01) | | 0.61 (0.59, 0.63) |
|  |  |  | tertile 1 | |  | | 1.46 (0.67) | | ref. | |  | | ref. | | ref. | | ref. | | ref. | | ref. |
|  |  |  | tertile 2 | |  | | 3.00 (0.00) | | 0.73 (0.68, 0.77) | | 3.60E-196 | | 1.55 (1.46, 1.64) | | 1.26 (1.18, 1.33) | | 0.70 (0.64, 0.76) | | 0.79 (0.72, 0.86) | | 0.40 (0.36, 0.45) |
|  |  |  | tertile 3 | |  | | 4.78 (1.28) | | 1.83 (1.79, 1.88) | | 0.00E+00 | | 3.32 (3.23, 3.41) | | 2.97 (2.87, 3.07) | | 1.75 (1.69, 1.81) | | 1.97 (1.90, 2.04) | | 1.21 (1.16, 1.25) |
|  | 1080 | Time spent using computer | 1-point | | 237,626 | | 1.07 (1.37) | | 0.07 (0.05, 0.09) | | 1.14E-11 | | -0.10 (-0.14, -0.07) | | -0.19 (-0.23, -0.16) | | 0.09 (0.06, 0.11) | | 0.04 (0.01, 0.07) | | -0.03 (-0.05, -0.02) |
|  |  |  | tertile 1 | |  | | 0.22 (0.25) | | ref. | |  | | ref. | | ref. | | ref. | | ref. | | ref. |
|  |  |  | tertile 2 | |  | | 1.00 (0.00) | | -0.44 (-0.48, -0.39) | | 3.44E-81 | | -0.55 (-0.64, -0.47) | | -2.04 (-2.12, -1.96) | | -0.31 (-0.37, -0.26) | | -0.60 (-0.67, -0.54) | | -0.46 (-0.50, -0.42) |
|  |  |  | tertile 3 | |  | | 3.04 (1.66) | | 0.26 (0.21, 0.31) | | 2.40E-24 | | 0.09 (-0.01, 0.19) | | -1.03 (-1.13, -0.94) | | 0.45 (0.38, 0.51) | | 0.04 (-0.03, 0.11) | | -0.05 (-0.09, 0.00) |

Notes: Model 1 was adjusted for chronological age, sex, and ethnicity. Model 2 was further adjusted for body mass index based on Model 1. N, available sample size for each factor; Values are shown as mean (SD) for continuous variables and as frequencies (%) for categorical variables. Ref. indicates the reference category for categorical variables. For categorical variables with more than two categories a Global P has been estimated using a likelihood ratio test and P value given in the P value column. Note that the Bonferroni corrected P value for the number of tests carried out is 4.67x10^-4^. Factors with P value < 4.67x10^-4^ are highlighted in dark green. Factors with P value > 4.67x10^-4^ but < 0.05 are highlighted in light green.

Table S34. ICD codes used in the UK Biobank to define all-cause and cause-specific mortality.

| **Mortality category** | | **Field IDs & ICD codes** |
| --- | --- | --- |
| All-cause mortality | - | 40000 |
| Cause-specific mortality | Cancer | ICD10: 40001 (C00-C97) |
|  | Cardiovascular disease | ICD10: 40001 (I00-I99) |
|  | Respiratory disease | ICD10: 40001 (J09-J98) |
|  | Digestive disease | ICD10: 40001 (K20-K93) |
|  | Neurodegenerative disease | ICD10: 40001 (F01-03, G122, G20, G21, G231-233, G238, G239, G30, G31) |
|  | Other causes ^a^ | - |

^a^ Other causes mortality refers to death from causes other than cancer, cardiovascular disease, respiratory disease, digestive disease, and neurodegenerative disease.

Table S35. ICD codes used in the UK Biobank to define the common health-related outcomes.

| **Diseases** | **Field IDs & ICD codes** |
| --- | --- |
| Cancer | ICD9: 40013 (140-208, except 173)  ICD10: 40006 (C00-C96, except C44)  Self-report: 20001 (1001-1088) |
| Cardiovascular disease | ICD9: 41271 (410.x, 411.x, 412.x, 413.x, 414.x,429.79, 430.x, 431.x, 432.x, 433.x, 434.x, 435.x, 436.x, 437.x, 438.x)  ICD10: 41270 (I20.x, I21.x, I22.x, I23.x, I24.1, I25.x, I46, I60.x, I61.x, I63.x, I64.x)  Self-report: 20002 (1066, 1074, 1075, 1081, 1086, 1491,1583) |
| Dementia | ICD9: 41271 (2902, 2903, 2904, 2912, 2941, 3310, 3311, 3312, 3315)  ICD10: 41270 (F00, F000, F001, F002, F009, G30, G300, G301, G308, G309, F01, F010, F011, F012, F013, F018, F019, I673, F020, G310, A810, F02, F021, F022, F023, F024, F028, F03, F051, F106, G311, G318)  Self-report: 20002 (1263) |
| Liver disease | ICD9: 41271 (571, 572, 573)  ICD10: 41270 (K70-K77)  Self-reported: 20002 (1158,1604) |
| Respiratory disease | ICD9: 41271 (491, 492, 496)  ICD10: 41270 (J40-J44, J47)  Self-reported: 20002 (1112, 1113, 1472), 6152 (6) |
| Chronic kidney disease | ICD9: 41271 (7531, 585, 586)  ICD10: 41270 (Q61, N18, N19)  Self-reported: 20002 (1427, 1192, 1193, 1194, 1519, 1520) |
| Hypertension | ICD9: 41271 (401.x-405.x)  ICD10: 41270 (I10.x-I13.x, I15.x)  Self-report: 20002 (1065, 1072, 1073)  Medication use: 6153, 6177  Systolic blood pressure ≥ 140 mmHg or diastolic blood pressure ≥ 90 mmHg |
| Type 2 diabetes mellitus | ICD9: 41271 (250.00, 250.10, 250.20, 250.90)  ICD10: 41270 (E11)  Self-reported: 20002 (1223)  Medication use: 20003  Random glucose level ≥ 11.1 mmol/L or glycated hemoglobin level ≥ 48 mmol/mol (6.5%) |
| Osteoarthritis | ICD9: 41271 (715, 721)  ICD10: 41270 (M15, M16, M17, M18, M19, M471, M472, M478, M479, M480)  Self-reported: 20002 (1131, 1465) |
| Eye disease | ICD9: 41271 (3661, 3662, 3663, 3665, 3668, 3669, 3650, 3651, 3652, 3655, 3656, 3659)  ICD10: 41270 (H25, H26, H40)  Self-reported: 20002 (1278, 1277) |
| Depression | ICD9: 41271 (311)  ICD10: 41270 (F32-F33)  Self-reported: 20002 (1286, 1531) |
| Anxiety | ICD9: 41271 (300)  ICD10: 41270 (F40-F48)  Self-reported: 20002 (1287) |

Notes: Self-reported medical history and medication information were only used to define prevalent diseases. Undiagnosed prevalent type 2 diabetes mellitus cases were identified using random glucose (≥ 11.1 mmol/L) or glycated hemoglobin (≥ 48 mmol/mol [6.5%]) levels. Undiagnosed prevalent hypertension cases were identified using systolic blood pressure (≥ 140 mmHg) or diastolic blood pressure (≥ 90 mmHg). Eye disease includes cataract and glaucoma.

Table S36. Details of the processing of modifiable factors.

| **Factors** | **Field ID** | **Description** |
| --- | --- | --- |
| **Local environment** |  |  |
| Particulate matter air pollution (pm10) | 24005, 24019 | mean value of particulate matter air pollution |
| Nitrogen dioxide air pollution | 24003, 24004, 24016, 24017, 24018 | mean value of nitrogen dioxide air pollution |
| **Psychosocial factors** |  |  |
| Able to confide | 2110 | 0: yes (about once a week, 2-4 times a week, almost daily)  1: no (never or almost never, once every few months, about once a month) |
| **Socioeconomic status** |  |  |
| Qualifications | 6138 | 0: higher education (college or University degree, other professional qualifications e.g., nursing, teaching)  1: lower education (A/AS levels or equivalent, O levels/GCSEs or equivalent, CSEs or equivalent, NVQ or HND or HNC or equivalent) |
| Current employment status | 6142 | 1: employed (in paid employment or self-employed, retired, doing unpaid or voluntary work, full or part time students)  0: unemployed (looking after home and/or family, unable to work because of sickness or disability, unemployed) |
| **Medical history** |  |  |
| Diagnosis of cardiovascular disease at baseline |  | 0: no 1: yes. Details are listed in Table S2 |
| Diagnosis of hypertension at baseline |  | 0: no 1: yes. Details are listed in Table S2 |
| Diagnosis of cancer at baseline |  | 0: no 1: yes. Details are listed in Table S2 |
| Diagnosis of diabetes mellitus at baseline |  | 0: no 1: yes. Details are listed in Table S2 |
| Diagnosis of depression at baseline |  | 0: no 1: yes. Details are listed in Table S2 |
| Diagnosis of chronic obstructive pulmonary disease at baseline |  | 0: no 1: yes. Details are listed in Table S2 |
| Attendance/disability/mobility allowance | 6146 | 0: no (none of the above)  1: yes (attendance allowance (means severely disabled), disability living allowance, blue badge (disabilities or health conditions cause immobility)) |
| **Physical measures** |  |  |
| Leg fat percentage | 23111, 23115 | mean value of left and right |
| Arm fat percentage | 23119, 23123 | mean value of left and right |
| Forced vital capacity (FVC) | 3062 | mean value of 3 arrays |
| Forced expiratory volume in 1-second (FEV1) | 3063 | mean value of 3 arrays |
| Peak expiratory flow (PEF) | 3064 | mean value of 3 arrays |
| Lung function |  | mean value of z-scores of FVC, FEV1 and PEF |
| Diastolic blood pressure, automated reading | 4079 | mean value of 2 arrays |
| Systolic blood pressure, automated reading | 4080 | mean value of 2 arrays |
| Hand grip strength | 46, 47 | mean value of left and right |
| **Lifestyle** |  |  |
| Vegetables eaten above recommendation | 1289, 1299 | 0: no 1: yes (consumption of vegetables (≥3 portions/day) |
| Fruit eaten above recommendation | 1309, 1319 | 0: no 1: yes (consumption of fruit (≥ 3 portions/day)) |
| Fish eaten above recommendation | 1329, 1339 | 0: no 1: yes (consumption of fish (daily or weekly)) |
| Processed meat intake reduced | 1349 | 0: no 1: yes (consumption of processed meat (consumed never or rarely) |
| Red meat intake reduced | 1369, 1379, 1389 | 0: no 1: yes (consumption of red meat (from never to monthly intake) |
| Wholegrain eaten above recommendation | 1438, 1448, 1458, 1468 | 0: no 1: yes (consumption of wholegrain (≥ 3 portions/day) |
| White bread/refined cereals intake reduced | 1438, 1448, 1458, 1468 | 0: no 1: yes (consumption of white bread/refined cereals (≤ 1.5 portions/day)) |
| Dietary index |  | cumulative sum of these six components (“fruit”, “vegetables”, “fish”, “red meat”, “processed meat”, “grain”) (theoretical range: 0 to 7). A score of one was assigned if participants reported increased consumption of fruit, vegetables, fish or decreased consumption of processed meat and other red meats. A score of two was assigned if participants reported increased consumption of whole grain bread and cereals, whilst a score of one was assigned if they reported decreased consumption of white bread and refined cereals. In all food groups a score of zero was assigned otherwise. |
| Healthy diet |  | 0: no 1: yes (dietary index ≥ 4) |
| Vitamin and mineral supplements (Vitamin A) | 6155 | 0: no 1: yes |
| Vitamin and mineral supplements (Vitamin B) | 6155 | 0: no 1: yes |
| Vitamin and mineral supplements (Vitamin C) | 6155 | 0: no 1: yes |
| Vitamin and mineral supplements (Vitamin D) | 6155 | 0: no 1: yes |
| Vitamin and mineral supplements (Vitamin E) | 6155 | 0: no 1: yes |
| Vitamin and mineral supplements (Folic acid or Folate (Vit B9)) | 6155 | 0: no 1: yes |
| Vitamin and mineral supplements (Multivitamins +/- minerals) | 6179 | 0: no 1: yes |
| Mineral and other dietary supplements (Fish oil (including cod liver oil)) | 6179 | 0: no 1: yes |
| Mineral and other dietary supplements (Glucosamine) | 6179 | 0: no 1: yes |
| Mineral and other dietary supplements (Calcium) | 6179 | 0: no 1: yes |
| Mineral and other dietary supplements (Zinc) | 6179 | 0: no 1: yes |
| Mineral and other dietary supplements (Iron) | 6179 | 0: no 1: yes |
| Mineral and other dietary supplements (Selenium) | 6179 | 0: no 1: yes |
| Moderate sleep | 1160 | 0: yes 1: no (sleep duration < 7 or > 9 hours) |
| Leisure/social activities | 6160 | 0: no 1: yes (sports club or gym, pub or social club, religious group, adult education class, other group activity) |

Notes: The proportion of missing values for the modifiable factors were relatively low (< 23.5%). This table only describes the details of modifiable factors which underwent processing prior to analysis.
